# Mutational mechanisms shaping the coding and noncoding genome of germinal center derived B-cell lymphomas

**DOI:** 10.1038/s41375-021-01251-z

**Published:** 2021-05-05

**Authors:** Daniel Hübschmann, Kortine Kleinheinz, Rabea Wagener, Stephan H. Bernhart, Cristina López, Umut H. Toprak, Stephanie Sungalee, Naveed Ishaque, Helene Kretzmer, Markus Kreuz, Sebastian M. Waszak, Nagarajan Paramasivam, Ole Ammerpohl, Sietse M. Aukema, Renée Beekman, Anke K. Bergmann, Matthias Bieg, Hans Binder, Arndt Borkhardt, Christoph Borst, Benedikt Brors, Philipp Bruns, Enrique Carrillo de Santa Pau, Alexander Claviez, Gero Doose, Andrea Haake, Dennis Karsch, Siegfried Haas, Martin-Leo Hansmann, Jessica I. Hoell, Volker Hovestadt, Bingding Huang, Michael Hummel, Christina Jäger-Schmidt, Jules N. A. Kerssemakers, Jan O. Korbel, Dieter Kube, Chris Lawerenz, Dido Lenze, Joost H. A. Martens, German Ott, Bernhard Radlwimmer, Eva Reisinger, Julia Richter, Daniel Rico, Philip Rosenstiel, Andreas Rosenwald, Markus Schillhabel, Stephan Stilgenbauer, Peter F. Stadler, José I. Martín-Subero, Monika Szczepanowski, Gregor Warsow, Marc A. Weniger, Marc Zapatka, Alfonso Valencia, Hendrik G. Stunnenberg, Peter Lichter, Peter Möller, Markus Loeffler, Roland Eils, Wolfram Klapper, Steve Hoffmann, Lorenz Trümper, ICGC MMML-Seq consortium, ICGC DE-Mining consortium, BLUEPRINT consortium, Ralf Küppers, Matthias Schlesner, Reiner Siebert

**Affiliations:** 1 Division of Theoretical Bioinformatics (B080), German Cancer Research Center (DKFZ), Heidelberg, Germany; 2 Department for Bioinformatics and Functional Genomics, Institute of Pharmacy and Molecular Biotechnology and Bioquant, University of Heidelberg, Heidelberg, Germany; 3 Heidelberg Institute of Stem Cell Technology and Experimental Medicine (HI-STEM), Heidelberg, Germany; 4 Computational Oncology, Molecular Diagnostics Program, National Center for Tumor Diseases (NCT), German Cancer Research Center (DKFZ) and German Cancer Consortium (DKTK), Heidelberg, Germany; 5 Institute of Human Genetics, Ulm University and Ulm University Medical Center, Ulm, Germany; 6 Intitute of Human Genetics, Christian-Albrechts-University, Kiel, Germany; 7 Interdisciplinary Center for Bioinformatics, University of Leipzig, Leipzig, Germany; 8 Bioinformatics Group, Department of Computer, University of Leipzig, Leipzig, Germany; 9 Transcriptome Bioinformatics, LIFE Research Center for Civilization Diseases, University of Leipzig, Leipzig, Germany; 10 Faculty of Biosciences, Heidelberg University, Heidelberg, Germany; 11 Bioinformatics and Omics Data Analytics (B240), German Cancer Research Center (DKFZ), Heidelberg, Germany; 12 EMBL Heidelberg, Genome Biology, Heidelberg, Germany; 13 DKFZ-HIPO, German Cancer Research Center (DKFZ), Heidelberg, Germany; 14 Department of Genome Regulation, Max Planck Institute for Molecular Genetics, Berlin, Germany; 15 Institute for Medical Informatics Statistics and Epidemiology, Leipzig, Germany; 16 Medical Faculty Heidelberg, Heidelberg University, Heidelberg, Germany; 17 Hematopathology Section, Christian-Albrechts-University, Kiel, Germany; 18 Institut d’Investigacions Biomèdiques August Pi i Sunyer (IDIBAPS), Barcelona, Spain; 19 Department of Pediatrics, University Hospital Schleswig-Holstein, Campus Kiel, Kiel, Germany; 20 University of Duesseldorf, Medical Faculty, Department of Pediatric Oncology, Hematology and Clinical Immunology, Center for Child and Adolescent Health, Düsseldorf, Germany; 21 Department of Internal Medicine/Hematology, Friedrich-Ebert-Hospital, Neumünster, Neumünster, Germany; 22 Division of Applied Bioinformatics (G200), German Cancer Research Center (DKFZ), Heidelberg, Germany; 23 Structural Biology and BioComputing Programme, Spanish National Cancer Research Centre (CNIO), Madrid, Spain; 24 Department for Internal Medicine II, University Hospital Schleswig-Holstein, Campus Kiel, Kiel, Germany; 25 Senckenberg Institute of Pathology, University of Frankfurt Medical School, Frankfurt am Main, Germany; 26 Division of Molecular Genetics, German Cancer Consortium (DKFK), German Cancer Research Center (DKFZ), Heidelberg, Germany; 27 Institute of Pathology, Charité – University Medicine Berlin, Berlin, Germany; 28 Department of Hematology and Oncology, Georg-Augusts-University of Göttingen, Göttingen, Germany; 29 Department of Molecular Biology, Radboud University, Faculty of Science, Nijmegen, The Netherlands; 30 Department of Clinical Pathology, Robert-Bosch-Hospital and Dr. Margarete Fischer-Bosch Institute for Clinical Pharmacology, Stuttgart, Germany; 31 Institute of Clinical Molecular Biology, Christian-Albrechts-University, Kiel, Germany; 32 Institute of Pathology, University of Wuerzburg and Comprehensive Cancer Center Mainfranken, Wuerzburg, Germany; 33 Department for Internal Medicine III, Ulm University, Ulm, Germany; 34 Institute of Cell Biology (Cancer Research), University of Duisburg-Essen, Medical School, Essen, Germany; 35 German Cancer Consortium (DKTK), Essen, Germany; 36 Barcelona Supercomputing Centre (BSC), Barcelona, Spain; 37 ICREA, Barcelona, Spain; 38 Institute of Pathology, Medical Faculty of the Ulm University, Ulm, Germany; 39 Present Address: University of Duesseldorf, Medical Faculty, Department of Pediatric Oncology, Hematology and Clinical Immunology, Center for Child and Adolescent Health, Düsseldorf, Germany; 40 Present Address: Computational Biology Group, Precision Nutrition and Cancer Research Program, IMDEA Food Institute, Madrid, Spain; 41 Present Address: College of Big Data and Internet, Shenzhen Technology University, Shenzhen, China; 42 Present Address: Biosciences Institute, Newcastle University, Newcastle upon Tyne, UK; 43 Present Address: Institute for Informatics, Faculty of Computer Science and Medical Faculty, University of Augsburg, Augsburg, Germany

**Keywords:** Cancer genomics, Oncogenesis, Cancer genetics

## Abstract

B cells have the unique property to somatically alter their immunoglobulin (IG) genes by V(D)J recombination, somatic hypermutation (SHM) and class-switch recombination (CSR). Aberrant targeting of these mechanisms is implicated in lymphomagenesis, but the mutational processes are poorly understood. By performing whole genome and transcriptome sequencing of 181 germinal center derived B-cell lymphomas (gcBCL) we identified distinct mutational signatures linked to SHM and CSR. We show that not only SHM, but presumably also CSR causes off-target mutations in non-IG genes. Kataegis clusters with high mutational density mainly affected early replicating regions and were enriched for SHM- and CSR-mediated off-target mutations. Moreover, they often co-occurred in loci physically interacting in the nucleus, suggesting that mutation hotspots promote increased mutation targeting of spatially co-localized loci (termed *hypermutation by proxy*). Only around 1% of somatic small variants were in protein coding sequences, but in about half of the driver genes, a contribution of B-cell specific mutational processes to their mutations was found. The B-cell-specific mutational processes contribute to both lymphoma initiation and intratumoral heterogeneity. Overall, we demonstrate that mutational processes involved in the development of gcBCL are more complex than previously appreciated, and that B cell-specific mutational processes contribute via diverse mechanisms to lymphomagenesis.

## Introduction

B-cell neoplasms encompass more than 80% of lymphoid malignancies worldwide [1]. The most common types of mature B-cell neoplasms are diffuse large B-cell lymphoma (DLBCL) and follicular lymphoma (FL), accounting for more than 50% of adult B-cell lymphomas. Both are germinal center (GC)-derived B-cell lymphomas (gcBCL). While DLBCL is a heterogeneous group of aggressive lymphomas, FL is indolent but can progress to DLBCL. DLBCL comprises two subgroups, defined by gene expression as germinal center B-cell like (GCB) and activated B-cell like (ABC), with some cases left unclassified [2, 3]. More recently, new subdivisions of DLBCL based on the patterns of mutated genes were proposed [4–7].

Lymphocytes are the only somatic cells in humans which actively alter their genomes in their physiological maturation program. Early in B-cell development, V(D)J recombination rearranges immunoglobulin (IG) genes to generate initial antigen receptor diversity. In response to T cell-dependent antigens, B cells undergo rapid proliferation in the GC [8]. Concurrently, mutations are introduced in the IG variable region genes which encode the antigen binding sites in a process called somatic hypermutation (SHM) to further diversify the IG repertoire [8]. Moreover, activated B cells can change the antibody isotype via class-switch recombination (CSR), which involves excision of a DNA fragment [9].

Both SHM and CSR are initiated by activation-induced cytidine deaminase (AID), which deaminates cytosine (C) to uracil (U) [10]. SHM introduces single nucleotide variants (SNVs) in the IG variable regions due to diverse error-prone DNA repair processes activated in response to AID activity. CSR, in contrast, is focused on the generation of DNA strand breaks into switch regions located 5’ of the IG heavy chain constant region genes (IG-switch), involving distinct factors [9].

Physiologic activity of AID is restricted to the IG loci and at much lower frequency also to a few non-IG off-targets (e.g., *BCL6*) [11]. However, AID activity also causes chromosomal translocations, and in particular in DLBCL, numerous additional genes are aberrantly targeted by SHM [12–14]. AID-mediated mutations have hence been implicated as key events in B-cell lymphomagenesis [14, 15]. Indeed, most gcBCLs exhibit oncogene translocations and recurrent targeting of B cell-specific genes by mutations ascribed to aberrant SHM [13, 14, 16]. However, a comprehensive understanding of the mutational mechanisms and genome-wide patterns in gcBCL is missing. We analyzed whole genome and transcriptome sequencing data of 181 and 176 gcBCL, respectively, in order to understand the origin and implications of somatic mutations in gcBCL. We dissect the mutational mechanisms shaping their genomes and use a comprehensive approach to elucidate how these mutate the driver genes.

## Material and methods

Sample selection, genomic and transcriptomic sequencing and bioinformatic evaluations followed the guildelines of the International Cancer Genome Consortium (ICGC) [17–20]. For details see Supplementary Methods.

## Results

### Mutational landscape

We performed whole genome sequencing of 181 pre-treatment lymphoma samples from adult patients, and 179 matching nontumor tissues using inclusion criteria described in the “Methods” section (Supplementary Table S1A). The cohort encompasses 86 FL, 17 FL/DLBCL (As FL/DLBCL cases were classified which either were composite of two compartments or in which histopathologic reviews did not yield an unambgious differentiation between both), 76 DLBCL, 1 unspecified B-cell lymphoma, and 1 lymphoma with features intermediate between DLBCL and Burkitt lymphoma (BL) (Supplementary Table S1B). Transcriptomes were obtained from 176 of the cases and used to molecularly classify them, adapting published indices [2]. We assigned 171 cases to the nonmolecular BL group, two to the molecular BL group, and three showed an intermediate profile. To increase statistical power for detecting common mutational mechanisms of B cells, these gcBCL subgroups were analyzed together in a subset of the analyses.

Whole genome sequencing data were obtained with a median coverage of 36.4 (range 24.1–56.4) and 37.0 (range 26.4–77.5) in tumors and controls, respectively, and interrogated for somatic mutations including SNVs, insertions and deletions (indels), structural variants (SVs), and copy number aberrations (CNAs). We identified a median of 8186 (range 1,236–138,620; subgroup specific median DLBCL: 12,943, FL: 5,933, FL/DLBCL: 13,381) somatic small variants (SNVs and indels) per tumor (Supplementary Fig. S1A).

A median of 55 SVs (range 2–1317; inversions: 9, deletions: 7, duplications: 26, translocations: 6) was detected per case. Most SVs were detected in FL/DLBCL (median: 100) and DLBCL (77). The number in FLs was considerably lower (35), indicating higher genomic instability in DLBCL and FL/DLBCL than in FL. The number of SVs correlated with the number of small mutations (Supplementary Fig. S1B). Regarding CNAs (deleted or gained genomic segments >1 Mb) DLBCLs (median 9 gains/5 losses) and FL/DLBCL (8/5) showed more CNAs than FLs (2/3), matching previous studies (Supplementary Fig. S1D, E) [21–23].

**Fig. 1:**
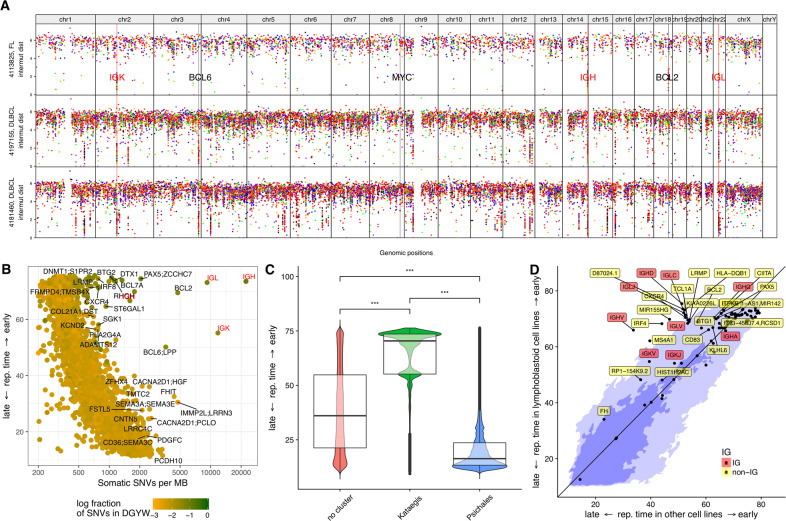
Mutation density and replication timing. **A** Rainfall plots of three samples including one FL (uppermost track) and two DLBCLs (second and third tracks from the top). For every track, the x-axis displays the genomic coordinate and the y-axis the log-scaled intermutation distance. Clusters of hypermutation (kataegis clusters) can be identified as “rainfalls” reaching very low intermutation distance. The IG loci are highlighted by red vertical lines and red labels, some hallmark genes involved in lymphomagenesis are highlighted by black vertical lines and black labels. **B**–**D** Correlation with replication timing. Replication timing is indicated as RepliSeq score of the respective genomic region as determined in [24] (see “Methods” for details). **B** Scatterplot of replication timing vs. mutation density, showing an inverse relationship between these two quantities. Outliers in this plot, i.e., exceptions from the inverse relationship, are typical targets of SHM in gcBCL. **C** Boxplot and violin plot of replication time vs. cluster category, demonstrating that kataegis is significantly enriched in early replicating regions (*p* < 10^−16^) and psichales in late replicating regions (*p* < 10^−16^) of the genome. **D** Rewiring of replication timing: kataegis regions are located in regions of the genome which are earlier replicating in lymphoblastoid cell lines (*y*-axis) than in other cell lines (HeLa-S3 (cervical adenocarcinoma), HUVEC (umbilical vein endothelial cells), K562 (chronic myelogenous leukemia in blast crisis), NHEK (epidermal keratinocytes), MCF-7 (mammary gland, adenocarcinoma), IMR-90 (fetal lung fibroblasts), and HepG2 (hepatocellular carcinoma) (*x*-axis). The light blue color in the background indicates the 95% quantile, the dark blue one the 68% quantile (respective fractions of all SNVs are situated on the colored areas). Regions with a difference in RepliSeq score > 3 are annotated by the closest gene.

SNVs exhibited a highly uneven distribution across the genome (Fig. 1A, Supplementary Fig. S2A). Cohort-wide analysis of SNV density in 1 Mb windows revealed a correlation between SNV density and replication timing [24], with higher SNV density in late replicating regions (Fig. 1B), as described [25]. However, some early replicating regions showed a very high mutation density. An increased fraction of SNVs in those windows affected the DGYW sequence motif, a preferred SHM target [26]. Many targets of physiological and aberrant SHM are located in these windows [13], e.g., *BCL2* and *PAX5* (Fig. 1B).

**Fig. 2:**
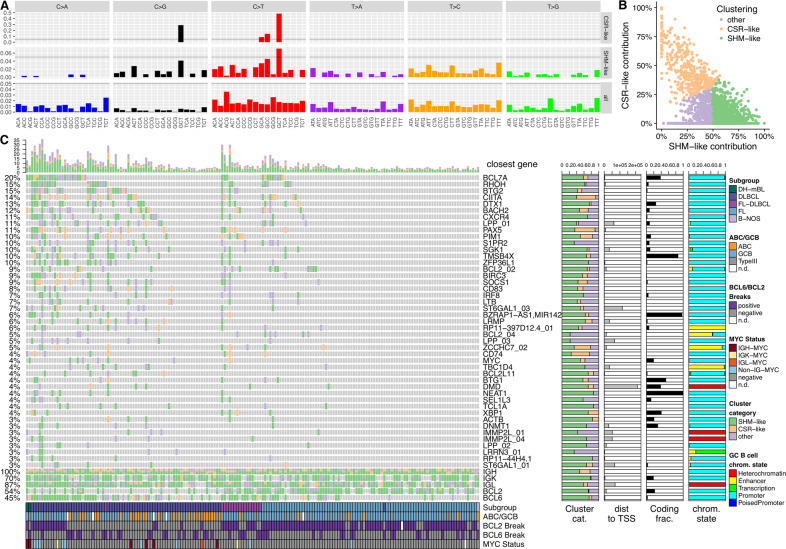
Analysis of mutation density dissects aberrant targeting of SHM and CSR. **A** Patterns of nucleotide exchanges in their triplet contexts as extracted cohort wide in the switch regions (upper track) and the regions containing V, D and J genes (middle track). These patterns are not mutational signatures, instead they correspond to visualizations of mutational catalogs. Scales on the y-axes in the different tracks are not fixed, instead a horizontal line is inserted at 5% for rough orientation and comparison. **B** Clustering of the kataegis clusters according to their contributions from CSR-like and SHM-like mutational processes with contributions of SHM-like and CSR-like as axes. Assessment of the contributions of these two mechanisms to all kataegis clusters was performed by non-negative least squares and subsequent unsupervised k-means clustering (k = 3). Kataegis clusters dominated by a CSR-like pattern are colored in orange, clusters dominated by a SHM-like pattern are colored in green and clusters dominated by neither pattern (other) are colored in purple. **C** kataegis clusters and kataegis regions displayed as oncoprint. The *x*-axis encodes samples, the y-axis the kataegis regions, which are ordered by recurrency of affection (≥3%, note that for a better overview, the well established kataegis regions in the IG, *BCL2* and *BCL6* loci are excluded from the inferred oncoprint-like ordering of the samples and only shown for completeness in the lowest five rows). The oncoprint carries four layers of annotation (normalized horizontal stacked barplots): (i) the fractions of the different kataegis cluster categories (SHM-like = green, CSR-like = orange and other = purple); (ii) the mean distance to the closest TSS in bp; (iii) the fraction of variants overlapping exons (black); and (iv) the fractions of chromatin states from GC B cells annotated to the variants in the respective kataegis regions.

Since the cohort-wide analysis masks inter-individual differences, we analyzed fluctuations in SNV density in individual genomes, excluding two cases without matched normal tissue. We identified 4538 clusters of very high mutation density (termed kataegis clusters) [27, 28] in 219 lymphoma genomes (consisting of 179 genomes from this study plus 39 pediatric BLs [17] and one adult BL, Supplementary Table S2), using a definition of a maximal intermutation distance of 1000 bp and a minimum of five mutations per cluster. Almost half of these (2,145, 47.3%) were recurrent in at least three patients and affected 166 genomic regions, which we term kataegis regions (Fig. 2 and Supplementary Fig. S3; upon omission of four hypermutated DLBCL cases, defined by more than two standard deviations above mean SNV mutational load, cf. Supplementary Information, 157 kataegis regions were identified—the difference of nine recurrent kataegis clusters contains exclusively known and established targets of SHM). 91 kataegis regions were located outside of IG loci. DLBCLs and FL/DLBCLs displayed higher median numbers of kataegis clusters, affected kataegis regions both inside the IG loci, outside the IG loci and overall as well as higher counts of SNVs in kataegis clusters (Supplementary Fig. S2B and Supplementary Table S3A). Among DLBCLs, GCB-DLBCL had higher mutational load (medians for ABC-DLBCL: 8,978 and GCB-DLBCL: 12,478), higher median numbers of kataegis clusters and affected kataegis regions, higher counts of SNVs in kataegis clusters and regions than ABC-DLBCL (Supplementary Fig. S2D and Supplementary Table S3B, C).

Beyond kataegis clusters with high mutation density, we also found regions with an intermediate mutation density, which we term psichales (ψιχάλεϛ, ancient greek for “drizzling rain”; Supplementary Figs. S2B and S4). Kataegis and psichales exhibit a remarkably different distribution over the genome, with kataegis clusters being bound to early replicating regions [24], whereas psichales is characteristic of late replicating regions (Fig. 1C, Supplementary Fig. S4). This suggests that psichales corresponds to the known increased mutation rate in late replicating heterochromatic regions [25, 29] (Supplementary Fig. S5, Supplementary Table S4) caused by differential DNA mismatch repair [30], while kataegis in gcBCL is caused by focal hypermutation of active genomic regions. Replication timing profiles differ between cell lines originating from different tissues [24]. Interestingly, kataegis clusters were enriched in genomic regions where lymphoblastoid cell lines show earlier replication than nonlymphoid cell lines (Fig. 1D). Similar to gcBCL, lymphoblastoid cell lines represent immortalized mature B cells and share with DLBCL a mature B-cell phenotype, strong proliferation, and expression of numerous B cell-typical genes. The enrichment of kataegis clusters in genomic regions where lymphoblastoid cell lines show earlier replication than the other cell lines shows that genomic regions early replicating specifically in B cells are particularly prone to become hypermutated.

### Aberrant SHM and aberrant CSR cause clusters of hypermutation

To understand the mutational mechanisms introducing the high number of kataegis clusters in gcBCL genomes we analyzed the SNV profiles at the IG loci, the physiological targets of B cell-specific mutagenesis. We derived consensus coordinates of the IG switch regions (Supplementary Figs. S6 and S7, Supplementary Information), and then extracted SNVs in the switch regions and IG V regions (IG-VDJ). Profiles of nucleotide exchanges in their triplet context (corresponding to the concept of a mutational catalog [27]) differed strongly between SNVs located in IG-switch, IG-VDJ and the overall mutational catalog (Fig. 2A). We defined the SNV profile derived from IG-switch as CSR profile and from IG-VDJ as SHM profile. The CSR profile consists almost exclusively of four triplets corresponding to a DGC/GCH motif (mutation hotspot underlined), and is therefore much more focused than the previously described RGYW/WRCY or DGYW/WRCH motifs. In contrast, the SHM profile shows a much more diverse nucleotide exchange pattern. These patterns are consistent with SNVs introduced by CSR being mainly the result of focused repair of AID-mediated C to U deamination, while SHM includes strong modulation by error-prone DNA repair pathways.

We hypothesized that kataegis outside the IG loci may be due to aberrant targeting of either SHM or CSR. Assessment of the contributions of these two mechanisms to all kataegis clusters revealed three classes of kataegis clusters (Fig. 2B): one with predominant contributions of SHM (*n* = 2,323, 51.2%), one with predominant contributions of CSR (*n* = 428, 9.4%) and one with low contributions of SHM and CSR (*n* = 1,787, 39.4%). This classification persisted when clustering only the kataegis clusters located outside IG loci with 97.6% identical assignments (Supplementary Fig. S2F). Some kataegis-regions showed strong enrichment of SHM-like kataegis clusters like those in proximity of *RHOH* and *DTX1*, whereas others, like *CIITA*, *PAX5* or *CD74*, had mainly contributions from CSR-like clusters (Fig. 2C). This suggests that beyond aberrant SHM, the mutational landscape of gcBCLs is also shaped by aberrant targeting of the CSR machinery.

Due to the fact that FL showed less kataegis clusters in total than DLBCL and FL/DLBCL, absolute numbers of CSR-like, SHM-like and “other” kataegis clusters (Supplementary Fig. S8 A, E, I) and SNVs (Supplementary Fig. S2C, items 1, 2) were lower in FL. However, when assessing the relative fraction of the respective classes of kataegis clusters among all kataegis clusters, remarkable differences were observed: while these fractions showed a trend towards lower values in FL than in DLBCL and FL-DLBCL for CSR-like (Supplementary Fig. S8 B, J) kataegis clusters and SNVs (Supplementary Fig. S2C, items 3–4), they were higher for SHM-like kataegis clusters (Supplementary Fig. S8F) and SNVs (Supplementary Fig. S2C, item 5).

### Hypermutation by proxy

SHM typically introduces mutations within a window of roughly 2.5 kb 3’ of the transcription start site (TSS). 2581/4538 (56.9%) of all kataegis clusters and 2142/2460 (87.1%) of the recurrent kataegis clusters fulfilled these criteria. However, 1056 (23.3%) of all and 39 (1.6%) of the recurrent kataegis clusters were more than 20 kb away from the next TSS (Supplementary Information). The SHM-like and CSR-like profiles were depleted among these so called “TSS-distant” kataegis clusters (Supplementary Table S5A, H and J). We annotated chromatin states computed from ChIP-Seq of three GC B-cell samples [31, 32] to the kataegis clusters (Fig. 2C). As expected, both SHM-like (1236/2321, 53%) and CSR-like clusters (317/427, 74%) were primarily located in promoters (Supplementary Table S5B). In contrast, most kataegis clusters of type “non-CSR/non-SHM-like” mapped to heterochromatin (917/1784 = 51%).

**Fig. 3:**
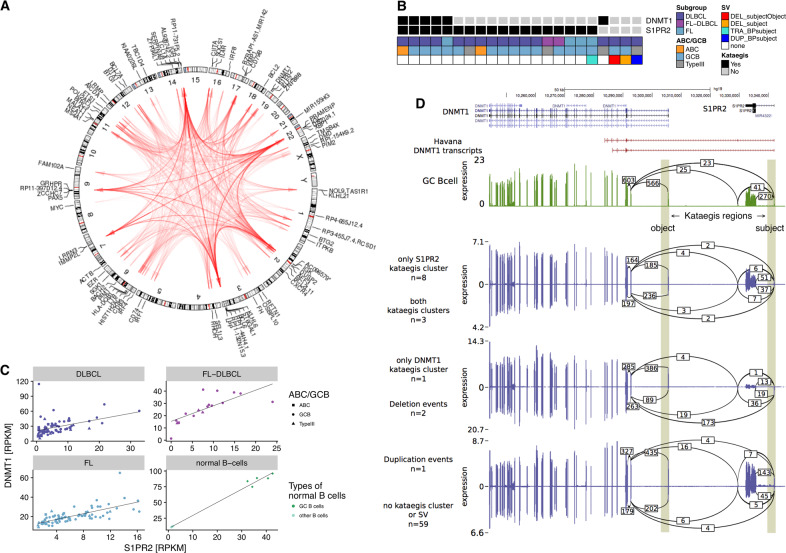
*Hypermutation by proxy* (HbP). **A** Genome-wide circos diagram showing the positions of all kataegis clusters and their co-occurrence by red arcs. The transparency of these arcs encodes the recurrency of co-occurrence. Arcs are directed from the subject (i.e., primary target) to the object (i.e., secondary target) of the HbP relationship. **B**–**D** Detailed illustration of the HbP relationship between S1PR2 and DNMT1. **B** Co-occurrence: black squares indicate in which samples kataegis clusters are present. Annotation data shows which subgroup the samples belong to, which cell of origin they have and whether a SV is present (DEL_subjectObject: deletion involving both subject (in this case S1PR2) and object (in this case DNMT1); DEL_subject: deletion involving only the subject; TRA_BPsubject: translocation with breakpoint in the subject; DUP_BPsubject: duplication with breakpoint in the subject). **C** Co-expression in the different subgroups (and normal B cells, other B cells standing for naïve B cells) and **D** tandem RNA chimeras as detected from RNA-seq: tracks displaying from top to bottom: i) known transcripts of *S1PR2* and *DNMT1;* and Sashimi plots for transcriptomic data of ii) normal GC B cells; iii) lymphoma samples with only S1PR2, i.e., the subject, affected by kataegis; iv) lymphoma samples with both S1PR2 and DNMT1, i.e., subject and object, affected by kataegis; v) lymphoma samples with only DNMT1, i.e., the object, affected by kataegis; vi) lymphoma samples with a deletion affecting either kataegis regions; vii) lymphoma samples with a duplication affecting either kataegis region; and viii) lymphoma samples affected by no event at all in this genomic region. Vertical shading highlights the genomic positions of the two kataegis regions. Numbers on arcs in the sashimi plots display the mean number of splice events (spliced reads) found in the corresponding group.

As there is indication that AID off-target activity is linked to topologically associated chromatin domains in the interphase nuclei of B cells [33], we hypothesized that TSS-distant kataegis hypermutation is caused by secondary targeting of the hypermutation machinery while primarily affecting aberrant hypermutation of target regions in spatial proximity. Hence, we systematically analyzed co-occurrence of kataegis regions (Fig. 3A). Per sample, hypermutation in certain kataegis-regions (termed *object regions*) occurred only if another kataegis-region (*subject region*) is affected (Fig. 3B, Supplementary Figs. S9, S10A, and S10F, Supplementary Table S6). Counting subject and object regions together, 77 kataegis regions outside and 16 inside the IG loci were involved in such relationships. Restricting the analysis to the 192 identified conditional co-occurrence relationships outside IG loci, 167 were inter-chromosomal, 10 were long-range intra-chromosomal (defined by a distance > 1 Mbp), and 15 were short-range intra-chromosomal effects. This suggests that the *subject regions* are primary targets of hypermutation, while the *object regions* may be exposed to the hypermutation machinery due to spatial *co-localization*. Indeed, the fraction of TSS-far kataegis regions was higher among the objects than among the subjects, regardless of whether IG loci are taken into consideration or not (Supplementary Table S7A–C). We introduced the term *hypermutation by proxy* (HbP) to describe such a relationship. Examples for subject regions include *BCL6* (Supplementary Fig. S10A–E) and *PAX5* (Supplementary Fig. S10F–I). Both *BCL6* and *PAX5* are located in gene clusters, and the HbP effect leads to secondary targeting of one or several object regions in genes within these clusters. Several objects of *PAX5* overlap with the *PAX5* enhancer described as recurrently mutated in chronic lymphocytic leukemia [34], suggesting that HbP may cause enhancer hypermutation. Another example affects *S1PR2* as subject and *DNMT1* as object (Fig. 3B–D; Supplementary Information).

In order to relate the concept of HbP to actual spatial colocalization in the nucleus, we investigated the concordance between the HbP relationships and published chromatin conformation data [35, 36] (Supplementary Table S8). Indeed, many intrachromosomal HbP relationships were reflected by strong interaction signals in the chromatin conformation data, such as gene clusters around *PAX5* and *BCL6*. However, inter-chromosomal HbP relationships could not be confirmed by the conformation data, probably because very long-range intrachromosomal and interchromosmal interactions are typically less reliably identified than short- to medium-range intrachromosomal interactions [37]. Our analysis suggests that the machinery for hypermutation has an outreach to other regions if these regions are in spatial proximity in the interphase nucleus of lymphoma cells.

Although the total number of HbP instances per sample is higher in DLBCL and FL/DLBCL than in FL (Supplementary Fig. S8M), when normalizing the number of HbP instances to the square of the number of kataegis clusters per sample (quadratic relationship between number of kataegis foci and number of HbP instances, Supplementary Fig. S8Q), FL showed higher values of this ratio (Supplementary Fig. S8N).

### New mutational signatures reflect mutagenic mechanisms active in GC B cells

**Fig. 4:**
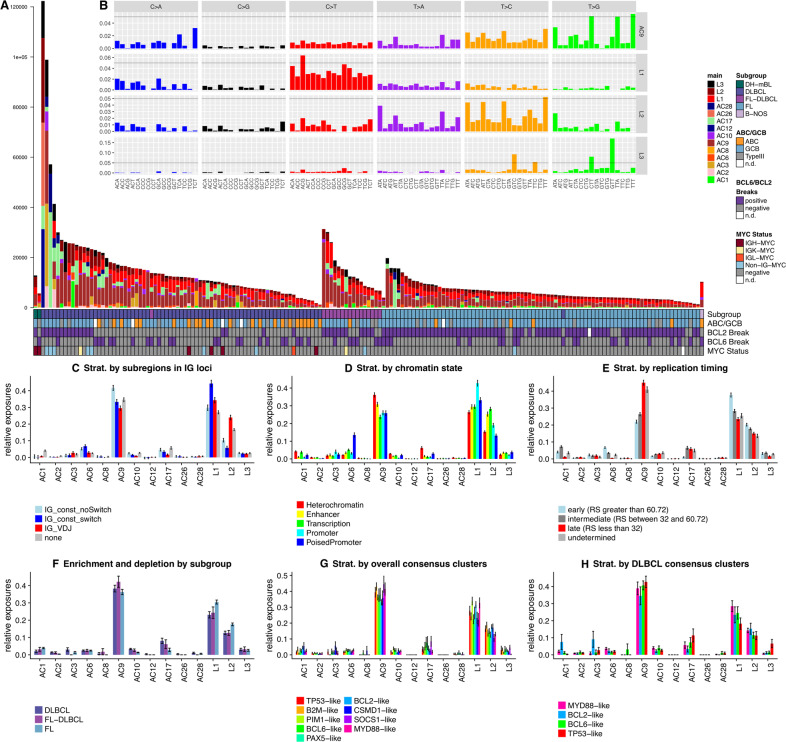
New mutational signatures are partially linked to B-cell-specifc mutagenic effects and exhibit characteristic enrichment and depletion patterns. **A** Absolute exposures of the samples to the mutational signatures extracted from the combined supervised and unsupervised analyses of mutational signatures. Heights of the stacked bar plots correspond to the number of SNVs explained by the respective mutational signatures. Samples are ordered by subgroup and then decreasing mutational load. For explanation of the identified mutational signatures please refer to the main text. (**B**, insert) 96-dimensional vectors of nucleotide exchange patterns in the triplet context for the mutational signatures AC9, L1, L2 (all of which were related to AID activity) and L3. Scales on the y-axes in the different tracks are not fixed, instead a horizontal line is inserted at 5% for rough orientation and comparison. **C**–**H** Enrichment and depletion patterns of mutational signatures by stratified analyses along different stratification axes, where different colors represent the different strata. **C** Stratification by genomic regions in which the SNVs were located (“none” = gray – outside of the IG loci, “IG_VDJ” = red – in VDJ genes or intergenic regions between these, “IG_const_switch” = blue – in the switch regions defined in this work, “IG_const_noSwitch” = light blue – in the constant domain of IGH, but outside of the switch regions). Signature L1 is enriched in the switch regions, L2 in the VDJ regions. AC9 is enriched in the constant, non-switch regions (*p*_KW_ = 2.2 × 10^−11^, *p*_Nem_ = 4.5 × 10^−6^). **D** Stratification by annotated GC B cell-specific chromatin state. L1 was enriched in promoters (*p*_KW_ = 1.2 × 10^−15^, *p*_Nem_ = 5.9 × 10^−14^), while L2 was enriched in transcribed regions (*p*_KW_ = 7.7 × 10^−30^, *p*_Nem_ = 4.7 × 10^−14^) and enhancers (*p*_Nem_ = 1.4 × 10^−8^) as compared to heterochromatic regions. **E** Stratification by replication timing, illustrating a rewiring of this measure: L1 showed a strong (*p*_KW_ = 2.7 × 10^−19^, *p*_Nem_ = 5.3 × 10^−14^, fold change FC = 1.606) and L2 a moderate (*p*_KW_ = 5.7 × 10^−11^, *p*_Nem_ = 5.6 × 10^−6^, FC = 1.347) enrichment in early replicating regions, as opposed to AC9 which is enriched in late replicating regions (*p*_KW_ = 6.3 × 10^−51^, *p*_Nem_ < 2 × 10^−16^). RS: RepliSeq score. **F** Enrichment and depletion patterns by subgroup of gcBCL: FLs had higher contributions of L1 (*p*_KW_ = 4.74 × 10^−3^, *p*_Nem_ = 1.2 × 10^−3^), L2 (p_KW_ = 6.51 × 10^−4^, *p*_Nem_ = 1.7 × 10^−4^) and AC1 (*p*_KW_ = 1.21 × 10^−5^, *p*_Nem_ = 1.3 × 10^−6^) but lower contributions of AC17 (*p*_KW_ = 2.02 × 10^−3^, *p*_Nem_ = 1.1 × 10^−3^), AC10 (*p*_KW_ = 6.51 × 10^−3^, *p*_Nem_ = 2.1 × 10^−4^), AC6 (*p* = 2.05 × 10^−2^) and AC2 (*p*_KW_ = 8.93 × 10^−3^, *p*_Nem_ = 1.3 × 10^−2^) as compared to DLBCLs. **G** Stratification by consensus clustering of the whole gcBCL cohort. While L3 (*p*_KW_ = 9.78 × 10^−3^, enriched in the SOCS1-like, B2M-like and TP53-like consensus clusters), AC1 (*p*_KW_ = 9.78 × 10^−3^, enriched in the CSMD1-like and BCL2-like consensus clusters) and AC2 (*p*_KW_ = 4.88 × 10^−2^, depleted in the BCL2-like cluster) were significantly enriched or depleted between the consensus clusters, L1 (high in the PIM1-like, BCL2-like and MYD88-like consensus clusters) and L2 (high in the B2M-like, BCL2-like and CSMD1-like consensus clusters) only showed trends. **H** Stratification by consensus clustering of only the DLBCL subgroup. After correcting for multiple testing, no significant effect was observed, with trends for L3 (high in TP53-like) and AC1 (high in BCL2-like). Error bars in **C**–**H** display standard error of the mean (SEM).

We investigated mutational signatures as traces of mutational mechanisms active in tumors [27]. We used 2,133,341 somatic SNVs from 219 lymphomas from the extended cohort defined above to perform a combination of unsupervised and supervised analyses of mutational signatures and found 14 different signatures (Fig. 4, Supplementary Figs. S11, S12, Supplementary Table S9). Of those, 11 (labeled “AC”) have been described before [27], including four of six signatures previously identified in gcBCL (Supplementary Table S9). Three new signatures were discovered, termed L1, L2, and L3 (Fig. 4A, B). Two of six mutational signatures from the original analysis of gcBCL were not identified in this analysis: AC13 (linked to the action of APOBEC enzymes) and AC5 (related to the age of the patients at diagnosis, mechanism unknown). Signature AC5 has high cosine similarity (see Methods) to L1, L2, and AC9. Because of this high similarity, most mutations we assign to L1 and L2 would have been assigned to AC5, if AC5 was included and L1 and L2 were not included in the analysis. Among the previously not extracted signatures is AC3, which we detected in 21 lymphomas. Signature AC3 has been linked to defects in homologous recombination repair (BRCAness) [38] which potentially confer synthetic lethality to poly(ADP-ribose) polymerase inhibitors [39].

To relate B cell-specific mutational processes to the new mutational signatures we compared all 14 signatures to the AID target motif DGYW [40] and to our CSR and SHM profiles by cosine similarity. L1 showed the highest similarity to DGYW and to the CSR profile, while L2 showed the highest similarity to the SHM profile. Signature L3 may have some link to APOBEC enzyme activity. With increasing factorization ranks in NMF, L3 splits apart from AC2 (an APOBEC signature) at rank 7 (Supplementary Fig. S11). Hence, the mutational mechanism causative for L3 remains presently unclear. In a complementary approach, we compared the extracted mutational signatures to a synthetic mutational signature based on data by Yaari et al. [41], who extracted synonymous mutations from V and J genes of the IGH locus from normal B cells in their 5-mer sequence context to obtain the fingerprint of physiologic SHM. We aggregated these into 3-mer context and used the resulting triplet frequencies to derive a synthetic SHM signature. Again, the newly identified signature L2 had highest similarity to the synthetic SHM signature, providing further evidence for SHM being the mechanism behind L2.

Several mutational processes show varying activity in distinct genomic regions [42], and in particular for the B cell-specific mechanisms a strong preference of certain target regions is known [43]. We stratified SNVs according to different genomic features and performed supervised analysis of mutational signatures (Fig. 4C–E, Supplementary Table S10). First, to relate mutational signatures to the physiological sites of B-cell mutagenesis, we checked for enrichment and depletion patterns in the IG-VDJ genes and the switch regions (Fig. 4C). L1 was enriched in the switch regions while L2 was enriched in the VDJ regions, corroborating our previous assignment. Second, we related the mutational signatures to chromatin states from normal GC B cells (Fig. 4D). L1 was enriched in promoters, while L2 was enriched in transcribed regions and enhancers as compared to heterochromatic regions, consistent with previous observations that B cell-specific mutagenesis primarily affects active regions of the genome [44]. Third, we assessed the influence of replication timing on exposure to the mutational signatures (Fig. 4E, Supplementary Fig. S10C). L1 showed a strong and L2 a moderate enrichment in early replicating regions. Strikingly, AC9, which has exclusively been found in B-cell malignancies and described as being linked to SHM [27] shows an enrichment in the heterochromatic, late replicating regions. In the IG loci, AC9 is enriched in the constant, non-switch regions, further corroborating that AC9 is not the fingerprint of SHM or CSR. As expected, L1 and L2 were enriched in kataegis clusters, with L1 being enriched in CSR-like and L2 in SHM-like kataegis clusters as compared to the nonclustered SNV stratum (Supplementary Fig. S12G). FLs had higher contributions of L1, L2, and AC1 but lower contributions of AC17, AC10, AC6, and AC2 as compared to DLBCLs (Fig. 4F).

We propose that L1 and L2 are the mutational footprints of CSR and SHM, respectively. The etiology of the B cell-specific signature AC9 and the new signature L3 remain enigmatic, though L3 may have some link to APOBEC activity.

### Mutational mechanisms during lymphoma evolution

To dissect the activity of the different mutational processes during B-cell lymphoma evolution, we stratified SNVs according to their cancer cell fractions (CCFs), i.e., the fraction of tumor cells harboring the respective variant. A high CCF identifies mutations which arose in the precursor cell or early in tumor evolution, while a low CCF is characteristic for mutations which arose late in tumor evolution (see Methods). Stratified analysis of mutational signatures showed an enrichment of AC1 (spontaneous deamination) and AC2 (APOBEC) in early clonal evolution (Supplementary Fig. S12D). Among the mutational signatures related to B cell-specific mutational processes, L1 showed a trend towards enrichment in early and AC9 in late clonal evolution. No enrichment was observed for L2. Following the hypothesis that the absence of enrichment patterns for L2 might indicate ongoing SHM activity in gcBCL, we investigated the distribution of CCFs in the IG loci. SNVs in the constant part of IGH were significantly earlier and SNVs in the variable parts of the IG loci were significantly later in clonal evolution than SNVs outside of the IG loci (Supplementary Fig. S12B). Hence, SHM in the variable parts of the IG loci is ongoing in gcBCL, while CSR appears to happen mostly before clonal expansion, in agreement with the genome-wide enrichment patterns for L1 (CSR) and L2 (SHM).

### Drivers of gcBCL

**Fig. 5:**
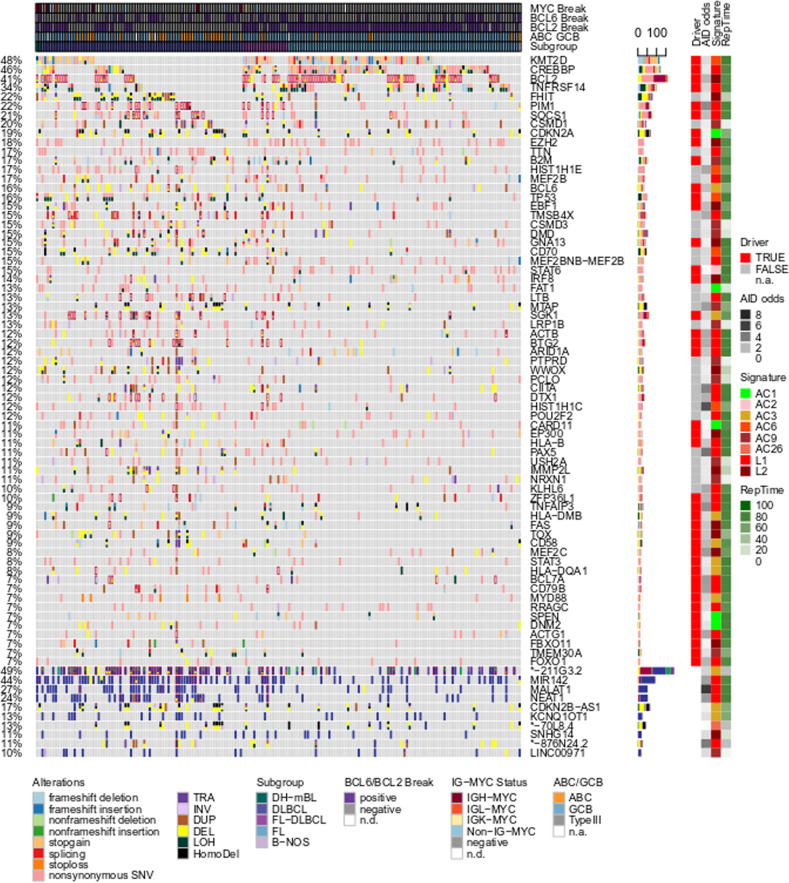
B cell-specific mutagenesis alone is not sufficient to drive lymphomagenesis. Oncoprint of coding (upper part of the figure) and noncoding (lower fifth of the figure dominated by blue color) mutations. The *x*-coordinate encodes samples which are pre-sorted by subgroups. The *y*-coordinate encodes different genes or non-coding genes. Different mutation types are encoded by the fill color of the fields in the oncoprint, where different types of mutation can coexist in one sample. Four layers of annotation on the right side of the oncoprint display (i) whether a gene is identified as a driver and (ii) how strongly mutations in AID-specific motifs are enriched, (iii) the best matching signature, and (iv) replication timing.

Only roughly 1% of somatic mutations were in protein coding sequences, with a median of 88 coding variants per sample (range 11–974, subgroup specific median DLBCL: 114, FL: 59.5, FL/DLBCL: 128; Supplementary Fig. S1, Supplementary Table S3). After integrating all types of variants with coding potential, we observed high mutational recurrence in known gcBCL drivers like *KMT2D*, *CREBBP*, *BCL2*, *TNFRSF14*, *PIM1*, *SOCS1*, and *CDKN2A* (Fig. 5, Supplementary Figs. S13, S14; see Supplementary Information for recurrently mutated noncoding genes). To differentiate between passenger and driver mutations and to identify subgroup-specific low recurrence drivers we applied IntOGen [45] to the whole cohort and to FL and DLBCL separately. We identified 118 driver genes in the 179 gcBCL with matched normal control (Supplementary Table S11), of which 9 and 8 were not significant in FLs (*ADAMTS1*, *ANKRD12*, *DHX16*, *DNM2*, *LRP12*, *SIAH2*, *SIN3A*, *ZNF217*, *ZNF292*) and not significant in DLBCLs (*BCL2*, *CDC42BPB*, *CXCR4*, *DHX15*, *JUP*, *MGEA5*, *MYCBP2*, *PDS5B*), respectively.

Encouraged by recent studies proposing genomic classifications of DLBCL based on data from whole exome sequencing [4–6] we applied NMF as a soft clustering technique on binarized data of driver gene alterations, both to the subset of DLBCL in our cohort (initially 76 cases, but 72 after excluding four hypermutated cases, defined by mutational load more than two standard deviations above mean SNV mutational load), and to the whole cohort. As described in the Supplementary Information and shown in Supplementary Fig. S15 this yielded consensus clusters comparable to the prior studies, which supports the validity of our approach for driver gene identification from the whole genome sequences. Notably, when we extended the approach from DLBCL to the full cohort (again excluding the four hypermutated DLBCLs), the optimal number of consensus clusters was nine, thereby revealing a more detailed substructure of gcBCL entities than in the published studies (Supplementary Fig. S16). We furthermore investigated congruence and cross-over of the DLBCL cases between the consensus clusters extracted only among the DLBCLs (Supplementary Fig. S15) and those consensus clusters extracted among all gcBCL cases (Supplementary Fig. S16), showing that the majority of cases in the MYD88-like and TP53-like DLBCL-only consensus clusters also mapped to the respective gcBCL consensus clusters, whereas cases from the BCL2-like DLBCL-only consensus cluster also populated the CSMD1-like gcBCL consensus cluster and cases from the BCL6-like DLBCL-only consensus cluster also populated the PIM1-like gcBCL consensus cluster. Numbers are displayed in Supplementary Table S12C. We then took these consensus clusters and investigated enrichment and depletion patterns of the mutational signatures identified in our analysis (Fig. 4G, H). L3 was enriched in the SOCS1-like, B2M-like and TP53-like consensus clusters, AC1 was enriched in the CSMD1-like and BCL2-like consensus clusters, AC2 was depleted in the BCL2-like cluster, L1 showed a trend and was higher in the PIM1-like, BCL2-like and MYD88-like consensus clusters compared to background, and L2 showed a trend and was higher in the B2M-like, BCL2-like, and CSMD1-like consensus clusters compared to background (Fig. 4G). Stratification by consensus clustering of only the DLBCL subgroup (Fig. 4H) revealed only trends for L3 (high in TP53-like) and AC1 (high in BCL2-like).

We sought to identify the mechanisms mutating the driver genes as well as other recurrently mutated genes. To assess the contribution of B cell-specific hypermutation, we mapped kataegis clusters to driver genes and found that 57.1% of the driver or recurrently mutated genes showed indications for kataegis in at least one case (36.4% when restricting the analysis to coding mutations, Supplementary Table S13). Complementarily, 42.9% of the driver and recurrently mutated genes were depleted in mutations affecting the DGC/CGH motif, indicating non-AID-mediated mutagenesis (Fig. 5, Supplementary Fig. S14). While genes that were recurrently affected by kataegis generally showed an enrichment of SNVs in the DGC motif, the reverse relation was often not fulfilled, suggesting that several genes are recurrently targeted by AID-mediated, but nonclustered mutations.

Next we compared cohort-wide mutational profiles for each driver and recurrently mutated gene with the previously identified mutational signatures using cosine similarity. 37.7% of the driver genes exhibited a profile most similar to signature L1 and 18.2% to L2, while 15.6% were most similar to AC9 (Fig. 5). Several drivers showed no evidence for B cell-specific mutagenesis, i.e., no enrichment for the AID target motif, no kataegis, and no predominant mutagenesis by a B cell-specific signature. Examples are *TP53* and *CARD11* with a pattern of SNVs dominated by signatures AC1 and AC6 (associated with defects in DNA mismatch repair).

Finally, we investigated the timing of coding driver mutations in the course of lymphoma evolution. We determined the median CCF per driver gene and ranked the genes accordingly to classify driver genes as early or late (Supplementary Fig. S17). In agreement with our previous analyses, early drivers were predominantly mutated by L1, whereas for intermediate and late drivers L2 and AC9 were the dominating signatures. Genes affecting NFκB signaling (*PPP4C*, *NFKBIE*, *NFKBIA*) [46–48] were mutated early during clonal evolution, suggesting that activation of NFκB signaling is essential for initiation of B-cell lymphomagenesis.

## Discussion

Most B-cell lymphomas derive from GC B cells [15]. Considering that most newly generated B cells will never participate in a GC reaction during their lifetime, and that those which do will be GC B cells only for a short time of about three weeks [49], and then continue to live as memory B cells or plasma cells for years or decades in humans, it becomes evident that the GC is a highly dangerous place for B cells. Key factors that contribute to the risky life of GC B cells are (i) the very high proliferation rate of GC B cells [50], which increases the risk for DNA replication-associated genetic lesions and may prepare the cells for continuous proliferation as transformed cells, (ii) the generation of chromosomal translocations as mistakes of SHM and CSR [16], (iii) off-target mutation activity of SHM [13, 14], and iv) a dampened DNA repair activity needed to tolerate the genotoxic stress imposed on GC B cells by their fast proliferation and SHM activity [51]. Moreover, B cells can repeatedly undergo GC reactions, and this repeated exposure to the mutagenic GC microenvironment may indeed play a role in FL pathogenesis [52]. However, a comprehensive understanding of the mutagenic mechanisms causing the malignant transformation of GC B cells is still missing. By analyzing a large number of prototypical gcBCL for mutations not only in the coding but also the noncoding genome we were in a position to study mutational mechanisms in gcBCL at unprecedented depth.

One of the major findings from our study is that besides kataegis regions of very high mutational density, the lymphomas also show recurrent regions of psichales with an intermediate mutation density. The observation that kataegis regions mostly affect early replicating genomic regions, while psichales focusses on late replicating regions, points to the involvement of distinct mutational mechanisms. Indeed, the distinct mutation patterns in kataegis and psichales clusters suggest a major role of off-target AID activity for kataegis, and of diminished DNA repair activity in late replicating regions of psichales clusters. The GC-dependency of kateagis regions is supported by a recent study published during the review process of this paper which reported that IGHV gene unmutated chronic lymphocytic leukemias lack kataegis regions outside the IGH switch regions [53]. The increased mutation density in late replicating regions is not B cell-specific and known from other types of cancer [25, 29]. A second novel mutation feature that we uncovered is *hypermutation by proxy*. This describes the surprising observation that some kataegis clusters generated by strong hypermutation activity can apparently promote hypermutation in other loci if co-localized in the nucleus. Hence, accumulation of hypermutation complexes on particular genomic regions apparently poses the risk to also mutagenize spatially closely localized chromosomal regions in trans. This concept is supported by a recent lymphoma cell line study showing hot spots for SHM in topologically associated chromatin domains, although that study lacked the aspect of directionality that we revealed [54]. Third, while prior studies on off-target AID activity only considered off-target SHM [13], we revealed that also the mutation machinery involved in CSR apparently has off-target mutation activity beyond inducing translocations and contributes to the SNV burden of gcBCL. Please note that the two AID-associated signatures we describe here are distinct from the canonical and noncanonical AID signatures reported previously [27, 55, 56]. Whereas the canonical AID signature is a general AID signature based on the AID hotspot motif, not distinguishing SHM and CSR machinery associated mutagenesis, the noncanoncial AID signature (signature 9 in ref. [27]) is indeed primarily linked to polymerase eta mutagenesis, and not AID directly [27, 55, 56]. Fourth, overall, about half of the gcBCL driver genes show signs of targeting by B cell-specific mutational processes, and the resulting mutations likely play a major pathogenetic role both in the initiation of lymphomagenesis and in the generation of intratumoral heterogeneity. Fifth, using NMF consensus clustering on data integrating various mutation types across the different gcBCLs, we identified nine consensus clusters corresponding to genomic subtypes. In conclusion, the development of gcBCL is much more complex than previously appreciated and gcBCL are unique among human cancers in the extent and diversity of how cell-type-specific processes contribute to mutations, localized hypermutation and malignant transformation.

## Supplementary information

Supplementary Methods

Supplementary Information

Supplemental Figures

Supplementary Tables 1–5, 7, 9–13

Supplemental Table 6

Supplemental Table 8
