# Supplementary material for: Mutational mechanisms shaping the coding and noncoding genome of germinal center derived B-cell lymphomas
Source: Leukemia. 2021 May 5;35(7):2002–16. doi: 10.1038/s41375-021-01251-z (PMC8257491; doi:10.1038/s41375-021-01251-z)
Supplement: Supplementary file 1 — Supplementary Methods [file 41375_2021_1251_MOESM1_ESM.pdf]

Hübschmann et al., Mutational mechanisms shaping the coding and non-coding genome of germinal center derived B-cell lymphomas

Supplemental Methods

Methods and procedures described herein were developed by the MMML and ICGC MMML-Seq consortia. They partially overlap with those described in a study on pediatric Burkitt lymphomas [1].

Study cohort

The ICGC MMML-Seq cohort comprises pre-treatment tumor tissue and corresponding matched normal material (peripheral blood, buffy coats shown to be tumor free by clonality analyses) obtained with informed consent of the respective patients and/or their legal guardians in the case of minors. The ICGC MMML-Seq study was approved by the Institutional Review Board of the Medical Faculty of the University of Kiel (A150/10) and of the recruiting centers. In all tumor samples, basic characterization including histopathological panel review and immunohistochemical and FISH analyses was performed as described recently [2].

From the overall ICGC MMML-Seq cohort a total of 181 samples of gcBCL (DLBCL, FL, DLBCL-FL, DHL, and B-NOS) entered this study and passed quality control criteria. The inclusion criteria were based on diagnosis of gcBCL according to the WHO 2008 criteria [3] and age at diagnosis of ≥ 30 years. Corresponding matched normal tissues for WGS was available in 179 of the 181 patients while RNA-Seq data was available for 176 patients (Suppl. Table 1).

In addition, FACS-sorted germinal center (GC) B cells from non-neoplastic tonsils were analyzed. For the isolation of GC B cells, first, B cells were enriched from tonsillar specimens by magnetic-activated cell sorting (MACS) with anti-CD19-microbeads (Miltenyi Biotech, Bergisch Gladbach, Germany), followed by fluorescence-activated cell sorting of $CD20^{high}CD38^{+}$ GC B cells on an ARIA3 cell sorter (Becton Dickinson, Heidelberg, Germany).

Sample processing

The study was performed in accordance with the ICGC guidelines (www.icgc.org). The experimental procedures for DNA and RNA extraction and the detection of IG rearrangements have been published previously [2,4].

Sequencing

DNA libraries of the tumor and their matched control samples were prepared using the TruSeq DNA library Preparation kit Sets A and B (Illumina; estimated insert size of 343 bp) or the TruSeq Nano DNA library Preparation kit (Illumina; estimated insert size of 350 bp). Clusters were generated with cBot and the TruSeq PE Cluster kit v3 cBot HS (15023336_A, Illumina). Paired-end sequencing was performed on Illumina HiSeq2000 (2x 100 bp), HiSeq2500 (2x 100 bp) or Hiseq X (2x 150 bp) instruments using the TruSeq SBS kit, Version 2.5 (200 cycles).

RNA libraries of the tumor samples and sorted GC B cells were generated using the TruSeq RNA library preparation Kit Set A and B, following manufacturer´s instructions at an insert size of ~300 bp. Two barcoded libraries were pooled per lane and sequenced on Illumina HiSeq2000, or HiSeq2500.

Whole genome sequencing data processing

Alignment

Read pairs were mapped to the human reference genome (build 37, version hs37d5), using bwa mem [5] (version 0.7.8 with minimum base quality threshold set to zero [-T 0] and remaining settings left at default values), followed by coordinate-sorting with bamsort (with compression option set to fast (1)) and marking duplicate read pairs with bammarkduplicates (with compression option set to best (9)); both are part of biobambam package version 0.0.148.

SNV and indel calling

Somatic SNVs and indels in matched tumor normal pairs were identified using the DKFZ core variant calling workflows of the ICGC PCAWG project (https://dockstore.org/containers/quay.io/pancancer/pcawg-dkfz-workflow) as follows. Somatic SNVs were identified with the DKFZ SAMtools-based calling pipeline [6,7]. Initial candidate variants for SNVs in the tumor were generated by samtools and bcftools (version 0.1.19), followed by a lookup of the corresponding positions in the control. To enable calling of variants with low allele frequency we disabled the Bayesian model (by setting -p 2). Thus, all positions containing at least one high quality non-reference base are reported as candidate variant. The resulting raw calls were categorized into putative somatic variants and others (artifacts, germline) based on the presence of variant reads in the matched normal sample. The frequency of all putative somatic variants was then refined by checking for potential redundant information due to overlapping reads and precise base counts for each strand were determined. All variants were annotated with dbSNP141, 1000 Genomes (phase 1), Gencode Mapability track, UCSC High Seq Depth track, UCSC Simple-Tandem repeats, UCSC Repeat-Masker, DUKE-Excluded, DAC-Blacklist, UCSC Selfchain. The confidence for each variant was then determined by a heuristic punishment scheme taking the aforementioned tracks into account. In addition, variants with strong read biases according to the strand bias filter were removed. High confidence variants were used for further analysis.

Tumor and matched control samples were analyzed by Platypus (version 0.8.1) [8] to identify indel events. All variants indicating an indel were categorized into putative somatic and other based on the genotype likelihoods (matched genotype 0/0 for somatic indels). High confidence somatic variants were required to either have the Platypus filter flag PASS or pass custom filters allowing for low variant frequency using a scoring scheme. Candidates with the badReads flag, alleleBias, or strandBias were discarded if the variant allele frequency was <10%. Additionally, combinations of Platypus non-PASS filter flags, bad quality values, low genotype quality, very low variant counts in the tumor, and presence of variant reads in the control were not tolerated. In order to remove recurrent artifacts and misclassified germline events, somatic indels that were identified as germline in at least two patients in the ICGC MMML-seq cohort were excluded.

Samples without matched control were processed with modified versions of both workflows as described in Jabs *et al.* [9]. In addition, variants exceeding an allele frequency of 0.0001 in ExAC v.0.3.1 (non-TCGA variants) and variants showing indications for a base quality bias or a mapping quality bias (corresponding samtools mpileup PV4 p-value<0.01) were excluded from analysis. Finally, variants in artifact-prone regions were removed. These artifact-prone regions were identified in an in-house lymphoma exome cohort based on their mutation density. Regions with 2-4 variants with maximum intermutation distance of 100 bp were selected. Recurrently affected regions (>2 patients) were considered artifacts, unless these regions overlapped with recurrent kataegis regions defined by a cohort of 219 gcBCL (179 cases with matched normal from this study and additional 40 pediatric BLs, 39 from ref. [1] and one adult BL) referred to as *merged non-BL/BL cohort*.

For four samples (4117030, 4138464, 4177175, 4133863) tumors and their matched controls were sequenced on different Illumina instruments (Hiseq2500 and HiseqX). To prevent technology-specific artefacts, the standard SNV calling workflow was extended with filters from the no control workflow [9]. Somatic small variants were further filtered out if their respective position was covered insufficiently in the control sample (<20X) or if the fraction of variant reads in the control was too high (>1/30).

For some samples increased artefact rates were detected which were related to higher base quality scores for wrongly called bases. For these samples the base quality threshold was increased from 13 to 20 and low mutant allele frequency (MAF) penalty was switched off (i. MPILEUP_OPTS="-REI -Q 20 -q 30 -ug"; CONFIDENCE_OPTS=" -c 0 -l 1") (4100314, 4100636, 4103434, 4103570, 4107990, 4108588, 4116268, 4119463, 4121263, 4123945, 4124795, 4131738, 4144131, 4144366, 4146136, 4148261, 4152036, 4158933, 4160069, 4162154, 4163741, 4166940, 4167381, 4169012, 4170577, 4170844, 4177175, 4177639, 4178518, 4180106, 4190231, 4190316, 4192483, 4193435, 4193646, 4197438, 4199848). One of these samples (4158268) additionally showed signs of strong guanine oxidation and was processed with base quality threshold 20 and low MAF penalty switched on (MPILEUP_OPTS="-REI -Q 20 -q 30 -ug"; CONFIDENCE_OPTS=" -c 0").

SNVs and indels from all samples were annotated using ANNOVAR [10] according to GENCODE gene annotation (version 19) and overlapped with variants from dbSNP [11] (build 141) and the 1000 Genomes Project database.
Statistics over coding small variants were estimated based on all 181 cases while the two samples without matched control were excluded for non-coding variant and SV statistics, mutation density analysis, mutational signature analysis and driver identification.

Detection of genomic structural rearrangements
Genomic structural rearrangements were detected using SOPHIA v.34.0 (manuscript in preparation) as described in Sahm *et al*. [12] and DELLY [13] as described in Northcott *et al*. [14]. Briefly, SOPHIA uses supplementary alignments as produced by bwa-mem as indicators of a possible underlying SV. SV candidates are filtered by comparing them to a background control set of sequencing data obtained using normal blood samples from a background population database of 3261 patients from published TCGA and ICGC studies and both published and unpublished DKFZ studies, sequenced using Illumina HiSeq 2000, 2500 (100 bp) and HiSeq X (151 bp) platforms and aligned uniformly using the same workflow as in this study. An SV candidate is discarded if (i) it has more than 85% of read support from low quality reads; (ii) the second breakpoint of the SV was unmappable in the sample and the first breakpoint was detected in 10 or more background control samples; (iii) an SV with two identified breakpoints had one breakpoint present in at least 98 control samples (3% of the control samples); or (iv) both breakpoints have less than 5% read support. Statistics over SVs for 179 samples with matched control and integrated variant analysis over all samples were based on SOPHIA calls.

Detection of copy number aberrations and allelic imbalances
Allele-specific copy-number aberrations were detected using ACEseq (allele-specific copy-number estimation from whole genome sequencing) [15]. ACEseq determines absolute allele-specific copy numbers as well as tumor ploidy and tumor cell content based on coverage ratios of tumor and control as well as the B-allele frequency (BAF) of heterozygous single nucleotide polymorphisms (SNPs). SVs called by SOPHIA were incorporated to improve genome segmentation. Samples without matched control were processed using the ‘runWithoutControl=true’ option.
Ploidies were manually checked and compared with FISH results. Adjustments were made if necessary. Accordingly, tumor cell content estimates were compared to the doubled median MAF and adjusted in case ACEseq and MAF-based estimates deviated by more than 10% from each other.
To prevent biases due to oversegmentation, copy number profiles were further smoothed prior to calculating the total number of gains and losses. Neighboring segments were merged if they rounded to the same copy number and deviated by less than 0.5 copies in case of segments <20 kb or deviated by less than 0.3 copies otherwise. Remaining segments <500 kb were merged with their closer neighbor based on allele-specific and total copy number and once again segments smaller than 2 Mb deviating by less than 0.4 copies were merged. Based on the resulting segments the number of gains and losses was estimated.

Cancer cell fractions (CCFs)
To obtain clonality estimates of small variants, CCFs were computed. Even though the possibilities to analyze the allele frequency of single mutations are limited at a median coverage of 36.41 as reached in this study, the determination of an enrichment and depletion pattern in a stratified analysis of mutational signatures is a technique based on ensembles or large sets of mutations in the different strata (Suppl. Table 10). The analyzed signal which then provides the enrichment and depletion pattern stems from the entirety of the signals of all mutations in the respective stratum. The uncertainty of a value averaged over a whole ensemble is much smaller than the uncertainty of a value assigned to one specific mutation.
First, we estimated the corrected MAF for each SNV and indel based on the tumor cell content (tcc) and the copy number of the corresponding segment:

$$MAF_{corrected} = \frac{reads_{alternateAllele}}{TCN * tcc} \times reads_{total} \times \ (TCN \times tcc + TCN_{normal} \times (1 - tcc) \qquad (1)$$

Reads indicates the number of reads, while TCN is the total copy number in the tumor and TCN_{normal} the copy number in the control sample, i.e. 2 and in case of male patients 1 for chromosome X and Y. Second, we estimated the CCF for all SNVs and indels on segments with one or two copies, excluding segments with higher or subclonal copy numbers. For SNVs and indels in heterozygous diploid segments the corrected MAF was multiplied by two to obtain the CCFs. A binomial test was performed for mutations mapping to diploid segments with loss of heterozygosity to determine whether the event most likely occurred before or after the duplication of the remaining allele. We tested under the null hypothesis that the event occurred after the duplication and therefore used an event probability of 0.5*tcc. The corrected MAF was multiplied by two if the null hypothesis was not rejected (p-value <0.05). Otherwise, we assumed that the corrected MAF represented the CCF already, as was done for mutations on segments with a single copy.

Repli-Seq scores as a measure of replication timing

Repli-Seq scores [16] were used to assess the replication timing of mutations. Replication timing of lymphoblastoid cell lines was calculated as median Repli-Seq score of the lymphoblastoid ENCODE cell lines GM12801, GM06990, GM12812, GM12813, and GM12878. Replication timing of “other” cell lines was the median score from the ENCODE cell lines HeLa-S3, HUVEC, K562, NHEK, MCF-7, IMR-90, and HepG2.

ChIP-seq of normal GC B cells

Data. The raw sequencing read data is publicly available from the Blueprint DCC portal (http://dcc.blueprint-epigenome.eu/#/home). The identifiers are listed in Table 1 below:

Table 1: ChIP-seq of normal GC B cells

**Sample ID**	S00W0DH1	S00Y9OH1	S013ARH1
**Cell Type**	gcBC	gcBC	gcBC
**Donor ID**	T14_5	T14_10	T14_11
**Donor Age (yrs)**	3-10	5-10	0-5
**Sex**	F	F	M
**Tissue**	tonsil	tonsil	tonsil
**Input**	ERX941037	ERX943225	ERX1007400
**H3K27ac**	ERX712687	ERX712724	ERX1007385
**H3K27me3**	ERX712690	ERX712727	ERX1007399
**H3K36me3**	ERX712688	ERX712725	ERX1007386
**H3K4me1**	ERX712694	ERX712723	ERX1007405
**H3K4me3**	ERX712686	ERX712722	ERX1007384
**H3K9me3**	ERX712691	ERX712726	ERX1007398

A complete list of the raw files available from the ftp is listed together with associated meta data in the data index file (indexed with secondary sample ERS accession; ftp://ftp.ebi.ac.uk/pub/databases/blueprint/releases/20140811/homo_sapiens/20140811.data.index).

Mapping. Reads were mapped to a gender matched reference (GRCh37) with BWA 0.5.9, with the read trimming parameter (-q) set to 15. The alignments were sorted and duplicates marked with Picard.

Mappings with a MAPQ below 15 were filtered out with samtools (http://ftp.ebi.ac.uk/pub/databases/blueprint/releases/current_release/homo_sapiens).

Segmentation. We segmented the GC B cell genomes with ChIP-seq data for the 6 histone modifications described above using ChromHmm (v1.03) [17]. The input data were the ChIP-Seq bed files with the genomic coordinates and strand orientation of mapped sequences (after removal of duplicate reads). The genome was divided in consecutive 200 bp non-overlapping intervals and independently assigned present (1) or absent (0) for each of the 6 chromatin modifications. The assignment was based on the count of tags mapping to the interval and on the basis of a Poisson background model using a threshold of 10^{-4}, as explained in Ernst et al [17]. After binarization and for segmentation we used the eleven states model established by the Blueprint Consortium (http://ftp.ebi.ac.uk/pub/databases/blueprint/paper_data_sets/monocyte_neutrophil_2014/chromatin_states/full_histone_panel/model_11_Blueprint_11.txt). Further, we computed the probability that each location is in a given chromatin state, and then assigned each 200-bp interval to its most likely state for each sample. Lastly, consecutive intervals within the same chromatin state were joined [18].
Finally, the 11 chromatin states were collapsed into the 5 functional chromatin states: promoter, poised promoter, enhancer, transcription and heterochromatin [19].

GC B cell consensus state. The chromatin state segmentations derived from GC B cells were combined (bedtools v2.25.0) and for each segment the consensus chromatin state was defined based on a majority voting. In case of an ambiguous result the artificial state "0" was assigned.

Identification of hallmarks events
Fluorescence in situ hybridization (FISH) on interphase nuclei was performed on frozen tissue using specific probes for the hallmark events in gcBCL obtained from Abbott Molecular: LSI BCL6, LSI MYC, LSI IGH/MYC, CEP8 Tri-color, LSI IGH and LSI BCL2. Digital image acquisition, processing, and evaluation was performed using ISIS digital image analysis version 5.0 (MetaSystems). At least 100 nuclei were examined by two independent observers for each probe whenever possible. FISH and WGS data were combined to determine a consensus state for the hallmark events. For the detection of aberrations by WGS two independent tools, SOPHIA and DELLY, were applied. The aberrations were classified as positive when FISH and at least one WGS-based caller identified the rearrangement, as discrepant in case FISH results were negative but both WGS-based methods detected a rearrangement or vice versa, and as negative otherwise. Discrepant cases were re-classified as positive if fusion transcripts affecting the hallmark genes could be identified by RNA-Seq data and remained discrepant otherwise (Suppl. Table 1).

Transcriptome analysis
Transcriptome data were mapped with segemehl 0.2.0 [20], allowing for spliced alignments and using a minimum accuracy of 90%. Gene expression values were counted using RNAcounter 1.5.2 (https://pypi.python.org/pypi/rnacounter), using the "--nh" option and counting only exonic reads (-t exon).

Gene expression based gcBCL classification
No reliable classification of ABC- and GCB-DLBCL based on histology or immunohistochemistry is available thus far. Previous studies relied on microarray expression data [21,22]. A set of 23 differentially expressed genes to distinguish between DLBCL of type ABC and GCB has been established previously [23]. We used these genes to classify our cohort based on RNA-Seq data.
For an initial set of 38 lymphomas, classified as DLBCL by histopathological review, RNA-seq data for all 23 gene regions was available on the average reads per million (RPM) scale. Expression values were log transformed and we applied unsupervised hierarchical clustering on both, genes and lymphoma samples.
The clustering of the genes resulted in a perfect separation of genes up- and downregulated in ABC-DLBCL. Based on the hierarchical clustering and the expression pattern of high expression in ABC-DLBCL upregulated genes and low expression in ABC-DLBCL downregulated genes and vice versa we

defined 3 clusters. 13 samples were termed ABC-like, 17 GCB-like and 8 samples in between ABC-like and GCB-like were termed unclassified.

We estimated the density of expression values separately for ABC-like and GCB-like DLBCL samples assuming normal distribution of expression values. For each sample, the observed expression value was compared with estimated distributions and the conditional probability for ABC-DLBCL was estimated based on the observed expression. The evaluation of a sample resulted in 23 estimated ABC-DLBCL probabilities, one for each gene included in the classifier. We applied the median of the estimated ABC-DLBCL probabilities as an overall classification score for ABC- vs GCB-DLBCL. Given the results of the test set with 38 DLBCL we applied the thresholds of <0.25 for GCB-DLBCL and >=0.66 for ABC-DLBCL. Samples in between were labelled as unclassified lymphoma. The classifier was applied to all samples with available RNA-Seq data included in the analysis (n=176).

Extraction of clusters of SNVs at high and intermediate mutation density

At per sample level, SNVs were defined to belong to kataegis clusters [24,25] if at least five consecutive SNVs had a maximum intermutation distance of 1000 bp. Recurrence across the cohort was assessed by investigating pairwise overlaps between kataegis clusters by the functions `coverage()` and `reduce()` from the R-package GenomicRanges [26]. Whenever overlap of kataegis clusters from at least three samples was observed, the minimal region of overlap of these clusters was termed kataegis region.

We introduce the term psichales (ψιχάλες, ancient greek for "drizzling rain") for clusters of SNVs at intermediate mutation density. Identification of psichales clusters in a sample is defined depending on total mutational load of this sample. The minimum number of SNVs required to be present in a psichales cluster, *min_count*, and the maximum intermutation distance, δ, were defined according to the following empirically determined formulae:

$$\delta = minimum\left(400kbp, 3.5 \cdot median(intermut\_dist(SNV\_coords))\right) \quad (2)$$

$$min\_count = maximum(7, 0.0011 \cdot number\_of\_SNVs) \quad (3)$$

where *intermut_dist(SNV_coords)* represents the vector of intermutational distances of the SNVs of a sample. Recurrence across the cohort was evaluated as described above for kataegis. Kataegis and psichales clusters and regions were annotated by aggregating the respective information from all member SNVs (categorical information by tabulation, continuous information by computing mean and standard deviation).

In order to investigate the presence of clusters at high and intermediate mutation density also in other entities, we extracted kataegis and psichales clusters from SNV calls of eight different data sets originating from different entities and different tissues of origin (three breast cancer cohorts, one cohort of chronic lymphocytic leukemia (CLL), one external DLBCL cohort, two cohorts of ovarian cancer and one sarcoma cohort [25,27-29] (the analyses are in part based upon data generated by the TCGA research network: http://cancergenome.nih.gov/)). Data analysis was done with the same methodology as performed in our manuscript for gcBCL.

Determination of IG switch regions

Multiple layers of information were integrated in order to define genomic coordinates for the switch regions in the constant part of the IGH locus. Many V, D, and J genes and the genes IGHA2, IGHE, IGHG4, IGHG2, IGHA1, IGHG1, IGHG3, IGHM and IGHD (called IGHconst genes) inside the IGH locus are annotated in hg19 and thus the overall genomic boundaries of IGH are known. The data layers for each IGHconst gene are depicted in Figs. S6 and S7.

Coordinates of the intergenic regions telomeric of the IGH constant genes were extracted. We call these regions pre-IGHconst (pre-IGHA2, pre-IGHE, ..., pre-IGHM) regions. Further analysis was based on these regions and their flanking genes. We retrieved 106 contigs from IMGT/LIGM-DB [30] (as of March 03, 2017, query parameters SPECIES=homo sapiens (human) and OTHER_KEYWORDS=switch). The names of the contigs were parsed and categorical information was annotated: (a) contig known to originate from one single switch region; (b) contig known to originate from a recombination, i.e. deletion and fusion of the breakpoints. Pairwise alignments of these contigs and the pre-IGHconst regions were performed with the function *pairwiseAlignment()* from the Bioconductor package Biostrings [31]. Contigs

of type (a) and (b) were aligned globally with regards to IGMT contigs and locally with regards to the pre-IGHconst regions (type=”global-local”) and termed oGL (original glocal-local) and jL (junction local), respectively, while contigs of type (a) were additionally aligned with a purely local alignment (type=”local”), termed oL (original local). Furthermore, kataegis regions and repeat regions defined by repeat masker (track *Simple Repeats* from ENSEMBL) overlapping with pre-IGHconst regions were extracted. Strandedness of the repeats was omitted and directly adjacent repeats were merged (visualized in the track *redRep*). Finally, breakpoints of SV events detected in the *merged non-BL/BL* cohort were categorized:

1. Hallmark translocations of IGH: one breakpoint located in IGH and the other breakpoint located in *MYC, BCL2* or *BCL6*.
2. Translocations involving IGH and other genomic loci.
3. Recombination events inside the IGH locus (including CSR).

For visualization, breakpoints of class (1) were furthermore split into 5 groups, reflecting the subgroups of gcBCLs. Events were plotted as separate tracks. Breakpoints of class (2) were plotted as a common track regardless of subgroup attribution. Recombination events (class 3) were plotted as the topmost track.

Consensus switch regions were extracted (highlighted as red rectangles in the subplots of Fig. S6 and S7) as maximal regions of overlap of a subset of the features described above. First, regions from all alignment tracks from IMGT/LIGM-DB (*oGL*, *oL*, *jL*) were merged, allowing a maximum gap of 1 kbp. Then, regions from the *redRep* track were merged to these consensus regions, allowing a maximum gap of 200 bp. Other tracks were used to ensure plausibility of the extracted switch regions (location 5’ of an IGHconst gene, overlap with a kataegis region).

Classification of hypermutation clusters into SHM-like and CSR-like

For all samples in the *merged non-BL/BL cohort*, profiles of SNVs in their triplet contexts (corresponding to mutational catalogs in the Alexandrov et al. [24] nomenclature) were extracted for the switch regions (all merged together) as well as for the VDJ genes and pseudogenes (all merged together) by counting SNVs in their triplet context.

Accordingly, profiles of SNVs in their triplet contexts were extracted for all kataegis clusters. Supervised decomposition of these profiles by non-negative least squares (NNLS) with the two basis profiles mentioned above was computed. Finally, kmeans clustering (*k=3)* was carried out on the NNLS contributions to yield three categories of kataegis clusters: i) SHM-like (high contributions of the VDJ profile); ii) CSR-like (high contributions of the switch region profile); and iii) other (low contributions of both VDJ and switch region profiles).

Hypermutation by proxy

Pairwise co-occurrence of hypermutation in different kataegis regions was investigated by calculating a custom score for the subject-object relationship. First, for all pairs of kataegis regions, the number of samples with hypermutation in both regions was counted. In each given pair, the kataegis region in which more samples were affected was called the current subject, the kataegis region in which less samples were affected was called the current object. Second, the count arguing against the given subject/object relationship, i.e., the number of samples in which only the object was affected by hypermutation and not the subject was subtracted from the number of samples with hypermutation in both regions. In a third step, the scores obtained were normalized by division by the maximum score across all pairwise combinations, and finally negative values replaced by zero. Co-occurrence was defined to be present if this normalized score was greater than zero. Note that this normalized score is not symmetric with regards to exchanging subject and object. As one kataegis region can be subject to several objects and/or object to several subjects, integrated region-wise subject scores (as opposed to the current subject score as defined above) were evaluated as the sum of current subject scores over all possible objects, and integrated region-wise object scores were evaluated as the sum of current object scores over all possible subjects. Kataegis regions outside the IG loci with integrated subject scores ≥ 0.6 or integrated object scores ≥ 0.6 were selected for display in Fig. 3A.

For some subject-object pairs of interest, including DNMT1/S1PR2, co-expression of the involved genes was investigated separately for the different gcBCL subgroups.

In order to test for an enrichment of RNA chimeras between pairs of kataegis regions, a multi-step procedure was implemented. First, all splice-mapped reads where one part mapped into a kataegis region and the flanking 20 kB and the other to a different chromosome were extracted at per-sample level. Kataegis regions which overlapped with IG-loci as well as *BCL6* and *BCL2* were filtered out, as trans-splicing from these loci is frequent and might distort the signal. Using an in-house perl script calling samtools, splice events supported by at least 3 reads were selected. Beyond counting these splice events, we recorded how many of these splice events had a kataegis region on both ends of a spliced read (in trans, cf. above) and computed the fraction of reads connecting kataegis regions.
In order to generate a background distribution, bedtools shuffle was applied to all genes that showed an expression level comparable to the kataegis region genes (log2 expression of 7 or above in edgeR). We performed 100 shufflings and computed the z-scores of the number of interchromosomal trans-splice events as well as the relative number of pairwise inter-kataegis region splicing.
Chromatin conformation in naive B cells, total B cells from peripheral blood (comprising naive and memory B cells) and CLL cells was assessed in the framework of the BLUEPRINT consortium [32,33]. The kataegis regions extracted from our cohort (as described in the manuscript and in the methods part) were intersected with restriction fragments used in the chromatin conformation experiment. The links between the restriction fragments from chromatin conformation were then used to infer co-localization of the kataegis regions. We would like to state that the promoter capture HiC data is limited; two kataegis regions that both do not represent a bait in the analysis will not be found to interact as the analysis limits itself to detect interactions between baits and other ends.

Unsupervised analysis of mutational signatures
An unsupervised analysis of mutational signatures [24] was performed with non-negative matrix factorization (NMF). As detectability of signatures using NMF strongly depends on statistical power, this analysis was performed on the *merged non-BL/BL* cohort. The function `runNmfGpu()` from the software package Bratwurst [34,35] was used for NMF analyses. It provides a wrapper functions for one NMF solver [36] on graphical processing units (GPUs) using the Compute Unified Device Architecture (CUDA) 8 framework. The factorization rank was varied over the range 2 to 15. For every factorization rank, 500 iterations over random initial conditions were performed. For every initial condition, iteration over update equations was performed until convergence was reached, but interrupted if convergence was not reached after 10,000 iterations. The optimal factorization rank was obtained by simultaneously minimizing the Frobenius error, maximizing the cophenetic correlation coefficient and minimizing the Amari distance. Relationships between signatures extracted at different factorization ranks *k* were be visualized as a Sankey diagrams or riverplot, in which nodes are signatures and edge weights are encoded by coefficients of NNLS decompositions of signatures at factorization rank i by those at rank i-1.

Supervised analysis of mutational signatures
The supervised analysis of mutational signatures was performed with the R package YAPSA [37,38]. The function `LCD_complex_cutoff()` in YAPSA computes an NNLS decomposition of the mutational catalogue with known signatures (L1, L2, and L3 resulting from NMF analysis and 30 signatures from COSMIC (http://cancer.sanger.ac.uk/cosmic/signatures)). In order to increase specificity, `LCD_complex_cutoff()` applies the NNLS algorithm twice. A first NNLS is run proposing all supplied signatures to the decomposition, then a second NNLS is run again with a reduced set of signatures consisting only of those signatures whose exposures, i.e. contributions in the linear combination in the first decomposition, were higher than a certain signature-specific cutoff. The signature-specific cutoffs were determined in a random operator characteristic (ROC) analysis using publicly available data on mutational catalogues of 7,042 cancer samples (507 from whole genome sequencing and 6,535 from whole exome sequencing) [24] and mutational signatures (http://cancer.sanger.ac.uk/cosmic/signatures, downloaded on January 15th, 2016). The following cut-offs were employed - AC1: 0; AC2: 0.01045942; AC3: 0.08194056; AC4: 0.01753969; AC5: 0; AC6: 0.001548535; AC7: 0.04013304; AC8: 0.242755; AC9: 0.1151714; AC10: 0.01008376; AC11: 0.09924884; AC12: 0.2106201; AC13: 0.007876626; AC14: 0.1443059; AC15: 0.03796027; AC16:

0.3674349; AC17: 0.002647962; AC18: 0.3325386; AC19: 0.1167454; AC20: 0.1235028; AC21: 0.1640255; AC22: 0.03102216; AC23: 0.03338659; AC24: 0.03240176; AC25: 0.01611908; AC26: 0.09335221; AC27: 0.009320062; AC28: 0.05616434; AC29: 0.05936213; AC30: 0.05915355; L1: 0; L2: 0; L3: 0.

Synthetic SHM signature

Yaari et al [39] have extracted synonymous mutations from V and J genes of the IGH locus from normal B cells in their 5-mer sequence context to obtain the fingerprint of physiologic SHM. They provide separate files encoding the mutation probability per motif (http://clip.med.yale.edu/shm/distribution/Mutability.csv) and the probabilities for the different nucleotide exchanges (http://clip.med.yale.edu/shm/distribution/Substitution.csv). Multiplication of corresponding values from these two tables yields the probability for a given nucleotide exchange in its 5-mer context. We aggregated this information into a format comparable to mutational signatures of SNVs in their triplet context and termed the resulting pattern synthetic SHM signature.

Stratified analyses of mutational signatures

In order to identify enrichment and depletion patterns, YAPSA was used to stratify the analysis of mutational signatures. Stratification was performed along different stratification axes. Using the function `run_SMC()` from YAPSA, the stratified analysis was performed as a multistep procedure [40]: (1) a supervised analysis of mutational signatures was run without any stratification; (2) for every SNV in a sample, the stratum it belonged to was annotated; (3) for every stratum, a stratum-specific mutational catalog was built; and (4) a supervised NNLS (using lsei) with the constraint that the sum of exposures per stratum equals the exposures computed by the unstratified analysis was performed. Thereafter, enrichment and depletion patterns for all mutational signatures detected in step 1 were computed from the exposures in all strata with the help of the function `stat_test_SMC()`. Kruskal Wallis tests were used as discovery tests and corrected for multiple testing according to Benjamini and Hochberg (BH); only if the discovery test after correction for multiple testing was significant, pairwise posthoc Nemenyi tests were performed.

Detection of oncodrivers

IntOGen [41] (version 3.0.5) was run with default settings on all SNVs and indels detected in samples with matched control to identify driver genes. Additionally, IntOGen was run separately on all FL and all DLBCL cases with matched control. The union of the full cohort as well as the FL- and DLBCL-specific gene sets was used as driver gene set.

Integration of different variant types

SNVs, indels, SVs and CNAs were integrated in order to account for all variant types in the recurrence analysis. Whilst all genes with SNVs or indels in coding regions (nonsynonymous, splicing, frameshift event) and ncRNA were included, SVs and CNAs were handled differently. Any genes between the breakpoints of focal SVs (<1 Mbp) were considered affected. However, duplications and deletions in the range of 10 kbp to 1 Mbp had to be verified by ACEseq, and subclonal events with a deviation of less than 0.7 copy numbers from the average ploidy were discarded. For larger SVs only genes that were directly hit by a breakpoint were considered. Only focal CNA events (<1 Mbp) were taken into account for variant integration, as these are more likely to target specific genes within the affected region than large events such as whole chromosome arm events. To capture the precise target, focal SVs and CNAs were combined and the minimal regions of interest (ROIs), i.e. local maxima of overlapping regions with more than one event, were identified. Finally, genes affected by SNVs, indels, focal and large SVs or genes within the CNA ROIs were considered for the recurrence analysis. Any gene affected ≥19 times was further looked up in focal CNA regions outside of ROIs and added to the oncoprints. Variants in ncRNAs were only considered for cases with matched control.

AID enrichment and signature analysis of driver and recurrently mutated genes

The CSR profile consists of C>T SNVs in DGC/GCH motifs and C>G SNVs in GCT motifs. In order to test enrichments, occurrences of these specific base exchanges in the motifs were counted along with

other base exchanges within and any base exchanges outside of these motifs and used for computation of odds ratios. This was done for all recurrently mutated genes (≥19 samples affected) and recurrently mutated driver genes (≥11 samples affected).

Additionally, we determined the most likely signature per gene. To this end, we compiled a catalogue of cohort-wide SNVs for each gene in their triplet context. These were compared to the mutational signatures extracted for our cohort by cosine similarity, and the most similar mutational signature was annotated to the respective gene. The cosine similarity of two vectors is defined as the cosine of the angle between these two vectors, yielding a maximum similarity of 1 for parallel vectors and 0 for orthogonal vectors; equivalent to Pearson correlation.

NMF Consensus Clustering

We binarized the different classes of mutations (SNVs, small Indels, SVs and CNAs) identified in driver genes and recurrent genes and ran an NMF analysis. As SVs and CNAs cannot be attributed to genes unambiguously, some neighbouring genes which were recurrently coaffected by the same event were grouped: a BRD2_cluster containing *BRD2*, *XXbac-BPG181M17.5*, *BRD2-IT1*, *HLA-DMA*, *XXbac-BPG181M17.6* and *HLA-DMB*; an S1PR2_cluster containing *S1PR2* and *DNMT1*; a MEF2B_cluster containing *MEF2B*, *MEF2BNB-MEF2B*; a CDKN2_cluster containing *CDKN2B-AS1*, *CDKN2B*, *RP11-149I2.4*, *RP11-145E5.5*, *CDKN2A*, *MTAP*, *RP11-70L8.4* and *C9orf53*; a CIITA_cluster containing *CIITA* and *RP11-876N24.2*; and finally a BCL6_cluster containing *RP11-211G3.2* and *BCL6*. In order to rule out false positive findings, the four hypermutated samples 4109808, 4145528, 4199714 and 4163639 were excluded from this analysis.

In a first step, this analysis was restricted to the 76 non-hypermutated DLBCL cases of our gcBCL cohort. Recurrence thresholds for genes to enter this analysis were 12 in our whole gcBCL cohort and 6 in the non-hypermutated DLBCL cohort. The optimal factorization rank was determined as described above in the section “Unsupervised analysis of mutational signatures”. If these criteria did not yield an unambiguous optimal factorization rank, the coefficient of variation and the average silhouette width were used as additional quality criteria. Clusters were labelled by the most informative representative of the recurrently affected genes inside the cluster

In a second step we also analyzed the merged cohort of different gcBCL entities (other than BL). Recurrence thresholds for genes to enter this analysis were 12 across our whole gcBCL cohort and 10 in the remaining non-hypermutated cohort. The optimal factorization rank was determined as described above for the DLBCL subcohort. Again, clusters were labelled by the most informative representative of the recurrently affected genes inside the cluster.

Data Availability

Sequencing data has been deposited in the European Genome-Phenome Archive (EGA) under accession number EGAS00001002199. WGS data for samples that were part of the ICGC PCAWG are available under https://www.ebi.ac.uk/ega/studies/EGAS00001001692. A list of samples available from the PCAWG repository can be found under http://pancancer.info/gnos_metadata/latest/reports/donors_alignment_summary/MALY-DE.both_aligned.donors.txt.

Code Availability

All code used within the manuscript is available from the corresponding authors upon reasonable request.

Plotting conventions

All boxplots in this work use the following standard convention of the function `geom_boxplot()` from the R package ggplot2: The upper and lower ends of the box correspond to the first and third quartiles (the 25th and 75th percentiles). The line in between represents the median of the distribution. The upper whisker extends from the hinge to the highest value that is within 1.5 * IQR of the hinge, where IQR is the inter-quartile range, or distance between the first and third quartiles. The lower whisker extends from the hinge to the lowest value within 1.5 * IQR of the hinge. Data beyond the end of the whiskers are outliers and plotted as points.

In several figures, boxplots with the conventions described above and violin plots are overlayed.

References

1. López C, Kleinheinz K, Aukema SM, Rohde M, Bernhart SH, Hübschmann D*, et al.* Genomic and transcriptomic changes complement each other in the pathogenesis of sporadic Burkitt lymphoma. Nat Commun 2019; 10**:** 1459-1459.
2. Richter J, Schlesner M, Hoffmann S, Kreuz M, Leich E, Burkhardt B*, et al.* Recurrent mutation of the ID3 gene in Burkitt lymphoma identified by integrated genome, exome and transcriptome sequencing. Nat Genetics 2012; 44**:** 1316-1320.
3. Swerdlow SH, Campo E, Harris NL, Jaffe ES, Pileri SA, Stein H*, et al. WHO Classification of Tumours of Haematopoietic and Lymphoid Tissues*, vol. 4th. IARC press, 2008, 326-326pp.
4. Kretzmer H, Bernhart SH, Wang W, Haake A, Weniger MA, Bergmann AK*, et al.* DNA methylome analysis in Burkitt and follicular lymphomas identifies differentially methylated regions linked to somatic mutation and transcriptional control. Nat Genetics 2015; 47**:** 1316-1325.
5. Li H, Durbin R. Fast and accurate short read alignment with Burrows-Wheeler transform. Bioinformatics 2009; 25**:** 1754-1760.
6. Jones DTW, Hutter B, Jäger N, Korshunov A, Kool M, Warnatz H-J*, et al.* Recurrent somatic alterations of FGFR1 and NTRK2 in pilocytic astrocytoma. Nat Genetics 2013; 45**:** 927-932.
7. Li H, Handsaker B, Wysoker A, Fennell T, Ruan J, Homer N*, et al.* The Sequence Alignment/Map format and SAMtools. Bioinformatics 2009; 25**:** 2078-2079.
8. Rimmer A, Phan H, Mathieson I, Iqbal Z, Twigg SRF, Wilkie AOM*, et al.* Integrating mapping-, assembly- and haplotype-based approaches for calling variants in clinical sequencing applications. Nat Genetics 2014; 46**:** 1-9.
9. Jabs J, Zickgraf FM, Park J, Wagner S, Jiang X, Jechow K*, et al.* Screening drug effects in patient-derived cancer cells links organoid responses to genome alterations. Mol Sys Biol 2017; 13**:** 955-955.
10. Wang K, Li M, Hakonarson H. ANNOVAR: Functional annotation of genetic variants from high-throughput sequencing data. Nucl Acids Res 2010; 38**:** 1-7.
11. Sherry ST, Ward MH, Kholodov M, Baker J, Phan L, Smigielski EM*, et al.* dbSNP: the NCBI database of genetic variation. Nucl Acids Res 2001; 29**:** 308-311.
12. Sahm F, Toprak UH, Hübschmann D, Kleinheinz K, Buchhalter I, Sill M*, et al.* Meningiomas induced by low-dose radiation carry structural variants of NF2 and a distinct mutational signature. Acta Neuropathol 2017; 134**:** 155-158.
13. Rausch T, Zichner T, Schlattl A, Stütz AM, Benes V, Korbel JO. DELLY: structural variant discovery by integrated paired-end and split-read analysis. Bioinformatics 2012; 28**:** i333-i339.
14. Northcott PA, Buchhalter I, Morrissy AS, Hovestadt V, Weischenfeldt J, Ehrenberger T*, et al.* The whole-genome landscape of medulloblastoma subtypes. Nature 2017; 547**:** 311-317.
15. Kleinheinz K, Bludau I, Huebschmann D, Heinold M, Kensche P, Gu Z*, et al.* ACEseq - allele specific copy number estimation from whole genome sequencing. bioRxiv 2017.
16. Hansen RS, Thomas S, Sandstrom R, Canfield TK, Thurman RE, Weaver M*, et al.* Sequencing newly replicated DNA reveals widespread plasticity in human replication timing. Proc Natl Acad Sci USA 2010; 107**:** 139-144.
17. Ernst J, Kellis M. ChromHMM: automating chromatin-state discovery and characterization. 2012. p. 215-216.
18. Carrillo-de-Santa-Pau E, Juan D, Pancaldi V, Were F, Martin-Subero I, Rico D*, et al.* Automatic identification of informative regions with epigenomic changes associated to hematopoiesis. Nucl Acids Res 2017; 45**:** 9244-9259.
19. Carrillo de Santa Pau E, Juan D, Pancaldi V, Were F, Martin-Subero I, Rico D*, et al.* Searching for the chromatin determinants of human hematopoiesis. bioRxiv 2016.
20. Hoffmann S, Otto C, Kurtz S, Sharma CM, Khaitovich P, Vogel J*, et al.* Fast mapping of short sequences with mismatches, insertions and deletions using index structures. PLoS Comput Biol 2009; 5**:** e1000502-e1000502.
21. Hummel M, Bentink S, Berger H, Klapper W, Wessendorf S, Barth TFE*, et al.* A biologic definition of Burkitt's lymphoma from transcriptional and genomic profiling. N Engl J Med 2006; 354**:** 2419-2430.
22. Wright G, Tan B, Rosenwald A, Hurt EH, Wiestner A, Staudt LM. A gene expression-based method to diagnose clinically distinct subgroups of diffuse large B cell lymphoma. Proc Natl Acad Sci USA 2003; 100**:** 9991-9996.
23. Care MA, Barrans S, Worrillow L, Jack A, Westhead DR, Tooze RM. A microarray platform-independent classification tool for cell of origin class allows comparative analysis of gene expression in diffuse large B-cell lymphoma. PLos One 2013; 8**:** e55895-e55895.
24. Alexandrov LB, Nik-Zainal S, Wedge DC, Aparicio SaJR, Behjati S, Biankin AV*, et al.* Signatures of mutational processes in human cancer. Nature 2013; 500**:** 415-421.
25. Nik-Zainal S, Alexandrov LB, Wedge DC, Van Loo P, Greenman CD, Raine K*, et al.* Mutational processes molding the genomes of 21 breast cancers. Cell 2012; 149**:** 979-993.
26. Lawrence M, Huber W, Pagès H, Aboyoun P, Carlson M, Gentleman R*, et al.* Software for computing and annotating genomic ranges. PLoS Comput Biol 2013; 9**:** e1003118-e1003118.
27. Nik-Zainal S, Davies H, Staaf J, Ramakrishna M, Glodzik D, Zou X*, et al.* Landscape of somatic mutations in 560 breast cancer whole-genome sequences. Nature 2016; 534**:** 47-54.
28. Patch A-M, Christie EL, Etemadmoghadam D, Garsed DW, George J, Fereday S*, et al.* Whole–genome characterization of chemoresistant ovarian cancer. Nature 2015; 521**:** 489-494.
29. Puente XS, Beà S, Valdés-Mas R, Villamor N, Gutiérrez-Abril J, Martín-Subero JI*, et al.* Non-coding recurrent mutations in chronic lymphocytic leukaemia. Nature 2015; 526**:** 519-524.
30. Giudicelli V, Duroux P, Ginestoux C, Folch G, Jabado-Michaloud J, Choume D*, et al.* IMGT/LIGM-DB, the IMGT(R) comprehensive database of immunoglobulin and T cell receptor nucleotide sequences. Nucl Acids Res 2006; 34**:** D781-D784.
31. Pagès H, Aboyoun P, Gentleman R, DebRoy S. Biostrings: String objects representing biological sequences, and matching algorithms. Bioconductor; 2016.
32. Beekman R, Chapaprieta V, Russiñol N, Vilarrasa-Blasi R, Verdaguer-Dot N, Martens JHA*, et al.* The reference epigenome and regulatory chromatin landscape of chronic lymphocytic leukemia. Nat Med 2018; 24**:** 868-880.
33. Javierre BM, Burren OS, Wilder SP, Kreuzhuber R, Hill SM, Sewitz S*, et al.* Lineage-specific genome architecture links enhancers and non-coding disease variants to target gene promoters. Cell 2016; 167**:** 1369-1384.e1319.
34. Huebschmann D, Kurzawa N, Steinhauser S, Rentzsch P, Kraemer S, Andresen C*, et al.* Deciphering programs of transcriptional regulation by combined deconvolution of multiple omics layers. bioRxiv 2017**:** 199547-199547.
35. Quintero A, Hubschmann D, Kurzawa N, Steinhauser S, Rentzsch P, Kramer S*, et al.* ShinyButchR: Interactive NMF-based decomposition workflow of genome-scale datasets. Biol Methods Protoc 2020; 5**:** bpaa022.

36. Mejía-Roa E, Tabas-Madrid D, Setoain J, García C, Tirado F, Pascual-Montano A. NMF-mGPU: non-negative matrix factorization on multi-GPU systems. BMC Bioinf 2015; 16**:** 43-43.
37. Huebschmann D, Gu Z, Schlesner M. YAPSA: Yet Another Package for Signature Analysis. Bioconductor; 2015.
38. Hübschmann D, Jopp-Saile L, Andresen C, Kramer S, Gu Z, Heilig CE*, et al.* Analysis of mutational signatures with yet another package for signature analysis. Genes Chromosomes Cancer 2020; early online, Nov. 22.
39. Yaari G, Vander Heiden JA, Uduman M, Gadala-Maria D, Gupta N, Joel JN*, et al.* Models of somatic hypermutation targeting and substitution based on synonymous mutations from high-throughput immunoglobulin sequencing data. Front Immunol 2013; 4**:** 1-6.
40. Giessler KM, Kleinheinz K, Huebschmann D, Balasubramanian GP, Dubash TD, Dieter SM*, et al.* Genetic subclone architecture of tumor clone-initiating cells in colorectal cancer. J Exp Med 2017; 214**:** 2073-2088.
41. Gonzalez-Perez A, Perez-Llamas C, Deu-Pons J, Tamborero D, Schroeder MP, Jene-Sanz A*, et al.* IntOGen-mutations identifies cancer drivers across tumor types. Nat Genetics 2013; 10**:** 1081-1084.
