# Supplementary material for: Mutational mechanisms shaping the coding and noncoding genome of germinal center derived B-cell lymphomas
Source: Leukemia. 2021 May 5;35(7):2002–16. doi: 10.1038/s41375-021-01251-z (PMC8257491; doi:10.1038/s41375-021-01251-z)
Supplement: Supplementary file 2 — Supplementary Information [file 41375_2021_1251_MOESM2_ESM.pdf]

Supplement to “Mutational mechanisms shaping the coding and non-coding genome of germinal center-derived B-cell lymphomas”

Supplementary Information

List of members

Full list of members of the ICGC MMML-seq

Coordination (C1): Reiner Siebert[1,2], Susanne Wagner[2], Andrea Haake[2], Julia Richter[2,3], Gesine Richter[2]
Data Center (C2): Roland Eils[4,5], Chris Lawerenz[4], Jürgen Eils[4], Jules Kerssemakers[4], Christina Jaeger-Schmidt[4], Ingrid Scholz[4]
Clinical Centers (WP1): Anke K. Bergmann[2, 6], Christoph Borst[7], Birgit Burkhardt[8], Alexander Claviez[6], Martin Dreyling[9], Sonja Eberth[10], Hermann Einsele[11], Norbert Frickhofen[12], Siegfried Haas[7], Martin-Leo Hansmann[13], Dennis Karsch[14], Michael Kneba[14], Jasmin Lisfeld[15], Luisa Mantovani-Löffler[16], Marius Rohde[15], German Ott[17], Christina Stadler[10], Peter Staib[18], Stephan Stilgenbauer[19], Lorenz Trümper[10], Thorsten Zenz[20]
Normal Cells (WPN): Martin-Leo Hansmann[13], Dieter Kube[10], Ralf Küppers[21], Marc Weniger[21]
Pathology and Analyte Preparation (WP2-3): Michael Hummel[22], Wolfram Klapper[3], Ulrike Kostezka[23], Dido Lenze[22], Peter Möller[24], Andreas Rosenwald[25], German Ott[17], Monika Szczepanowski[3]
Sequencing and genomics (WP4-7): Ole Ammerpohl[1,2], Sietse M. Aukema[2,3], Vera Binder[26], Arndt Borkhardt[26], Andrea Haake[2], Jessica I. Hoell[26]; Ellen Leich[25], Peter Lichter[27], Cristina López[1,2], Inga Nagel[2], Jordan Pischimariov[25], Bernhard Radlwimmer[27], Julia Richter[2,3], Philip Rosenstiel[28], Andreas Rosenwald[25], Markus Schilhabel[28], Stefan Schreiber[29], Inga Vater[2], Rabea Wagener[1,2], Reiner Siebert[1,2]
Bioinformatics (WP8-9): Stephan H. Bernhart[30-32,] Hans Binder[30,31], Benedikt Brors[33], Gero Doose[30-32], Roland Eils[4,5], Steve Hoffmann[30-32], Lydia Hopp[30], Daniel Hübschmann[4,5,34], Kortine Kleinheinz[4,5], Helene Kretzmer[30-32], Markus Kreuz[35], Jan Korbel[36], David Langenberger[30-32], Markus Loeffler[35], Maciej Rosolowski[35], Matthias Schlesner[4,37], Peter F. Stadler[31], Stephanie Sungalee[36]

[1]Institute of Human Genetics, University of Ulm and University Hospital of Ulm, Ulm, Germany; [2]Institute of Human Genetics, Christian-Albrechts-University, Kiel, Germany;
[3]Hematopathology Section, Institute of Pathology, Christian-Albrechts-University, Kiel, Germany; [4]Division of Theoretical Bioinformatics (B080), German Cancer Research Center (DKFZ), Heidelberg, Germany;
[5]Department for Bioinformatics and Functional Genomics, Institute of Pharmacy and Molecular Biotechnology and Bioquant, University of Heidelberg, Heidelberg, Germany;
[6]Department of Pediatrics, University Hospital Schleswig-Holstein, Campus Kiel, Kiel, Germany; [7]Department of Internal Medicine/Hematology, Friedrich-Ebert-Hospital, Neumünster;
[8]University Hospital Muenster - Pediatric Hematology and Oncology, Muenster Germany;
[9]Department of Medicine III - Campus Grosshadern, University Hospital Munich, Munich, Germany;
[10]Department of Hematology and Oncology, Georg-August-University of Göttingen, Göttingen, Germany;
[11]University Hospital Würzburg, Department of Medicine and Poliklinik II, University of Würzburg, Würzburg;
[12]Department of Medicine III, Hematology and Oncology, Dr. Horst-Schmidt-Kliniken of Wiesbaden, Wiesbaden;
[13]Senckenberg Institute of Pathology, University of Frankfurt Medical School, Frankfurt am Main, Germany;
[14]Department of Internal Medicine II: Hematology and Oncology, University Medical Centre, Campus Kiel, Kiel;
[15]University Hospital Giessen, Pediatric Hematology and Oncology, Giessen, Germany;
[16]Hospital of Internal Medicine II, Hematology and Oncology, St-Georg Hospital Leipzig, Leipzig, Germany;
[17]Department of Pathology, Robert-Bosch-Hospital, Stuttgart, Germany;
[18]Clinic for Hematology and Oncology, St.-Antonius-Hospital, Eschweiler;
[19]Department for Internal Medicine III, University of Ulm and University Hospital of Ulm, Ulm, Germany
[20]National Centre for Tumor Disease, Heidelberg, Germany;
[21]Institute of Cell Biology (Cancer Research), University of Duisburg-Essen, Duisburg-Essen, Medical School, Essen, Germany;
[22]Institute of Pathology, Charité – University Medicine Berlin, Berlin, Germany;
[23]Comprehensive Cancer Center Ulm (CCCU), University Hospital Ulm, Ulm, Germany;
[24]Institute of Pathology, University of Ulm and University Hospital of Ulm, Ulm;

[25]Institute of Pathology, University of Wurzburg, Germany;
[26]Department of Pediatric Oncology, Hematology and Clinical Immunology, Heinrich-Heine-University, Düsseldorf, Germany;
[27]German Cancer Research Center (DKFZ), Division of Molecular Genetics, Heidelberg, 69120, Germany;
[28]Institute of Clinical Molecular Biology, Christian-Albrechts-University, Kiel, Germany;
[29]Department of General Internal Medicine, University Kiel, Kiel, Germany;
[30]Interdisciplinary Center for Bioinformatics, University of Leipzig, Leipzig, Germany;
[31]Bioinformatics Group, Department of Computer, University of Leipzig, Leipzig, Germany; [32]Transcriptome Bioinformatics, LIFE Research Center for Civilization Diseases, University of Leipzig, Leipzig, Germany;
[33]Division of Applied Bioinformatics (G200), German Cancer Research Center (DKFZ), Heidelberg, Germany;
[34]Department of Pediatric Immunology, Hematology and Oncology, University Hospital, Heidelberg, Germany;
[35]Institute for Medical Informatics Statistics and Epidemiology, University of Leipzig, Leipzig, Germany;
[36]EMBL Heidelberg, Genome Biology, Heidelberg, Germany;
[37]Bioinformatics and Omics Data Analytics (B240), German Cancer Research Center (DKFZ), Heidelberg, Germany

Full list of members of the MMML

Pathology group and analytes preparation: Thomas F.E. Barth[1], Heinz-Wolfram Bernd[2], Sergio B. Cogliatti[3], Alfred C. Feller[2], Martin L. Hansmann[4], Michael Hummel[5], Wolfram Klapper[6], Dido Lenze[5], Peter Möller[1], Hans-Konrad Müller-Hermelink[7], German Ott[7], Andreas Rosenwald[7], Harald Stein[5], Monika Szczepanowski[6], Hans-Heinrich Wacker[6].

Genetics group: Thomas F.E. Barth[1], Petra Behrmann[8], Peter Daniel[10], Judith Dierlammm[8], Eugenia Haralambieva[7], Lana Harder[11], Paul-Martin Holterhus[12], Ralf Küppers[13], Dieter Kube[13], Peter Lichter[14], Jose I. Martín-Subero[11], Peter Möller[1], Eva M. Murga-Peñas[9], German Ott[7], Christiane Pott[16], Armin Pscherer[15], Andreas Rosenwald[7], Carsten Schwaenen[17], Reiner Siebert[18], Heiko Trautmann[16], Martina Vockerodt[19], Swen Wessendorf[16].

Bioinformatics group: Stefan Bentink[20], Hilmar Berger[21], Dirk Hasenclever[21], Markus Kreuz[21], Markus Loeffler[21], Maciej Rosolowski[21], Rainer Spang[20].

Project coordination: Benjamin Stürzenhofecker[14], Lorenz Trümper[14], Maren Wehner[14].

Steering committee: Markus Loeffler[21], Reiner Siebert[18], Harald Stein[5], Lorenz Trümper[14].

[1]Institute of Pathology, University Hospital of Ulm, Ulm, Germany; [2]Institute of Pathology, University Hospital Schleswig-Holstein Campus Lübeck, Lübeck, Germany; [3]Institute of Pathology, Kantonsspital St. Gallen, St.Gallen, Switzerland; [4]Institute of Pathology, University Hospital of Frankfurt, Frankfurt, Germany; [5]Institute of Pathology, Campus Benjamin Franklin, Charité–Universitätsmedizin Berlin, Berlin, Germany; [6]Institute of Hematopathology, University Hospital Schleswig-Holstein Campus Kiel/ Christian-Albrechts University Kiel, Kiel, Germany; [7]Institute of Pathology, University of Würzburg, Würzburg, Germany; [8]Cytogenetic and Molecular Diagnostics, Internal Medicine III, University Hospital of Ulm, Ulm, Germany; [9]University Medical Center Hamburg-Eppendorf, Hamburg, Germany; [10]Department of Hematology, Oncology and Tumor Immunology, University Medical Center Charité, Berlin, Germany; [11]Institute of Human Genetics, University Hospital Schleswig-Holstein Campus Kiel/Christian-Albrechts University Kiel, Kiel, Germany; [12]Division of Pediatric Endocrinology and Diabetes, Department of Pediatrics, University Hospital Schleswig- Holstein Campus Kiel / Christian-Albrechts University Kiel, Kiel, Germany; [13]Institute for Cell Biology (Tumor Research), University of Duisburg-Essen, Essen, Germany; [14]Department of Hematology and Oncology, Georg-August University of Göttingen, Göttingen, Germany; [15]German Cancer Research Center (DKFZ), Heidelberg, Germany; [16]Second Medical Department, University Hospital Schleswig-Holstein Campus Kiel/ Christian-Albrechts University Kiel, Kiel, Germany; [17]Cytogenetic and Molecular Diagnostics, Internal Medicine III, University Hospital of Ulm, Ulm, Germany; [18]Institute of Human Genetics, University of Ulm and University Hospital of Ulm, Ulm, Germany; [19]Department of Pediatrics I, Georg-August University of Göttingen, Göttingen, Germany; [20]Institute of Functional Genomics, University of Regensburg, Regensburg, Germany; [21]Institute for Medical Informatics, Statistics and Epidemiology, University of Leipzig, Leipzig, Germany.

List of principal investigators and involved members of the BLUEPRINT project

Project coordinator: Hendrik Stunnenberg[1]
Standardization and Quality control (WP1-2): Xavier Estivill[2], Ivo Gut[3], Hélène Pendeville[4], Joost H.A. Martens[1], Hendrik Stunnenberg[1]
Epigenome of haematopoietic cells (WP3): Frank Grosveld[5], Willem Ouwehand[6], Joost Martens[1], Hendrik Stunnenberg[1]
Epigenome of normal and neoplastic B- and T-cells (WP4): Elias Campo[7], Jude Fitzgibbon[8], Ralf

Küppers[9], Markus Loeffler[10], Elizabeth Macintyre[11], Jose Ignacio Martin Subero[7], Marta Kulis[7], Reiner Siebert[12], Salvatore Spicuglia[13], Hendrik Stunnenberg[1]
Epigenome of acute myeloid leukaemia (WP5): Tariq Enver[14], Joost Martens[1], Hendrik Stunnenberg[1], Edo Vellenga[15]
Data coordination and analysis (WP6-7): Paul Flicek[16], Roderic Guigo[17], Ivo Gut[3], Markus Loeffler[10], Joost H.A. Martens[1], Martin Seifert[18], Amos Tanay[19], David Torrents[20], Alfonso Valencia[21], Martin Vingron[22]
DNA methylation variation in T1DM (WP8): Stephan Beck[23], Bernhard Boehm[24], Åke Lernmark[25], David Leslie[26], Vardham Rakyan[26]
Biomarker development (WP9): Christoph Bock[27], Manel Esteller Badosa[28], Thomas Lengauer[29], Edo Vellenga[15]
The effect of common sequence variation on the epigenome landscape (WP10): Stylianos E. Antonarakis[30], Manolis Dermitzakis[30], Hans Lehrach[31], Willem Ouwehand[6] Nicole Soranzo[32], Hendrik Stunnenberg[1]
Mouse models to quantify variation in reference epigenomes (WP11): David Adams[32], Anne Ferguson-Smith[33], Salvatore Spicuglia[13]
Technology development for genome wide and selected profiling of cytosine (hydroxy)methylation (WP12): Adrian Bird[34], Wolf Reik[35], Dirk Schübeler[36], Michael Stratton[32]
Technology optimization for microscale application (WP13): Hélène Pendeville[4], Hendrik Stunnenberg[1], Eileen Furlong[37], Christoph Merten[37]
Identification/validation of Epi-targets and compound development and screening (WP14- 15): Lucia Altucci[38], Gerard Drewes[39], Laura Maccari[40], Thomas Graf[41], Kristian Helin[42], Antonello Mai[43], Saverio Minucci[44], Pier Giuseppe Pelicci[45]
Training and dissemination (WP16, 17 and 19): Claudia Schacht[46], Martin Seifert[18], Hendrik Stunnenberg[1], Jörn Walter[47]
ChIP-Seq of cell lines: Anke K. Bergmann[12,47], Hindrik. H.D. Kerstens[1], Laura Clarke[16], David Richardson[16], Enrique Carrillo-de Santa Pau[21], Daniel Rico[21]

[1]Radboud University, Department of Molecular Biology, Nijmegen, Netherlands; [2]Centre for Genomic Regulation, Genes and Diseases Program, Barcelona, Spain; [3]Centro Nacional de Analisis Genomico, Barcelona, Spain;
[4]Diagenode SA, Epigenetic Laboratory, Liege, Belgium;
[5]Erasmus University Medical Centre Rotterdam, Department of Cell Biology, Rotterdam, Netherlands;
[6]University of Cambridge, Department of Haematology, Cambridge, United Kingdom;
[7]Institut D'investigacions Biomediques August Pi I Sunyer, Center for Biomedical Diagnosis, Barcelona, Spain;
[8]Queen Mary University of London, Institute of Cancer, London, United Kingdom;
[9]University Hospital Essen, Institute of Cell Biology (Cancer Research), Essen, Germany;
[10]University of Leipzig, Institute for Medical Informatics, Statistics and Epidemiologie (IMISE), Leipzig, Germany;
[11]Centre National de la Recherche Scientifique, UMR-814 and Paris Descartes Université, Laboratoire d'hématologie, CNRS 8147, Paris, France;
[12]Institute of Human Genetics, University Hospital Schleswig-Holstein Campus Kiel/Christian-Albrechts University Kiel, Kiel, Germany;
[13]Institut National de la Sante et de la Recherche Medicale, TAGC Laboratory, Inserm U1090, Marseille, France;
[14]University College London, Department of Cancer Biology, London, United Kingdom;
[15]University Medical Centre Groningen, Department of Hematology, Groningen, Netherlands;
[16]European Bioinformatics Institute, Hinxton, United Kingdom;
[17]Centre for Genomic Regulation, Bioinformatics and Genomics Program, Barcelona, Spain
[18]Genomatix Software GmbH, Munich, Germany;
[19]Weizmann Institute of Science, Department of Science and Applied Mathematics, Rehovot, Israel;
[20]Barcelona Supercomputing Center, Department of Life Sciences - Computational Genomics, Barcelona, Spain;
[21]Structural Biology and Bio Computing Programme, Spanish National Cancer Research Center (CNIO), Madrid, Spain;
[22]Max Planck Institute for Molecular Genetics, Department of Computational Molecular Biology, Berlin, Germany;
[23]University College London, Cancer Institute, London, United Kingdom;
[24]University of Ulm, Department of Internal Medicine, Ulm, Germany;
[25]Lund University, Department of Clinical Sciences Malmö, Malmö, Sweden;
[26]Queen Mary University of London, Blizard Institute of Cell and Molecular Sciences, London, United Kingdom;
[27]CeMM Research Center for Molecular Medicine, Vienna, Austria;
[28]Institut d'Investigacio Biomedica de Bellvitge, Department of Cancer Epigenetics and Biology, Barcelona, Spain;
[29]Max Planck Institute for Informatics, Department of Cancer Epigenetics and Biology, Saarbrücken, Germany;

[30]University of Geneva, Department of Genetic Medicine and Development, Geneva, Switzerland;
[31]Max Planck Institute for Molecular Genetics, Department of Vertebrate Genomics, Berlin, Germany;
[32]Wellcome Trust Sanger Institute, Hinxton, United Kingdom;
[33]University of Cambridge, Department of Genetics, Cambridge, United Kingdom;
[34]University of Edinburgh, Wellcome Trust Centre for Cell Biology, Edinburgh, United Kingdom; [35]The Babraham Institute, Cambridge, United Kingdom;
[36]Friedrich Miescher Institute for Biomedical Research, Basel, Switzerland;
[37]European Molecular Biology Laboratory, Heidelberg, Germany
[38]Second University of Naples, Department of Pathology, Napoli, Italy;
[39]Cellzome AG, Heidelberg, Germany;
[40]Siena Biotech SPA, Siena, Italy;
[41]Centre for Genomic Regulation, Differentiation and Cancer Program, Barcelona, Spain; [42]University of Copenhagen, Biotech Research and Innovation Centre, Copenhagen, Denmark; [43]Sapienza University of Rome, Department of Drug Chemistry and Technologies, Rome, Italy; [44]European Institute of Oncology, Department of Experimental Oncology, Milan, Italy; [45]European Research and Project Office GmbH, Saarbrücken, Germany;
[46]University of Saarland, Genetics Institute, Saarbrücken, Germany;
[47]Department of Pediatrics, University Hospital Schleswig-Holstein, Campus Kiel, Kiel, Germany

List of principal investigators of the ICGC-DE Mining Consortium

Reiner Siebert[1], Lorenz Trümper[2], Roland Eils[3], Steve Hoffmann[4-6], Christoph Plass[7], Guido Sauter[8], Jan Korbel[9], Peter Lichter[10]

[1]Institute of Human Genetics, University of Ulm and University Hospital of Ulm, Ulm, Germany
[2]Department of Hematology and Oncology, Georg-August-University of Göttingen, Göttingen, Germany;
[3]Division of Theoretical Bioinformatics (B080), German Cancer Research Center (DKFZ), Heidelberg, Germany;
[4]Interdisciplinary Center for Bioinformatics, University of Leipzig, Leipzig, Germany;
[5]Bioinformatics Group, Department of Computer, University of Leipzig, Leipzig, Germany; [6]Transcriptome Bioinformatics, LIFE Research Center for Civilization Diseases, University of Leipzig, Leipzig, Germany
[7]Division of Cancer Epigenomics, German Cancer Research Center (DKFZ), Heidelberg, Germany;
[8]Institute of Pathology, University Medical Center Hamburg-Eppendorf, Martinistr. 52, 20246, Hamburg, Germany
[9]EMBL Heidelberg, Genome Biology, Heidelberg, Germany;
[10]German Cancer Research Center (DKFZ), Division of Molecular Genetics, Heidelberg, Germany.

Samples and cohorts

The data shown in the main text of this manuscript is based on whole genome sequencing (WGS) data from 181 lymphomas, of which 179 had matched normal controls, of a cohort of adult patients from the ICGC MMML-seq consortium. Fig. S1 gives an overview of small variant statistics for this cohort, Suppl. Table 1 summarizes sample specific and QC data, and Suppl. Table 3 displays further statistics on mutational load. Another subgroup of the ICGC MMML-seq consortium consists of 39 pediatric Burkitt lymphomas (BLs) and one adult BL, of which also WGS with matched normal controls is available [1]. The BLs are further subgrouped into BL_solid (28 cases), BL_leukemia (9 cases) and BL_pleura (3 cases). In order to increase statistical power, some of the discovery analyses presented in this manuscript were performed on a merged cohort containing all 219 samples enumerated above (Suppl. Table 2).

In the following, we call the cohort presented in this manuscript the non-BL cohort. For the main figures of the manuscript, the results obtained from the merged cohort were then reduced to and displayed only for the non-BL cohort. Complementary and matching Suppl. Figures display the data for the merged original cohort of the ICGC MMML-seq. This applied to the following correspondences:

- Fig. 2C – Fig. S3
- Fig. 4A – Fig. S12A

Definition of switch regions in the IGH locus

In order to study enrichment and depletion patterns of mutational processes in gcBCL, a comprehensive annotation of the different parts of the IG loci is mandatory. Yet, to date, no genomic coordinates for the

switch regions in the constant part if IGH are available. Therefore, combining information from different sources (contigs stored in IMGT [2], annotated repeats) and genomic information extracted from our cohort (genomic coordinates of breakpoints of hallmark events, i.e. IG-*MYC*, IG-*BCL2* or IG-*BCL6* translocations, recombinations inside the IG-loci, and Ka-ROIs as extracted from the analysis of SNV density), we derive and propose consensus coordinates for the switch regions (in the order of their genomic coordinates) in hg19:

- IGHA2 (switch α2): chr14:106,055,531-106,057,734
- IGHE (switch ε): chr14:106,069,016-106,072,047
- IGHG4 (switch γ4): chr14:106,092,613-106,097,487
- IGHG2 (switch γ2): chr14:106,111,338-106,115,104
- IGHA1 (switch α1): chr14:106,175,034-106,178,629
- IGHG1 (switch γ1): chr14:106,209,614-106,214,314
- IGHG3 (switch γ3): chr14:106,237,954-106,241,354
- IGHM (switch μ): chr14:106,322,324-106,326,978

The genomic coordinates will also be provided as BED-file and GRanges objects at zenodo.org; the switch regions and their genomic context are displayed in Figs. S6 and S7. A definition of a switch region 5' of IGHD was not possible. No contig from IMGT aligned to the intergenic region between IGHD and IGHM and no hallmark translocations (i.e. involving IGH and either *MYC*, *BCL2* or *BCL6*) nor any other translocation had a breakpoint in this genomic window.

Definition of hypermutated cases

We defined as hypermutated those cases whose SNV mutational load is more than two standard deviations above the mean SNV mutational load over the whole cohort. With this criterion, the cases 4109808 (z = 9.06), 4145528 (z = 7.18), 4199714 (z = 3.82) and 4163639 (z = 2.55) were identified as hypermutated (z-scores indicated in parenthesis). All these cases were DLBCLs.

Kataegis and psichales

Using the same methodology as for our gcBCL cohort, we extracted kataegis and psichales clusters from SNV calls of eight data sets originating from different cancer entities (Fig. S4A and S4B). Across all entities, a highly significant enrichment of SNVs in the psichales clusters in late replicating regions was observed (Fig. S4A, S4B, Suppl. Table 4A). The link between increased mutation density and late replication timing prevails for mutation density in the range of psichales. The very focal phenomenon of kataegis, however, is not enriched in late replicating regions. 145/1,056 (13.7%) of all TSS-distant kataegis clusters and 28/39 (70.8%) of the recurrent TSS-distant kataegis clusters were in the vicinity of known fragile sites as compiled from different sources [3-5].

Non-protein coding genes recurrently affected by mutations in gcBCL

Several non-protein coding genes of the genome were recurrently mutated (Fig. 5). Of these, the highest recurrence rates were observed for the long non-coding RNA (lncRNA) RP11-211G3.2, which is located within the first intron of *BCL6* and affected by *BCL6* hypermutation, the known target of aberrant hypermutation microRNA *MIR142* [6] (44%), and the lncRNA genes *MALAT1* (27%) and *NEAT1* (24%). These lncRNAs are adjacent (less than 70 kb distance) on chromosome 11 and affected by HbP with *MALAT1* being the subject and *NEAT1* being the object. MALAT1 and NEAT1 are described to play a role in nuclear body organization [7] and both have been implicated with cancers [8-13]. *MALAT1* has been identified as AID target [14].

Addendum hypermutation by proxy

A pair of Kataegis-regions in a HbP relationship affects *S1PR2* and *DNMT1* (18 and 6 gcBCL with kataegis clusters, respectively), where *S1PR2* hosts the subject and *DNMT1* the object (Fig. 3B-D). Remarkably, these genes neigbouring head-to-tail in the genome have been reported to generate a chimeric transcript [15]. In line, we here detected various tandem RNA chimeras of these strongly coexpressed genes in both GC B cells and gcBCL (Fig. 3D) and confirmed the existence of various RNA chimeras in the DLBCL cell line SU-DHL-10 (Fig. S18). It is intriguing to speculate that the tight co-expression of these genes is a means to link S1PR2-mediated homing of B cells in the GC with the

widespread DNA methylation changes occurring in GC B cells [16,17]. Given the observation of the S1PR2/DNMT1 RNA chimera we wondered whether there is an increased frequency of such transcript chimerism between genes prone to undergo kataegis. Remarkably, even after limiting the analysis to events affecting two different chromosomes, i.e., inter-chromosomal RNA chimera, we detected an increased number between pairs of kataegis regions as compared to a permutation of matched background regions (z-score 8.91, $p = 2.67 \times 10^{-19}$). A possible explanation for the enrichment of these kataegis-associated RNA chimeras might be that regions undergoing such kataegis are more closely positioned to each other in the nuclear space of a (malignant) B cell than the background distribution.
Expression of the two genes *S1PR2* and *DNMT1* was lower compared to GC B cells, but the fraction of reads supporting tandem RNA chimeras was comparable (Fig. 3B-D). The one case violating the *by proxy* relationship (i.e. with kataegis in *DNMT1* but not in *S1PR2*) had much less tandem RNA chimera abundance. In two cases bearing deletions of the intergenic region between the annotated transcripts of these two genes, the amount of tandem RNA chimeras was strongly increased.

Differential usage of mutational mechanisms between ABC- and GCB-DLBCL
Ongoing immunoglobulin somatic mutation has been observed in GCB-DLBCL but not in ABC-DLBCL [18]. Although most ABC-DLBCLs express IgM, and hence do not have undergone successful CSR, many show clonal as well as subclonal internal switch region deletions and point mutations in the switch regions [19]. This indicates repeated and prolonged CSR activity in ABC-DLBCL and their precursor cells, which is not seen in GCB-DLBCL, where ongoing SHM is more prominent. Thus, as there is apparently more and continued activity of the CSR machinery in ABC-DLBCL acting on the IGH locus, we wondered whether this also leads to more CSR-like events in ABC-DLBCL in general and analyzed distributions of kataegis clusters by one-sided tests. Strikingly, ABC-DLBCLs had higher counts (n.s.) and higher fractions of SNVs in CSR-like kataegis clusters, whereas GCB-DLBCLs had higher counts of SNVs in SHM-like kataegis clusters. The ratio of SNVs in CSR-like over SHM-like kataegis clusters showed a strong trend towards higher values in ABC-DLBCLs. Differential usage of mutational mechanisms thus has a correlate in the gene expression derived ABC/GCB-classification (Fig. S2E).

Addendum mutational signatures
In an unsupervised analysis of mutational signatures with NMF, the optimal factorization rank was found to be 11, corresponding to 11 mutational signatures. Eight of those were described before [20] (labeled "AC" for "Alexandrov COSMIC"), including four of six signatures previously identified in gcBCL (Suppl. Table 9). In addition to known signatures, 3 new mutational signatures were discovered in this unsupervised analysis, termed L1, L2 and L3 (Fig. 4A-B). Fig. S11A visualizes the signatures extracted at varying factorization ranks and their similarities in a Sankey diagram. Fig. S11B illustrates the quality criteria for the determination of the optimal factorization rank.
Following the unsupervised analysis, a supervised analysis with signature specific cutoffs using the software package YAPSA was performed (similar to the analysis as described in Sahm et al. [21]) [22]. Making use of the signature-specific cutoffs, which were trained on the same dataset as the initial discovery of the mutational signatures by Alexandrov et al. [20], adds sensitivity to the analysis and offers the potential to analyze smaller cohorts or single samples. In the supervised analysis, additional mutational signatures were identified, increasing the total number to 14 signatures. This fixed set of 14 mutational signatures and the exposures determined by the supervised analysis were used throughout all subsequent analyses presented here.

NMF consensus clustering
Recently, Chapuy et al. [23] performed non-negative matrix factorization (NMF) on merged binarized data from different sources in order to achieve a consensus clustering, which identified five robust clusters. Their analysis was based on 158 genetic driver alterations identified from 304 DLBCL cases by Whole Exome Sequencing (WES). In another analysis, Schmitz et al. [24] found four different prominent genetic subtypes of DLBCL were identified using a custom algorithm. Even though there exists some overlap between the genomic classifications described in the two publications, there are discrepancies between both reports as groups C2 and C4 by Chapuy et al. do not have a counterpart in the classification by Schmitz et al. Additionally, roughly half of the cases are not genomically classified

in the latter article.
In order to compare our data to these results, we also binarized the different classes of mutations (SNVs, small Indels, SVs and CNAs) identified in driver genes and recurrently mutated genes and ran an NMF analysis. In a first step, this analysis was restricted to the DLBCL cases of our gcBCL cohort (initially 76 cases but 72 after excluding four hypermutated cases, defined by mutational load more than two standard deviations above mean SNV mutational load). The results of this analysis are shown in Fig. S15. Based on the criteria for the determination of the optimal factorization rank described in the methods section of our manuscript, the optimal number of clusters was determined to be four in our analysis of DLBCL cases. In analogy to Chapuy et al. and Schmitz et al., these clusters were labelled by the most informative representative of the genes recurrently affected in the respective clusters (Fig. S15A). Our first cluster was thus labelled "MYD88-like", it is dominated by ABC-DLBCL and corresponds to Cluster 5 in Chapuy et al. and the "MCD" subtype in Schmitz et al. (of note, CD79B is also part of the genes characterizing this cluster); our second cluster is labelled "BCL2-like", samples belonging to this cluster are mainly GCB-DLBCL and this cluster corresponds to Cluster 3 in Chapuy et al. and the "EZB" subtype in Schmitz et al. (of note, EZH2 is also among the genes mutated in this cluster); our third cluster is labelled "BCL6-like", samples belonging to this cluster are mixed between the ABC and GCB subtypes with a slight dominance of ABC, this cluster potentially corresponds to Cluster 1 in Chapuy et al. and the "BN2" subtype in Schmitz et al.; and finally a "TP53-like" cluster, dominated by the GCB subtype, overlapping with Clusters 4 and 2 in Chapuy et al. Of note, the genetic subtype termed "N1" in Schmitz et al. was retrieved neither in in the analysis by Chapuy et al., nor in our analysis.
Chapuy et al. also investigated overall mutational density and found that Cluster 4 (corresponding to a subcluster of our "TP53-like" cluster) had the highest mutational density. In our analysis, the "TP53-like" and the "BCL6-like" clusters do not have a significantly different mutational density, but both have a significantly higher mutational density than the "MYD88-like" cluster (Fig. S15B). The WGS data (in contrast to the WES data obtained by Chapuy et al. and Schmitz et al.) additionally allows to compare the fraction of aberrant copy number states across the whole genome (see Fig. S15C). By this approach we can show that the "MYD88-like cluster" also has the lowest fraction of genomic imbalances.
In contrast to Chapuy et al. and Schmitz et al., our cohort offers the potential to analyze the merged cohort of different gcBCL entities (other than BL). In our view this adds important information because – as also discussed by Chapuy et al. – some DLBCL genomic subgroups resemble genomic features of e.g. follicular lymphoma. We thus performed an NMF-based consensus clustering on binarized data also on our merged cohort. Results of this analysis are shown in Fig. S16.
In this merged analysis the optimal number of clusters was determined to be nine, thereby revealing a more detailed substructure of gcBCL entities. Again, clusters were labelled by the most informative representative of the recurrently affected genes inside the cluster. The "MYD88-like", "BCL2-like", "BCL6-like" and "TP53-like" clusters identified in only DLBCL were also retrieved in this larger and more heterogeneous cohort.
In addition, new clusters, which were either completely or at least in their majority driven by FLs, were identified: a "CSMD1-like" cluster, a "PAX5-like" and a "SOCS1-like" cluster. Furthermore, as expected, the "BCL2-like" cluster already discovered in DLBCL was populated by a majority of FLs in this pooled analysis. However, also additional mixed clusters were discovered: a "B2M-like" cluster and a "PIM1-like" cluster, the latter being populated by a high fraction of FL-DLBCL cases.
In order to link these clusters to the mutational mechanisms identified in our manuscript, we performed enrichment and depletion analyses. Results are shown in Fig. 4G-H. This analysis revealed that L1 (CSR) was enriched in the "PIM1-like", "BCL2-like" and "MYD88-like" clusters, whereas L2 (SHM) was enriched in "B2M-like" and "BCL2-like" clusters. Of note, AC9 was depleted in "BCL2-like", "PAX5-like", "BCL6-like" and "PIM1-like" clusters but had high contributions in the "B2M-like", "SOCS1-like" and "MYD88-like" clusters. The ageing signature AC1 was enriched in the "BCL2-like" consensus cluster. Significant differences in total SNV load were found among the different consensus clusters (Kruskal-Wallis discovery test $p = 4.56 \times 10^{-8}$), with pairwise post-hoc tests (Wilcoxon rank sum tests) revealing significantly lower SNV load in the "BCL2-like" consensus cluster than in all others except "CSMD1-like" and "MYD88-like" (Suppl. Table S12A). Furthermore, the "MYD88-like" consensus cluster had significantly less SNV load than the "PIM1-like" and "PAX5-like" clusters and a trend towards less SNV load than the "TP53-like" and the "B2M-like" consensus clusters (p-values are shown in Suppl. Table

S12A). Similarly, significant differences in the aberrant fraction of the genome were found among the different consensus clusters (Kruskal-Wallis discovery test $p = 1.50\times10^{-3}$), with pairwise post-hoc tests (Wilcoxon rank sum tests) revealing significantly lower aberrant fractions in the “BCL2-like” consensus cluster than in the “PIM1-like” and the “SOCS1-like” consensus clusters (Fig S16C and Suppl. Table S12B). Furthermore, the “BCL2-like” consensus cluster had a trend towards lower aberrant fraction than the “TP53-like” consensus cluster. We furthermore investigated congruence and cross-over of the DLBCL cases in our gcBCL cohort between the consensus clusters extracted only among the DLBCLs (Fig. S15) and those consensus clusters extracted among all gcBCL cases (Fig. S16). Numbers are displayed in Suppl. S12C.

Supplementary references

1. López C, Kleinheinz K, Aukema SM, Rohde M, Bernhart SH, Hübschmann D*, et al.* Genomic and transcriptomic changes complement each other in the pathogenesis of sporadic Burkitt lymphoma. Nat Commun 2019; 10**:** 1459-1459.
2. Giudicelli V, Duroux P, Ginestoux C, Folch G, Jabado-Michaloud J, Choume D*, et al.* IMGT/LIGM-DB, the IMGT(R) comprehensive database of immunoglobulin and T cell receptor nucleotide sequences. Nucl Acids Res 2006; 34**:** D781-D784.
3. Arlt MF, Casper AM, Glover TW. Common fragile sites. Cytogen Genome Res 2003; 100**:** 92-100.
4. Glover TW, Wilson TE, Arlt MF. Fragile sites in cancer: more than meets the eye. Nat Rev Cancer 2017; 17**:** 489-501.
5. Ma K, Qiu L, Mrasek K, Zhang J, Liehr T, Quintana LG*, et al.* Common fragile sites: genomic hotspots of DNA damage and carcinogenesis. Int J Mol Sci 2012; 13**:** 11974-11999.
6. Robbiani DF, Bunting S, Feldhahn N, Bothmer A, Camps J, Deroubaix S*, et al.* AID produces DNA double-strand breaks in non-Ig genes and mature B cell lymphomas with reciprocal chromosome translocations. Mol Cell 2009; 36**:** 631-641.
7. West JA, Davis CP, Sunwoo H, Simon MD, Sadreyev RI, Wang PI*, et al.* The long noncoding RNAs NEAT1 and MALAT1 bind active chromatin sites. Mol Cell 2014; 55**:** 791-802.
8. Ellis MJ, Ding L, Shen D, Luo J, Suman VJ, Wallis JW*, et al.* Whole-genome analysis informs breast cancer response to aromatase inhibition. Nature 2012; 486**:** 353-360.
9. Mello SS, Sinow C, Raj N, Mazur PK, Bieging-Rolett K, Broz DK*, et al.* Neat1 is a p53-inducible lincRNA essential for transformation suppression. Genes Dev 2017; 31**:** 1095-1108.
10. Tripathi V, Shen Z, Chakraborty A, Giri S, Freier SM, Wu X*, et al.* Long Noncoding RNA MALAT1 Controls Cell Cycle Progression by Regulating the Expression of Oncogenic Transcription Factor B-MYB. PLos Genetics 2013; 9**:** e1003368-e1003368.
11. Xu C, Yang M, Tian J, Wang X, Li Z. MALAT-1: A long non-coding RNA and its important 3′ end functional motif in colorectal cancer metastasis. Int J Oncol 2011; 39**:** 169-175.
12. YiRen H, YingCong Y, Sunwu Y, Keqin L, Xiaochun T, Senrui C*, et al.* Long noncoding RNA MALAT1 regulates autophagy associated chemoresistance via miR-23b-3p sequestration in gastric cancer. Mol Cancer 2017; 16**:** 174-174.
13. Zhang J, Li Y, Dong M, Wu D. Long non-coding RNA NEAT1 regulates E2F3 expression by competitively binding to miR-377 in non-small cell lung cancer. Oncol Let 2017; 14**:** 4983-4988.
14. Kato L, Begum NA, Burroughs AM, Doi T, Kawai J, Daub CO*, et al.* Nonimmunoglobulin target loci of activation-induced cytidine deaminase (AID) share unique features with immunoglobulin genes. Proc Natl Acad Sci USA 2012; 109**:** 2479-2484.
15. Greger L, Su J, Rung J, Ferreira PG, Lappalainen T, Dermitzakis ET*, et al.* Tandem RNA chimeras contribute to transcriptome diversity in human population and are associated with intronic genetic variants. PLos One 2014; 9**:** e104567-e104567.
16. Kulis M, Merkel A, Heath S, Queirós AC, Schuyler RP, Castellano G*, et al.* Whole-genome fingerprint of the DNA methylome during human B cell differentiation. Nat Genetics 2015; 47**:** 746-756.
17. Muppidi JR, Schmitz R, Green JA, Xiao W, Larsen AB, Braun SE*, et al.* Loss of signalling via Gα13 in germinal centre B-cell-derived lymphoma. Nature 2014; 516**:** 254-258.
18. Lossos IS, Alizadeh aa, Eisen MB, Chan WC, Brown PO, Botstein D*, et al.* Ongoing immunoglobulin somatic mutation in germinal center B cell-like but not in activated B cell-like diffuse large cell lymphomas. Proc Natl Acad Sci USA 2000; 97**:** 10209-10213.
19. Lenz G, Nagel I, Siebert R, Roschke AV, Sanger W, Wright GW*, et al.* Aberrant immunoglobulin class switch recombination and switch translocations in activated B cell–like diffuse large B cell lymphoma. J Exp Med 2007; 204**:** 633-643.
20. Alexandrov LB, Nik-Zainal S, Wedge DC, Aparicio SaJR, Behjati S, Biankin AV*, et al.* Signatures of mutational processes in human cancer. Nature 2013; 500**:** 415-421.
21. Sahm F, Toprak UH, Hübschmann D, Kleinheinz K, Buchhalter I, Sill M*, et al.* Meningiomas induced by low-dose radiation carry structural variants of NF2 and a distinct mutational signature. Acta Neuropathol 2017; 134**:** 155-158.
22. Hübschmann D, Jopp-Saile L, Andresen C, Kramer S, Gu Z, Heilig CE*, et al.* Analysis of mutational signatures with yet another package for signature analysis. Genes Chromosomes Cancer 2020; early online, Nov. 22.
23. Chapuy B, Stewart C, Dunford AJ, Kim J, Kamburov A, Redd RA*, et al.* Molecular subtypes of diffuse large B cell lymphoma are associated with distinct pathogenic mechanisms and outcomes. Nat Med 2018; 24**:** 679-690.
24. Schmitz R, Wright GW, Huang DW, Johnson CA, Phelan JD, Wang JQ*, et al.* Genetics and pathogenesis of diffuse large B-cell lymphoma. N Engl J Med 2018; 378**:** 1396-1407.
