# Supplementary material for: Mutational mechanisms shaping the coding and noncoding genome of germinal center derived B-cell lymphomas
Source: Leukemia. 2021 May 5;35(7):2002–16. doi: 10.1038/s41375-021-01251-z (PMC8257491; doi:10.1038/s41375-021-01251-z)
Supplement: Supplementary file 3 — Supplemental Figures [file 41375_2021_1251_MOESM3_ESM.pdf]

Hübschmann et al., Mutational mechanisms shaping the coding and non-coding genome of germinal center derived B-cell lymphomas

Supplemental figures

Legends:

Figure S1: (A) Per sample mutation statistics: number of coding small variants (SNVs and small indels), number of non-coding small variants, ratio of coding vs. non-coding small variants, number of structural variants, and aberrant genome fraction. The number of noncoding small variants and the ratio of coding vs. non-coding small variants are not shown for two samples without matched normal control. **(B)** Scatterplot of the number of SVs vs. small variant count. **(C - E)** Integrative plots of recurrent CNAs for FL (C), FL-DLBCL (D), DLBCL (E). Non-diploid cases were excluded.

Figure S2: (A) Additional rainfall plots with increasing mutational load from top to bottom. Genomic regions affected by Psichales appear as vertical streaks, but less focalized than the kataegis-like clusters or rainfalls. As in Figure 1, special genomic regions including those targeted by hallmark translocations are highlighted. **(B)** Overlayed violin plots and boxplots displaying distributions of different per sample quantities across gcBCL subgroups: (i) number of SNVs; (ii) number of kataegis clusters; (iii) number of affected Kataegis regions inside the IG loci; (iv) number of affected Kataegis regions outside the IG loci; (v) overall number of affected Kataegis regions; (vi) counts of SNVs inside kataegis clusters; (vii) ratio of affected kataegis regions inside the IG loci vs. all affected Kataegis regions (*ROIs_Ka_ratio*); (viii) counts of SNVs inside Psichales clusters; (ix) counts of SNVs inside affected Psichales regions; and (x) ratio of affected kataegis regions vs. affected Psichales regions. DLBCLs, FL/DLBCLs and FLs had comparable numbers of psichales clusters and affected psichales regions, but DLBCLs and FL/DLBCLs exhibited significantly higher numbers of SNVs in psichales clusters and regions than FLs, reflecting their overall higher mutational load. **(C)** Overlayed violin plots and boxplots displaying distributions of different per sample quantities across gcBCL subgroups: (i) counts of SNVs in kataegis clusters of type "other" (*SNVs_Mech_other*); (ii) counts of SNVs in CSR-like kataegis clusters (*SNVs_Mech_CSR*); (iii) counts of SNVs in SHM-like Kataegis clusters (*SNVs_Mech_SHM*); (iv) ratio of counts of SNVs in Kataegis clusters of type "other" vs. counts of SNVs in all kataegis clusters (*SNVs_Mech_other_ratio*); (v) ratio of counts of SNVs in CSR-like kataegis clusters vs. counts of SNVs in all kataegis clusters (*SNVs_Mech_CSR_ratio*); (vi) ratio of counts of SNVs in SHM-like Kataegis clusters vs. counts of SNVs in all kataegis clusters (*SNVs_Mech_SHM_ratio*). **(D)** The first four items (i) – (iv) are analogous to the first, second, fifth and sixth item of (B), but for DLBCL subtypes. (v) counts of SNVs inside kataegis clusters overlapping with kataegis regions; (vi) ratio of SNVs in kataegis cluster overlapping wiht kataegis regions vs. SNVs in all kataegis clusters; (vii) same ratio as in (vi), but restricted to the IG loci. **(E)** The first two items are analogous to the second and third item in (C), but for DLBCL subtypes; (iii) ratio of counts of SNVs in CSR-like kataegis clusters vs. counts of SNVs in SHM-like kataegis clusters. **(F)** Clustering of the kataegis clusters according to their contributions from CSR-like and SHM-like mutational processes with contributions of SHM and CSR as axes (analogous to Figure 2B, but without Kataegis cluster located inside IG loci). Kataegis clusters dominated by a CSR-like pattern are colored in orange, clusters dominated by a SHM-like pattern are colored in green and clusters dominated by neither pattern (other) are colored in purple.

Figure S3: Kataegis clusters and kataegis regions displayed as oncoprint, analogous to Figure 2C. The x-axis encodes samples, the y-axis the kataegis regions, which are ordered by recurrency of affection. The oncoprint carries 14 layers of annotation: i) the contributions per kataegis region of the three different categories SHM-like (green), CSR-like (orange) and other (purple) as stacked barplots; ii) a categorical annotation whether the kataegis region is located in VDJ genes (blue) or other parts of the IG loci (turquoise), whether it was described to be a target of aberrant SHM before (purple), whether the closest gene was associated with lymphomagenesis in some fashion (red), whether the closest gene is a cancer gene (orange) or all others (yellow); iii) barplots indicating the total number of SNVs in the respective kataegis region cohort-wide; iv) the density of SNVs per genomic window (SNVs per bp); v) genomic size of the respective kataegis region in bp; vi) distance to the closest transcription start site; vii) replication timing (encoded by RepliSeq scores) in lymphoblastoid cell lines; viii) integrated subject score of the respective kataegis region; ix) the integrated object score of the respective kataegis region; x) distribution of cellular fractions per kataegis regions; xi) distribution of distances to the closet breakpoint; xii) fractions of the different functional Annovar annotations; xiii) fractions of the different chromatin states from CG B cells; xiv) fractions of SNVs matching the motif DGYW in the respective kataegis regions.

Figure S4: Distribution of replication timing of SNVs in psichales clusters vs. SNVs in background as illustrated by combined boxplots and violin plots. (A) Replication time averaged over cell lines vs. psichales in the different entities (three breast cancer cohorts (BRCA), one cohort of chronic lymphocytic leukemia (CLL), one external DLBCL cohort, two cohorts of ovarian cancer (OV) and one sarcoma (SA) cohort (Nik-Zainal et al., Nature 2016; Nik-Zainal, Cell 2012; Puente et al., Nature 2015; Patch et al., Nature 2015; results shown here are in part based upon data generated by the TCGA research network: http://cancergenome.nih.gov/). (B) Replication time averaged over cell lines vs. psichales across all entities and samples. (C) Comparison of replication time in the breast cancer cell line MCF7 (left) against replication time averaged over cell lines (right) vs. psichales in the breast cancer entities. (D) Comparison of replication time in the lymphoblastic cell lines GM06990, GM12801, GM12812, GM12813 and GM12878 against replication time averaged over cell lines (right) vs. psichales in the B-cell derived entities. The label "TRUE" represents the psichales SNVs, the label "FALSE" the non-psichales SNVs. (E) Distribution of replication timing of SNVs in kataegis clusters vs. SNVs in background as illustrated by combined boxplots and violin plots - in contrast to psichales, kataegis is not enriched in late replicating genomic regions.

Figure S5: Psichales clusters and psichales regions displayed as oncoprint, analogous to Figure S3. 18,794 psichales clusters were detected in 219 genomes. Using a recurrence threshold of three affected cases, 252 recurrent psichales regions were identified. The x-axis encodes samples, the y-axis the psichales regions, which are ordered by recurrency of affection. The oncoprint carries 12 layers of annotation analogous to Figure S3, but without colour coding of the individual psichales clusters and without integrated subject or object scores (all not defined).

Figures S6/S7: Genomic windows in which the IGH switch regions were defined (in the order of their genomic coordinates): **(A)** IGHA2 (switch α2), **(B)** IGHE (switch ε), **(C)** IGHG4 (switch γ4), **(D)** IGHG2 (switch γ2), **(E)** IGHA1 (switch α1), **(F)** IGHG1 (switch γ1), **(G)** IGHG3 (switch γ3), **(H)** IGHM (switch μ). Each subplot is composed of several tracks, showing from bottom to top (if a track is empty it is left out in the respective subplot): (i) transcripts from ENSEMBL; (ii) repeats from repeat masker (with colour coding Alu – light blue, L1 – blue, L2 – light green, L3 – green, L4 – pale red, LINE – light red, LTR – ocre, MER – orange, MIR – pale purple, MLT – purple, RNA – pale yellow, SINE – brown, rich – grey50, oligomer – grey30, monomer – black, other – grey80, simple

- red); (iii) merged repeats whenever overlapping (irrespective of repeat class); (iv – vi) tracks showing alignments with contigs from the IMGT data base where *oGL* = „original global-local“ (global-local alignemntes of contigs known to originate from one single switch region), and *oL* = “original local“ (local Smith-Waterman alignments of contigs known to originate from one single switch region) and *jL* = „junction local“ (local alignments of contigs known to originate from recombinations); (vii) a track for Ka-ROIs (regions of interest of recurrent Kataegis-like clusters); (viii – xii) tracks showing hallmark events (translocations between IGH and *MYC*, *BCL2* or *BCL6*) in the different subgroups, where the subgroups „BL_solid“, „BL_leukemia“ and „BL_pleura“ are grouped together and designated „BL“ and the subgroups „DHL“ and „B-NOS“ are grouped together and designated „other“. All subgroup specific tracks use a common colour coding for the hallmark events: MYC - blue, BLC2 - dark green, BCL6 – brown; (xiii) track showing non-hallmark translocations; and (xiv) track showing coordinates of breakpoints of rearrangements within the IGH locus.

Figure S8: Mutational mechanisms in gcBCL subgroups, comparing DLBCL, FL-DLBCL and FL (left part of the figure, items A, B, E, F, I, J, M and N) and ABC-DLBCL vs. GCB-DLBCL (right part of the figure, items C, D, G, H, K, L, O, P and R). (A) Absolute counts and (B) normalized fractions of CSR-like kataegis clusters among all kataegis clusters in gcBCL subgroups. (E) Absolute counts and (F) normalized fractions of SHM-like kataegis clusters among all kataegis clusters in gcBCL subgroups. (I) Absolute counts and (J) normalized fractions of kataegis clusters of type “other” among all kataegis clusters in gcBCL subgroups. (C) Absolute counts and (D) normalized fractions of CSR-like kataegis clusters among all kataegis clusters in ABC-DLBCL vs. GCB-DLBCL. (G) Absolute counts and (H) normalized fractions of SHM-like kataegis clusters among all kataegis clusters in ABC-DLBCL vs. GCB-DLBCL. (K) Absolute counts and (L) normalized fractions of kataegis clusters of type “other” among all kataegis clusters in ABC-DLBCL vs. GCB-DLBCL. (M) Absolute counts of HbP instances per sample in gcBCL subgroups. (N) Number of HbP instances per sample normalized to the square of the number of kataegis clusters per sample in gcBCL subgroups. (O, P) Analogous to (M) and (N) but for ABC-DLBCL vs. GCB-DLBCL. (Q) Dotplot with linear fit between (on the x-axis) the number of kataegis clusters per sample and (on the y-axis) the square root of the number of HbP instances per sample. The linear fit in this display illustrates a quadratic relationship, i.e. $y = c*x^2$. (R) Enrichment and depletion patterns of mutational signatures in ABC-DLBCL vs. GCB-DLBCL. Abbreviations: HbP: hypermutation by proxy.

Figure S9: Hypermutation by proxy: overview. Pairwise subject-object relationships of the kataegis regions as determined by the custom method introduced in this work. All kataegis regions identified in gcBCL are included in the plot. Subject regions are displayed as rows and object regions as columns. Note that this visualization is not symmetric.

Figure S10: Hypermutation by proxy: examples. (A-I) HbP at the *BCL6* gene cluster. (A) Oncoprint showing the samples affected by rKa-clusters (recurrent Kataegis-like clusters) in *BCL6* and the neighboring genes involved in HbP (*RP11−132N15.3*, *RP11−44H4.1*, *LPP*, *ST6GAL1*). Whereas three different Ka-ROIs are located within *LPP*, the other genes are affected by only one Ka-ROI each, leading to a total of six Ka-ROIs involved in the *BCL6* gene cluster. Four out of six Ka-ROIs overlap with chromatin segments which are annotated as enhancers. (B - E) Scatterplots of the expression of the genes *RP11−132N15.3*, *RP11−44H4.1*, *LPP*, *ST6GAL1* as compared to *BCL6* per subgroup. **(F-I) HbP around *PAX5*.** (F) Oncoprint showing the samples affected by rKa-clusters (recurrent Kataegis-like clusters) in *PAX5* and the neighboring genes involved in HbP (*ZCCHC7*, *GRHPR* and *RP11-397D12.4*). Whereas two different Ka-ROIs are located within *ZCCHC7*, the other genes are affected by only one Ka-ROI each, leading to a total of four Ka-ROIs involved

in genes in the vicinity of *PAX5*. Three out of four Ka-ROIs overlap with chromatin segments which are annotated as enhancers. (G - I) Scatterplots of the expression of the genes *ZCCHC7*, *GRHPR* and *RP11-397D12.4* as compared to *PAX5* per subgroup.

Figure S11: Unsupervised analysis of mutational signatures with NMF. (A) Riverplot graph, in which nodes correspond to mutational signatures and edges represent pairwise cosine similarities between mutational signatures. The vertical direction encodes different factorization ranks, increasing from top to bottom. The horizontal direction shows the different mutational signatures extracted at the respective factorization rank. The horizontal position of one node (corresponding to one mutational signature) is chosen such that crossing edge weights are minimized. Mutational signatures are colored and labeled according to their cosine similarity to known mutational signatures (from http://cancer.sanger.ac.uk/cosmic/signatures). At the optimal factorization rank k = 11 (cf. subplot (B)), three new signatures can be found. These new signatures are taken into account in the coloring and labeling of the entire graph. **(B)** Quality metrics for the choice of the factorization rank: norm of the residuals ("FrobError" for Frobenius reconstruction error, to be minimized), cophenetic correlation coefficient ("copheneticCoeff", to be maximized) and the mean Amari distance ("meanAmariDist", to be minimized). Combining these three measures, an optimal factorization rank of 11 is evaluated. **(C)** Determination of the cutoffs for the stratified analysis of mutational signatures with replication timing (encoded by RepliSeq score) as stratification axis. The vertical red lines show the chosen cutoffs in the density function of the RepliSeq scores of all SNVs of our cohort.

Figure S12: Unsupervised analysis of mutational signatures with NMF. (A) Exposures to the identified mutational signatures for 219 lymphomas (179 cases with matched normal from this study and additional 40 pediatric Burkitt lymphomas (BL), 39 from [Lopez et al., manuscript accepted at Nature Communications] and one adult BL). **(B)** Overlayed violin plots and boxplots of the distributions of cancer cell fractions (CCF) of SNVs in IG const genes, in IG switch regions (where the CSR-like profile stems from) and in IG VDJ genes (where the SHM-like profile stems from). **(C)** Normalized exposures to the identified mutational signatures. **(D - G)** More enrichment and depletion patterns as indicated in the titles of the subplots. **(D)** Stratified analysis of mutational signatures showed an enrichment of AC1 (spontaneous deamination, $p_{KW} = 8.8\times10^{-23}$, $p_{Nem} = 5.8\times10^{-13}$) and AC2 (APOBEC, $p_{KW} = 2.2\times10^{-3}$, $p_{Nem} = 9.7\times10^{-3}$) in early clonal evolution. L1 showed a trend towards enrichment in early ($p_{KW} = 9.6\times10^{-2}$) and AC9 in late ($p_{KW} = 1.6\times10^{-2}$, $p_{Nem} = 0.2$) clonal evolution. **(G)** L1 and L2 were enriched in kataegis clusters, with L1 being enriched in CSR-like ($p_{KW} = 9.8\times10^{-21}$, $p_{Nem} = 1.7\times10^{-5}$) and L2 in SHM-like ($p_{KW} = 4.4\times10^{-35}$, $p_{Nem} = 5.4\times10^{-4}$) kataegis clusters as compared to the non-clustered SNV stratum. Abbreviations: CCF – cancer cell fraction, id. – intermutation distance. Error bars represent standard error of the mean (SEM).

Figure S13: Occurrences of the most frequently mutated genes. (A) In DLBCL vs. FL and (B) in ABC-DLBCL vs. GCB-DLBCL.

Figure S14: Extended SNV statistics for the driver genes. To account for the broad range of mutation frequencies in the different driver genes, the visualization is split into three groups with different axis scales. From left to right, the panels show: i) the number of samples with any SNVs in the respective gene or in a 2.5 kb window around the gene; ii) the number of kataegis clusters overlapping with the respective gene in the gcBCL cohort; iii) the distribution of SNV counts per sample; iv) the distribution of the distance of each SNV to the TSS (of the canonical transcript); v) fractions of SNVs belonging to SHM-like kataegis clusters (green), CSR-like kataegis clusters (orange), kataegis clusters of type "other" (purple) and no kataegis clusters at all (yellow); vi) the

distribution of the position of the SNVs relative to the (canonical) TSS of the respective genes: outside of a 2.5 kb window 5' of the gene (light blue), inside a 2.5 kb window 5' of the gene (dark blue), within 2.5 kb from TSS in the gene body (dark green), more than 2.5 kb away of the TSS in the gene body, and 3' of the gene (light red); and vii) the ratio of the SNV density in the window 2.5 kb around the (canonical) TSS divided by the SNV density in the remaining gene.

Figure S15: NMF-based consensus clustering of alterations in driver genes identified in DLBCL cases of our gcBCL cohort. (A) Four clusters were identified and labelled "MYD88-like" (red), "BCL2-like" (green), "BCL6-like" (light blue) and "TP53-like" (purple) and annotated at the bottom of the heatmap. The cell-of-origin attribution of the DLBCL cases is also annotated below the heatmap. All alterations are encoded as red squares in affected genes. Inside the clusters, affected genes are ordered by occurrence of alterations. (B) Assessment of SNV load for the cases belonging to the different clusters. (C) Fraction of the genome with aberrant copy number state per consensus cluster.

Figure S16: **NMF-based consensus clustering of alterations in driver genes identified in the gcBCL cohort across sub-entities.** (A) Nine clusters were identified and labelled "TP53-like" (red), "B2M-like" (orange), "PIM1-like" (light green), "BCL6-like" (green), "PAX5-like" (turquoise), "BCL2-like" (light blue), "CSMD1-like" (blue), "SOCS1"-like (purple), and "MYD88-like" (magenta), and annotated at the bottom of the heatmap. The cell-of-origin attribution as well as the subgroup/entity of the samples are also annotated below the heatmap. The three hypermutated samples 4145528, 4109808 and 4163639 were excluded from the whole cohort of 181 gcBCL. All alterations are encoded as red squares in affected genes. Inside the clusters, affected genes are ordered by occurrence of alterations. (B) Mutational load in the different consensus clusters. (C) Fraction of the genome with aberrant copy number state.

Figure S17: Distributions of cancer cell fraction per driver gene.

Figure S18: Tandem RNA chimeras between S1PR2 and DNMT1 in the DLBCL cell line SU-DHL-10. A variety of splice variants were identified by PCR.

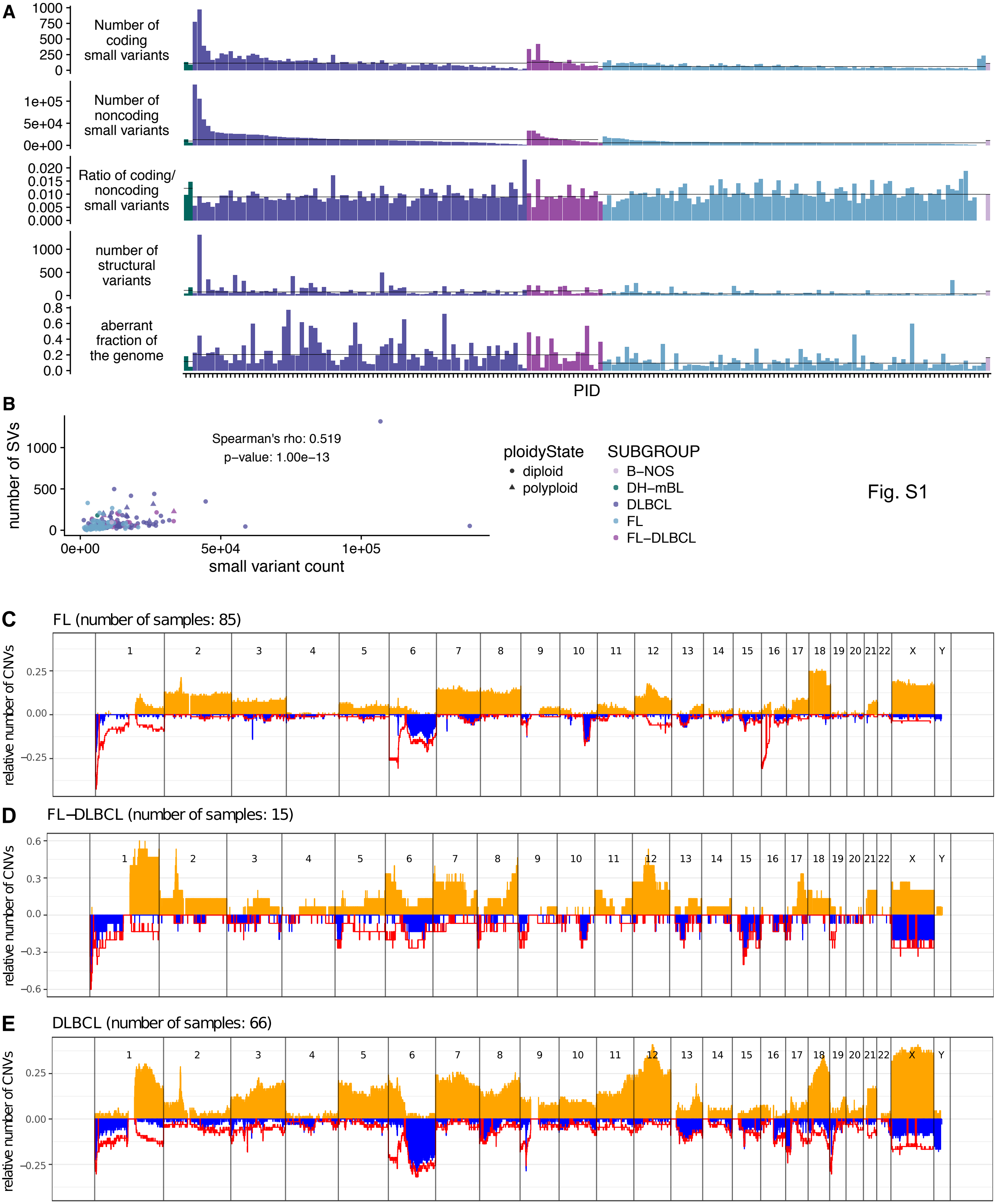
A
Number of coding small variants
Number of noncoding small variants
Ratio of coding/ noncoding small variants
number of structural variants
aberrant fraction of the genome
PID
B
Spearman's rho: 0.519
p−value: 1.00e−13
number of SVs
small variant count
ploidyState
diploid
polyploid
SUBGROUP
B−NOS
DH−mBL
DLBCL
FL
FL−DLBCL
C
FL (number of samples: 85)
D
FL−DLBCL (number of samples: 15)
E
DLBCL (number of samples: 66)
relative number of CNVs

Fig. S1

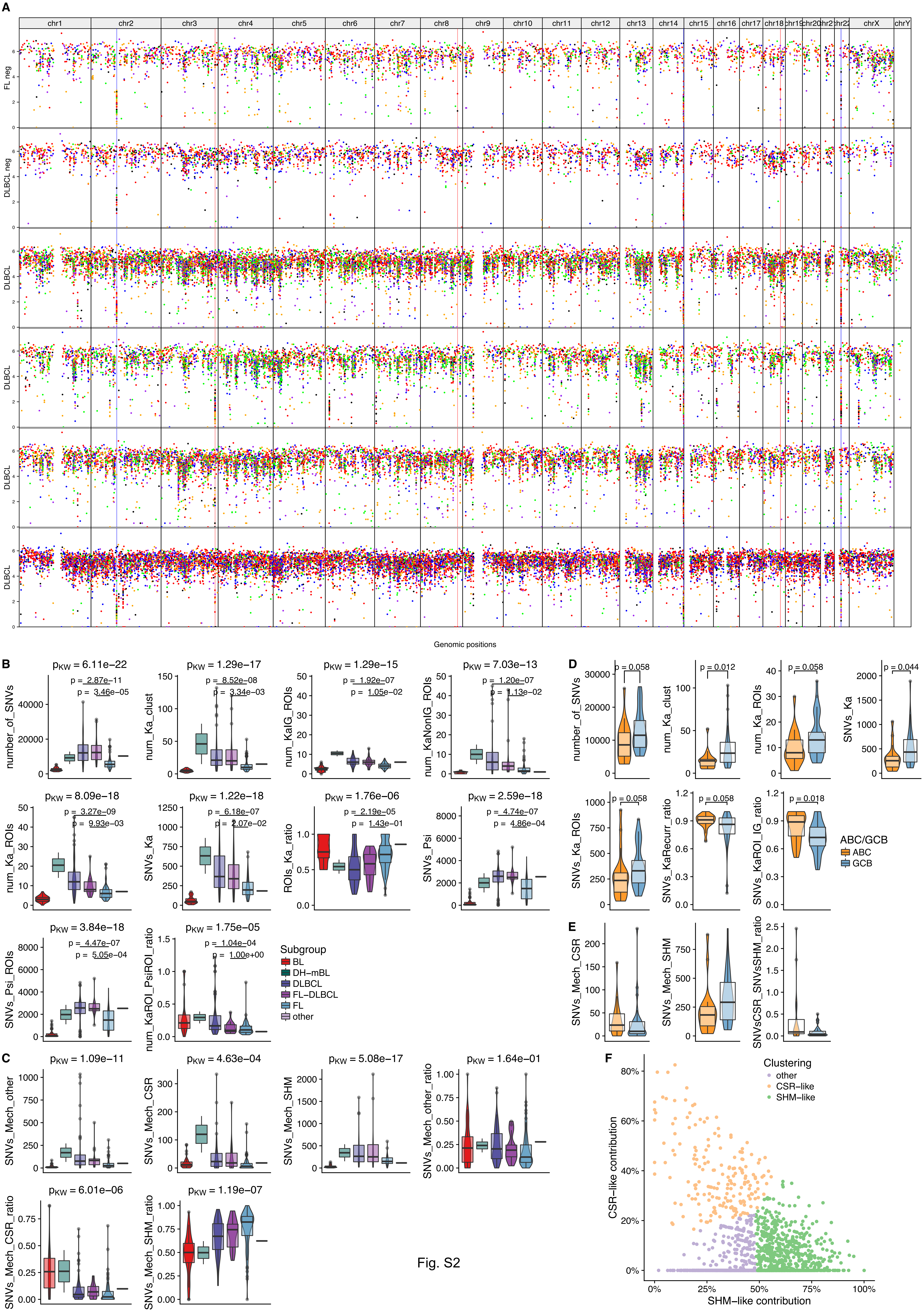
A
chr1
chr2
chr3
chr4
chr5
chr6
chr7
chr8
chr9
chr10
chr11
chr12
chr13
chr14
chr15
chr16
chr17
chr18
chrX
chrY
FL neg
DLBCL neg
DLBCL
Genomic positions
B
pKW = 6.11e−22
p = 2.87e−11
p = 3.46e−05
number_of_SNVs
pKW = 1.29e−17
p = 8.52e−08
p = 3.34e−03
num_Ka_clust
pKW = 1.29e−15
p = 1.92e−07
p = 1.05e−02
num_KaIG_ROIs
pKW = 7.03e−13
p = 1.20e−07
p = 1.13e−02
num_KaNonIG_ROIs
pKW = 8.09e−18
p = 3.27e−09
p = 9.93e−03
num_Ka_ROIs
pKW = 1.22e−18
p = 6.18e−07
p = 2.07e−02
SNVs_Ka
pKW = 1.76e−06
p = 2.19e−05
p = 1.43e−01
ROIs_Ka_ratio
pKW = 2.59e−18
p = 4.74e−07
p = 4.86e−04
SNVs_Psi
pKW = 3.84e−18
p = 4.47e−07
p = 5.05e−04
SNVs_Psi_ROIs
pKW = 1.75e−05
p = 1.04e−04
p = 1.00e+00
num_KaROI_PsiROI_ratio
Subgroup
BL
DH−mBL
DLBCL
FL−DLBCL
FL
other
C
pKW = 1.09e−11
SNVs_Mech_other
pKW = 4.63e−04
SNVs_Mech_CSR
pKW = 5.08e−17
SNVs_Mech_SHM
pKW = 1.64e−01
SNVs_Mech_other_ratio
pKW = 6.01e−06
SNVs_Mech_CSR_ratio
pKW = 1.19e−07
SNVs_Mech_SHM_ratio
D
p = 0.058
number_of_SNVs
p = 0.012
num_Ka_clust
p = 0.058
num_Ka_ROIs
p = 0.044
SNVs_Ka
p = 0.058
SNVs_Ka_ROIs
p = 0.058
SNVs_KaRecurr_ratio
p = 0.018
SNVs_KaROI_IG_ratio
ABC/GCB
ABC
GCB
E
SNVs_Mech_CSR
SNVs_Mech_SHM
SNVsCSR_SNVsSHM_ratio
F
Clustering
other
CSR−like
SHM−like
CSR−like contribution
SHM−like contribution

Fig. S2

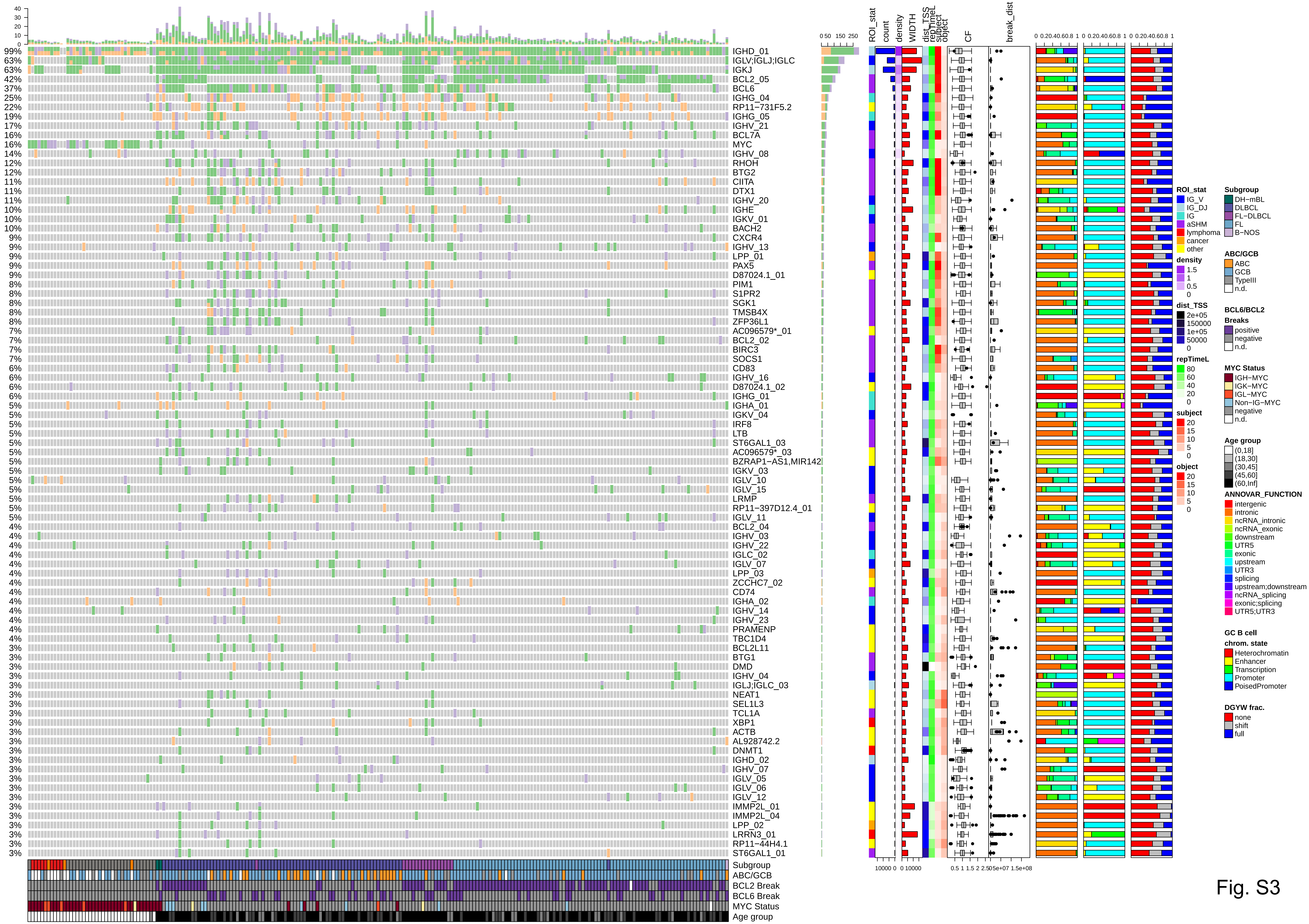

IGHD_01
IGLV;IGLJ;IGLC
IGKJ
BCL2_05
BCL6
IGHG_04
RP11−731F5.2
IGHG_05
IGHV_21
BCL7A
MYC
IGHV_08
RHOH
BTG2
CIITA
DTX1
IGHV_20
IGHE
IGKV_01
BACH2
CXCR4
IGHV_13
LPP_01
PAX5
D87024.1_01
PIM1
S1PR2
SGK1
TMSB4X
ZFP36L1
AC096579*_01
BCL2_02
BIRC3
SOCS1
CD83
IGHV_16
D87024.1_02
IGHG_01
IGHA_01
IGKV_04
IRF8
LTB
ST6GAL1_03
AC096579*_03
BZRAP1−AS1,MIR142
IGKV_03
IGLV_10
IGLV_15
LRMP
RP11−397D12.4_01
IGLV_11
BCL2_04
IGHV_03
IGHV_22
IGLC_02
IGLV_07
LPP_03
ZCCHC7_02
CD74
IGHA_02
IGHV_14
IGHV_23
PRAMENP
TBC1D4
BCL2L11
BTG1
DMD
IGHV_04
IGLJ;IGLC_03
NEAT1
SEL1L3
TCL1A
XBP1
ACTB
AL928742.2
DNMT1
IGHD_02
IGHV_07
IGLV_05
IGLV_06
IGLV_12
IMMP2L_01
IMMP2L_04
LPP_02
LRRN3_01
RP11−44H4.1
ST6GAL1_01
Subgroup
ABC/GCB
BCL2 Break
BCL6 Break
MYC Status
Age group
ROI_stat
count
density
WIDTH
dist_TSS
repTimeL
subject
object
CF
break_dist
ROI_stat
IG_V
IG_DJ
IG
aSHM
lymphoma
cancer
other
density
dist_TSS
repTimeL
subject
object
Subgroup
DH−mBL
DLBCL
FL−DLBCL
FL
B−NOS
ABC/GCB
ABC
GCB
TypeIII
n.d.
BCL6/BCL2 Breaks
positive
negative
n.d.
MYC Status
IGH−MYC
IGK−MYC
IGL−MYC
Non−IG−MYC
negative
n.d.
Age group
(0,18]
(18,30]
(30,45]
(45,60]
(60,Inf]
ANNOVAR_FUNCTION
intergenic
intronic
ncRNA_intronic
ncRNA_exonic
downstream
UTR5
exonic
upstream
UTR3
splicing
upstream;downstream
ncRNA_splicing
exonic;splicing
UTR5;UTR3
GC B cell chrom. state
Heterochromatin
Enhancer
Transcription
Promoter
PoisedPromoter
DGYW frac.
none
shift
full

Fig. S3

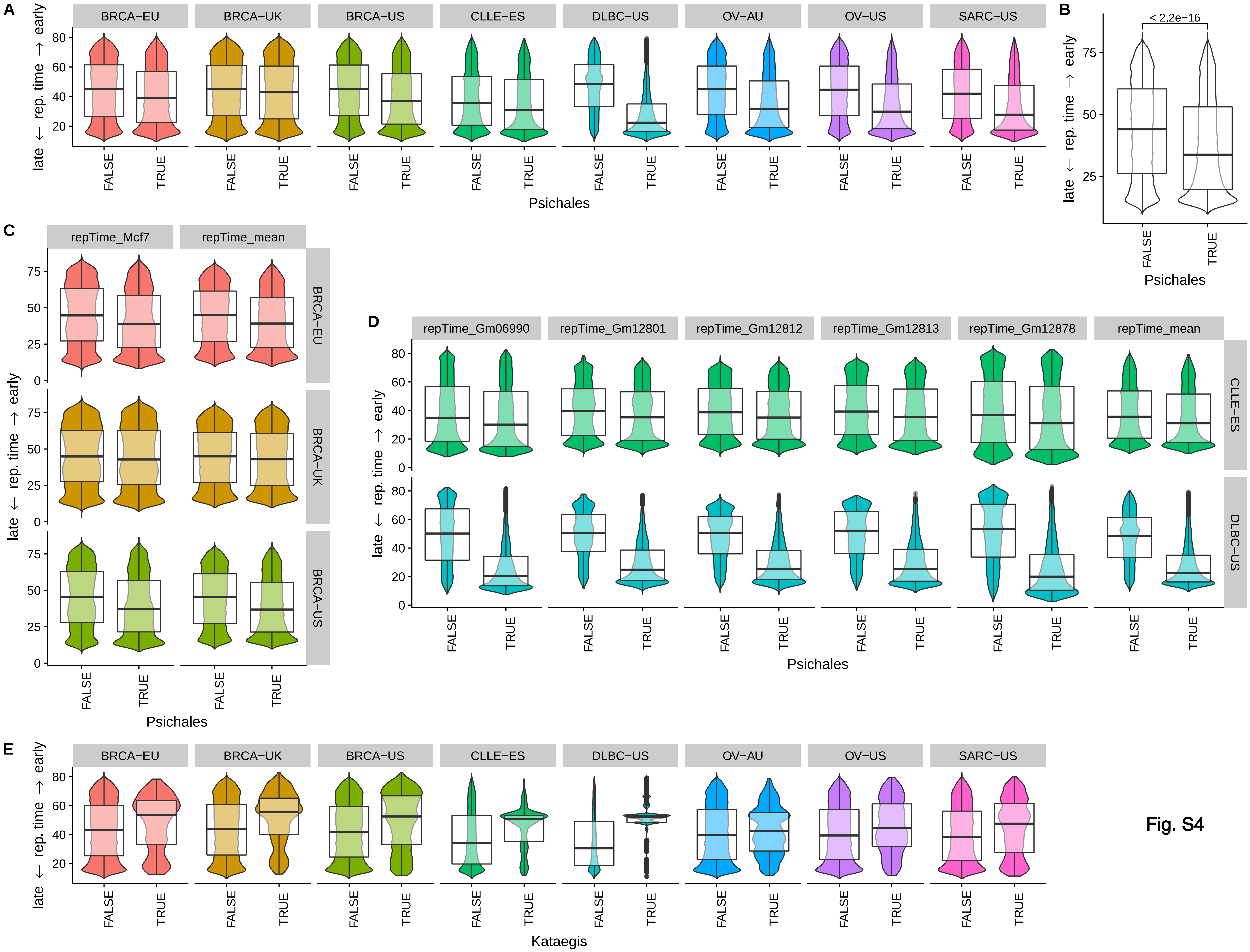

A
BRCA−EU
BRCA−UK
BRCA−US
CLLE−ES
DLBC−US
OV−AU
OV−US
SARC−US
late ← rep. time → early
Psichales
FALSE
TRUE
B
< 2.2e−16
C
repTime_Mcf7
repTime_mean
D
repTime_Gm06990
repTime_Gm12801
repTime_Gm12812
repTime_Gm12813
repTime_Gm12878
E
Kataegis

Fig. S4

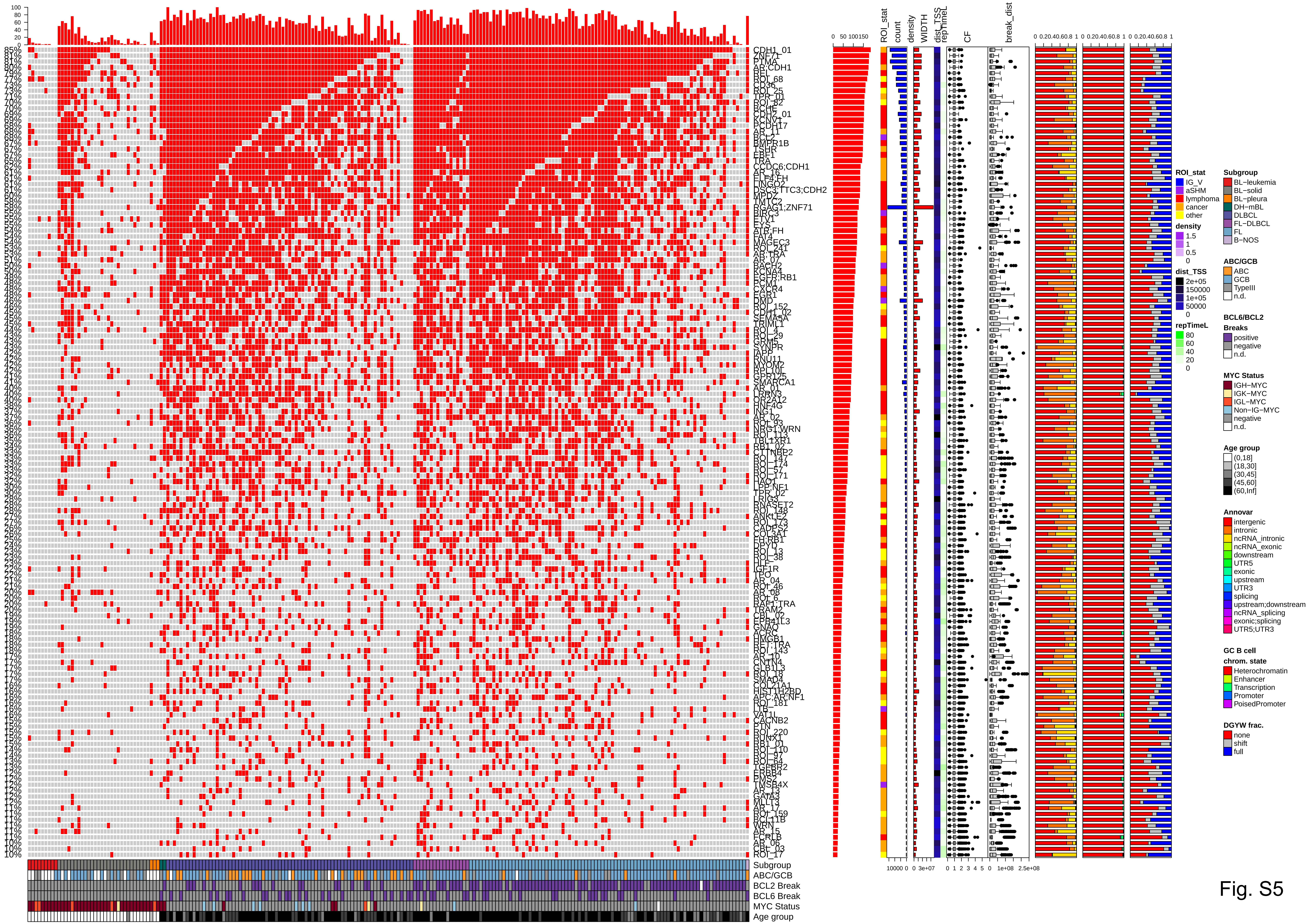
ROI_stat
count
density
WIDTH
dist_TSS
repTimeL
CF
break_dist
ROI_stat
IG_V
aSHM
lymphoma
cancer
other
density
dist_TSS
repTimeL
Subgroup
BL−leukemia
BL−solid
BL−pleura
DH−mBL
DLBCL
FL−DLBCL
FL
B−NOS
ABC/GCB
ABC
GCB
TypeIII
n.d.
BCL6/BCL2
Breaks
positive
negative
n.d.
MYC Status
IGH−MYC
IGK−MYC
IGL−MYC
Non−IG−MYC
negative
n.d.
Age group
(0,18]
(18,30]
(30,45]
(45,60]
(60,Inf]
Annovar
intergenic
intronic
ncRNA_intronic
ncRNA_exonic
downstream
UTR5
exonic
upstream
UTR3
splicing
upstream;downstream
ncRNA_splicing
exonic;splicing
UTR5;UTR3
GC B cell
chrom. state
Heterochromatin
Enhancer
Transcription
Promoter
PoisedPromoter
DGYW frac.
none
shift
full
Subgroup
ABC/GCB
BCL2 Break
BCL6 Break
MYC Status
Age group

Fig. S5

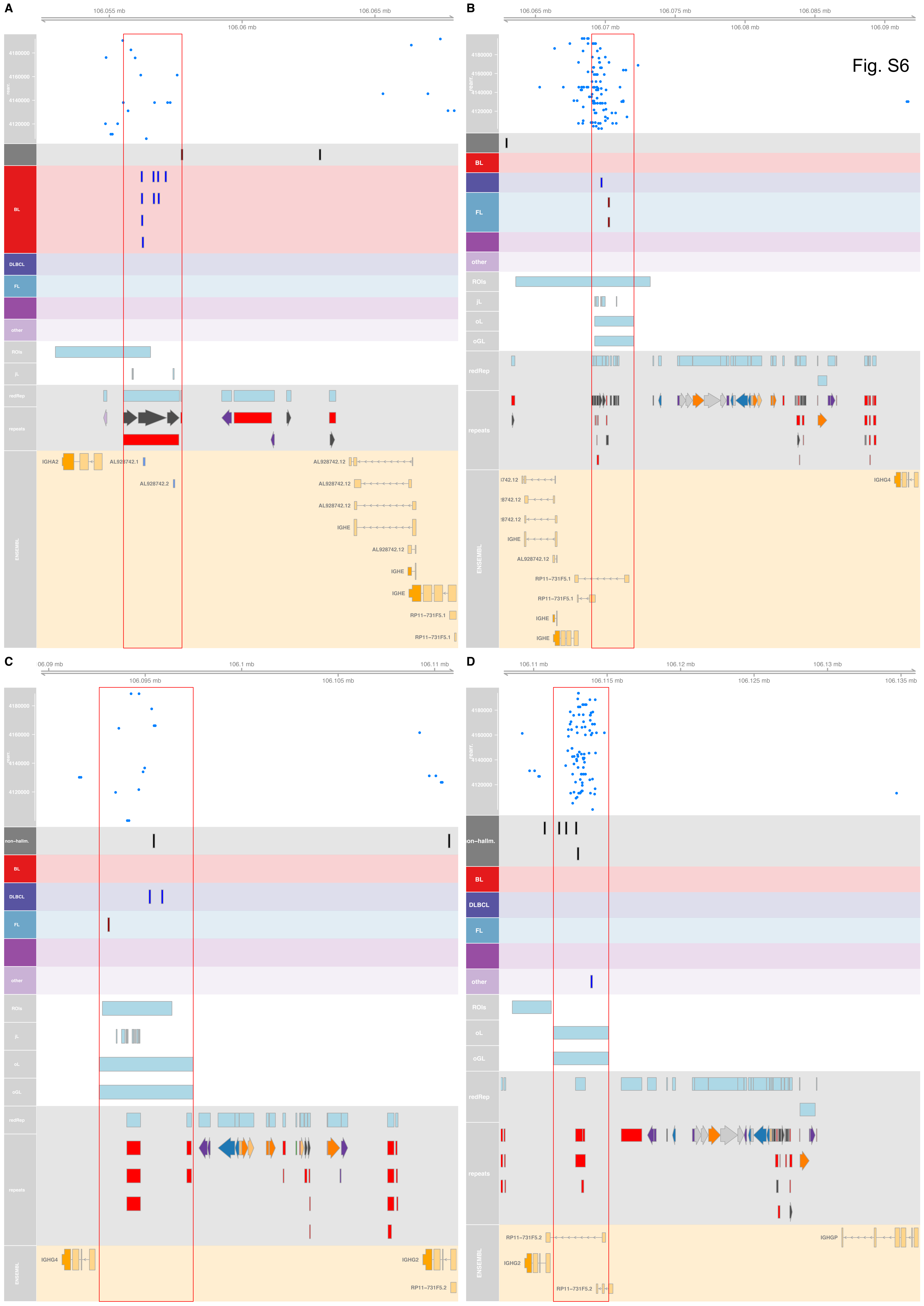
A
B
C
D
Fig. S6
BL
DLBCL
FL
other
ROIs
jL
oL
oGL
redRep
repeats
ENSEMBL
IGHA2
IGHE
IGHG4
IGHG2
IGHGP
AL928742.1
AL928742.2
AL928742.12
RP11-731F5.1
RP11-731F5.2
non-hallm.

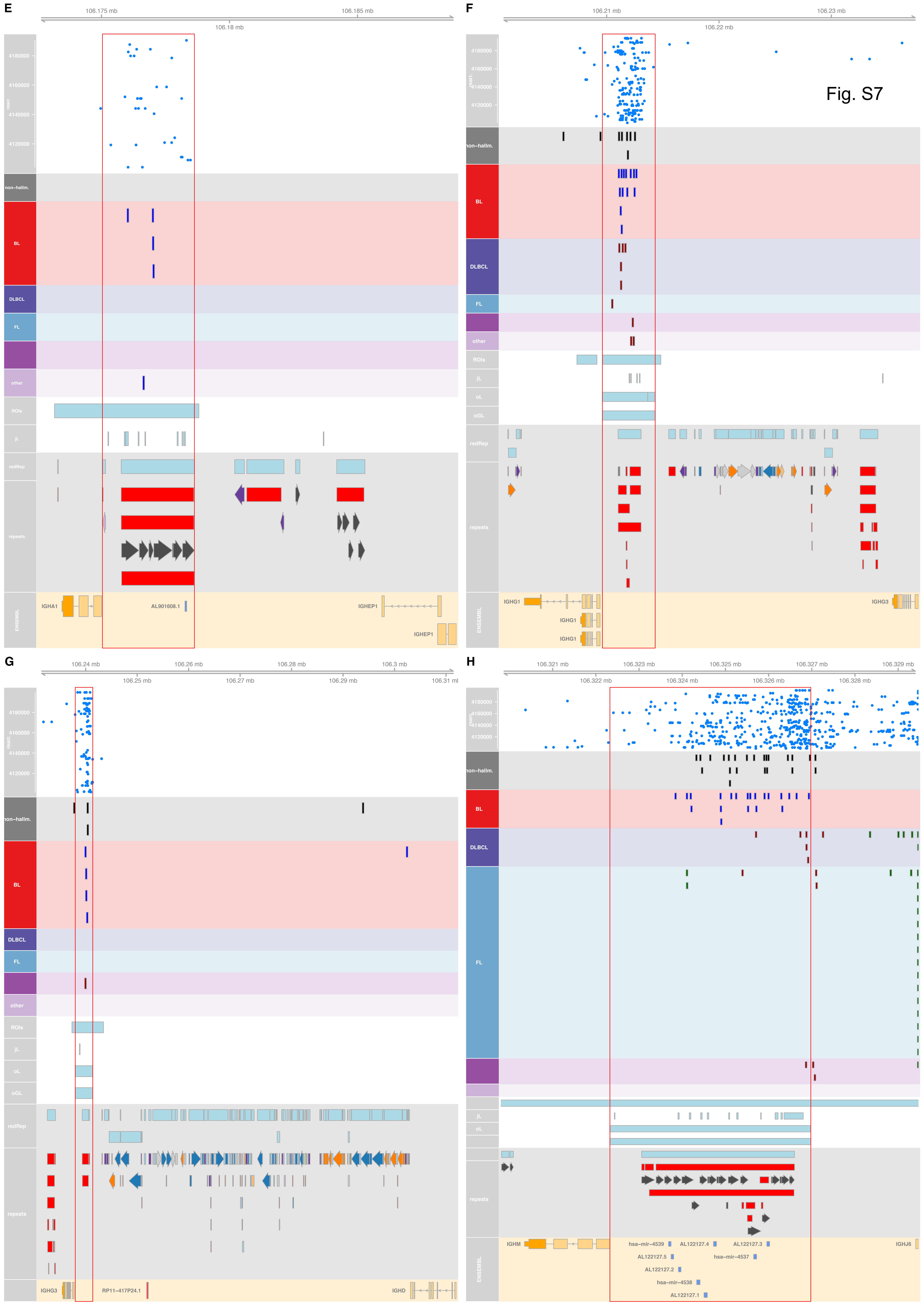
E
106.175 mb
106.18 mb
106.185 mb
4180000
4160000
4140000
4120000
rearr.
non−hallm.
BL
DLBCL
FL
other
ROIs
jL
redRep
repeats
ENSEMBL
IGHA1
AL901608.1
IGHEP1
IGHEP1
F
106.21 mb
106.22 mb
106.23 mb
4180000
4160000
4140000
4120000
rearr.
non−hallm.
BL
DLBCL
FL
other
ROIs
jL
oL
oGL
redRep
repeats
ENSEMBL
IGHG1
IGHG1
IGHG1
IGHG3
G
106.24 mb
106.25 mb
106.26 mb
106.27 mb
106.28 mb
106.29 mb
106.3 mb
106.31 mb
4180000
4160000
4140000
4120000
rearr.
non−hallm.
BL
DLBCL
FL
other
ROIs
jL
oL
oGL
redRep
repeats
IGHG3
RP11−417P24.1
IGHD
H
106.321 mb
106.322 mb
106.323 mb
106.324 mb
106.325 mb
106.326 mb
106.327 mb
106.328 mb
106.329 mb
4180000
4160000
4140000
4120000
rearr.
non−hallm.
BL
DLBCL
FL
jL
oL
repeats
ENSEMBL
IGHM
hsa−mir−4539
AL122127.4
AL122127.3
IGHJ6
AL122127.5
hsa−mir−4537
AL122127.2
hsa−mir−4538
AL122127.1
Fig. S7

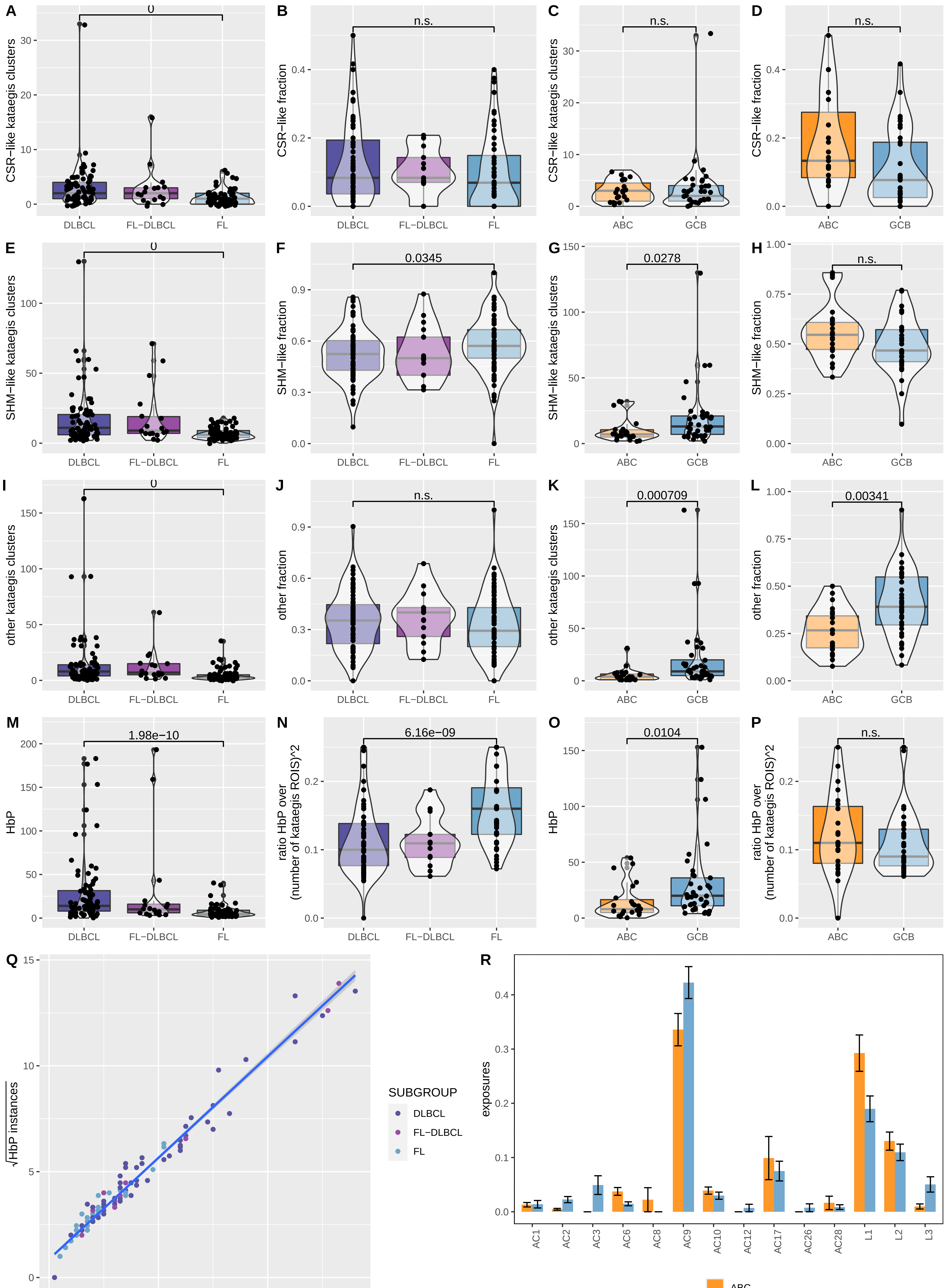

A
B
C
D
E
F
G
H
I
J
K
L
M
N
O
P
Q
R
CSR−like kataegis clusters
CSR−like fraction
SHM−like kataegis clusters
SHM−like fraction
other kataegis clusters
other fraction
HbP
ratio HbP over (number of kataegis ROIS)^2
n.s.
0.0345
0.0278
0.000709
0.00341
1.98e−10
6.16e−09
0.0104
DLBCL
FL−DLBCL
FL
ABC
GCB
√HbP instances
number of kataegis clusters
SUBGROUP
exposures
AC1
AC2
AC3
AC6
AC8
AC9
AC10
AC12
AC17
AC26
AC28
L1
L2
L3

Fig. S8

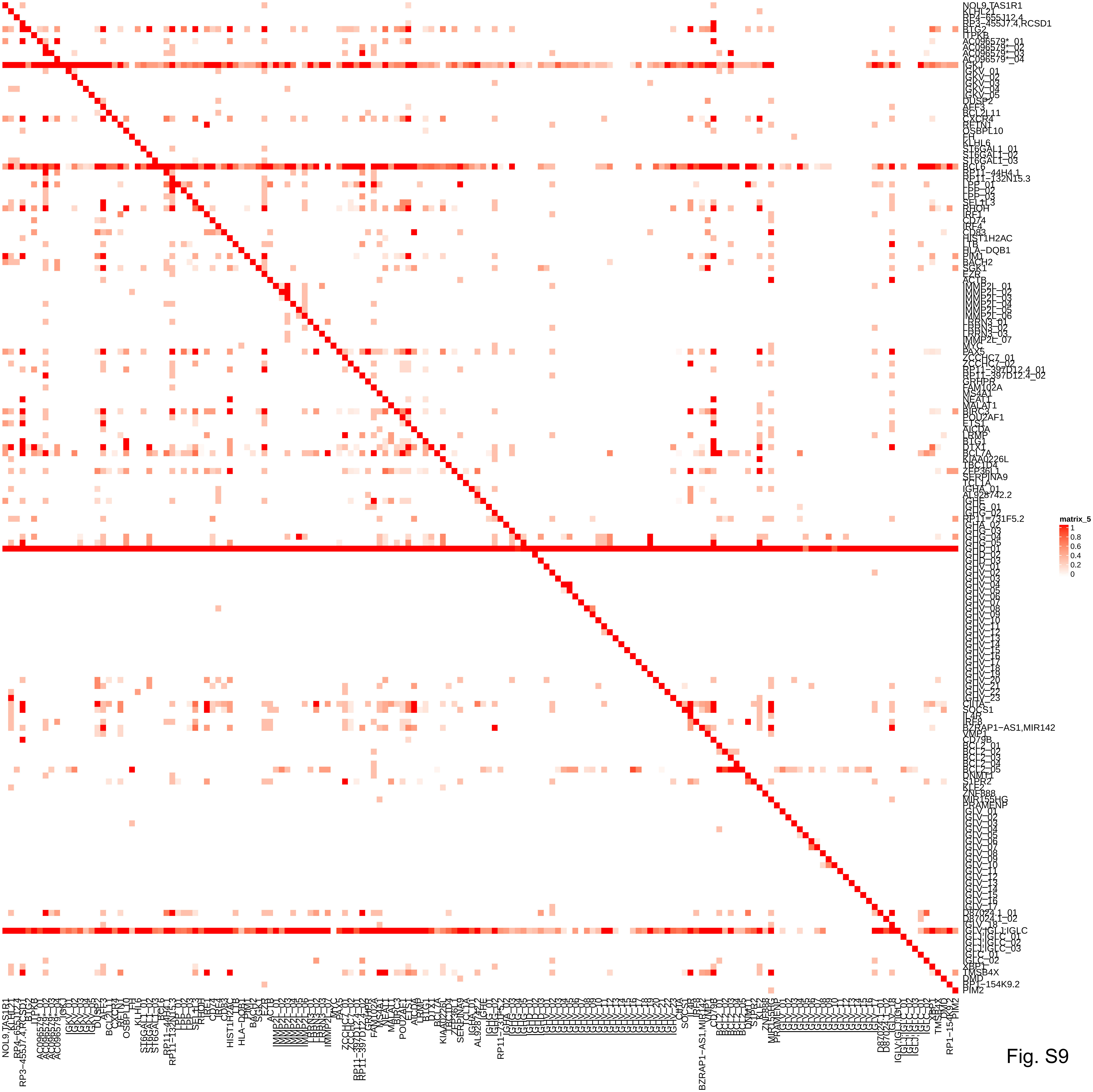

Fig. S9

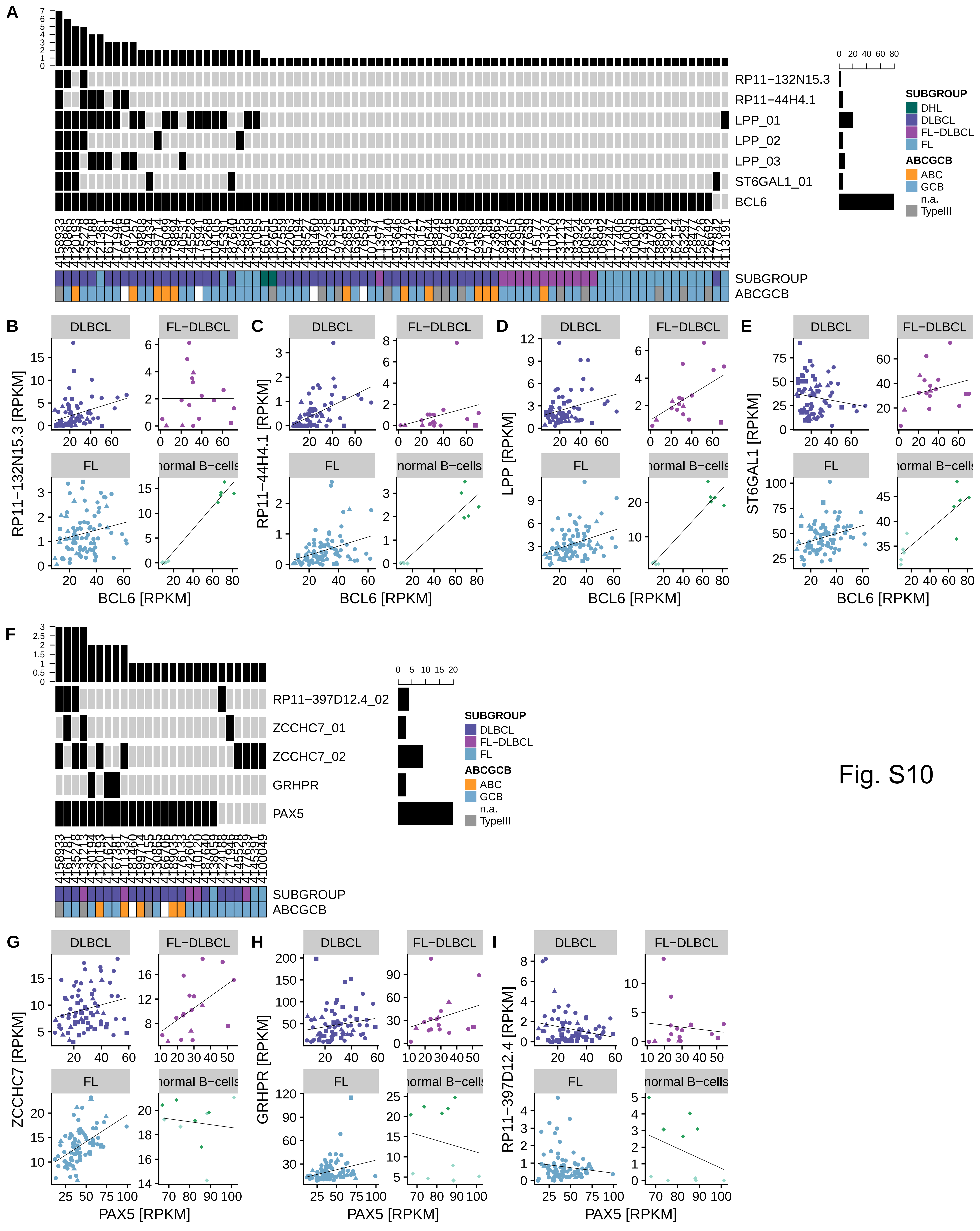

A
RP11−132N15.3
RP11−44H4.1
LPP_01
LPP_02
LPP_03
ST6GAL1_01
BCL6
SUBGROUP
DHL
DLBCL
FL−DLBCL
FL
ABCGCB
ABC
GCB
n.a.
TypeIII
B
C
D
E
DLBCL
FL−DLBCL
FL
normal B−cells
RP11−132N15.3 [RPKM]
RP11−44H4.1 [RPKM]
LPP [RPKM]
ST6GAL1 [RPKM]
BCL6 [RPKM]
F
RP11−397D12.4_02
ZCCHC7_01
ZCCHC7_02
GRHPR
PAX5
G
H
I
ZCCHC7 [RPKM]
GRHPR [RPKM]
RP11−397D12.4 [RPKM]
PAX5 [RPKM]

Fig. S10

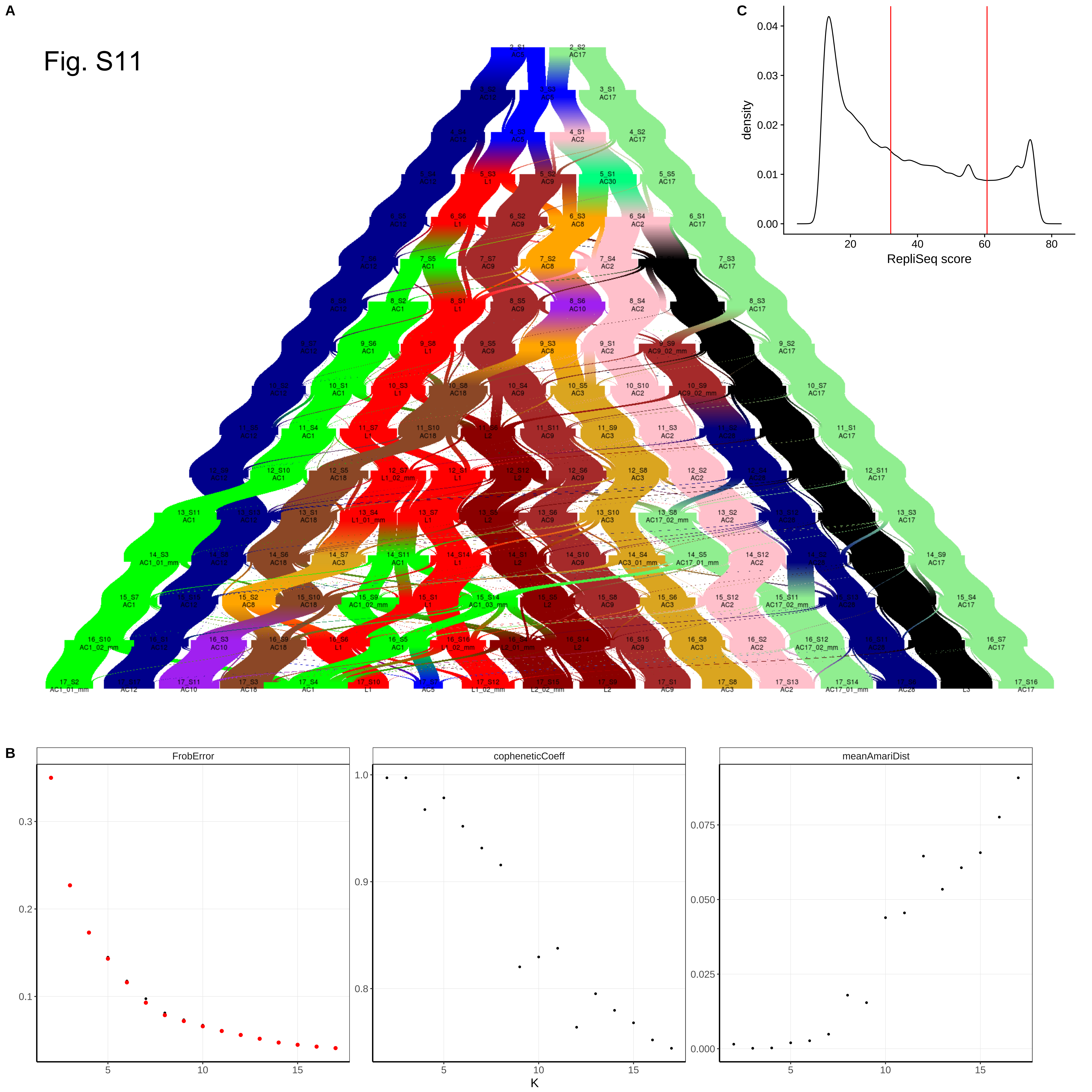

A
Fig. S11
2_S1 AC5
2_S2 AC17
3_S2 AC12
3_S3 AC5
3_S1 AC17
4_S4 AC12
4_S3 AC5
4_S1 AC2
4_S2 AC17
5_S4 AC12
5_S3 L1
5_S2 AC9
5_S1 AC30
5_S5 AC17
6_S5 AC12
6_S6 L1
6_S2 AC9
6_S3 AC8
6_S4 AC2
6_S1 AC17
7_S6 AC12
7_S5 AC1
7_S7 AC9
7_S2 AC8
7_S4 AC2
7_S3 AC17
8_S8 AC12
8_S2 AC1
8_S1 L1
8_S5 AC9
8_S6 AC10
8_S4 AC2
8_S3 AC17
9_S7 AC12
9_S6 AC1
9_S8 L1
9_S5 AC9
9_S3 AC8
9_S1 AC2
9_S9 AC9_02_mm
9_S2 AC17
10_S2 AC12
10_S1 AC1
10_S3 L1
10_S8 AC18
10_S4 AC9
10_S5 AC3
10_S10 AC2
10_S9 AC9_02_mm
10_S7 AC17
11_S5 AC12
11_S4 AC1
11_S7 L1
11_S10 AC18
11_S6 L2
11_S11 AC9
11_S9 AC3
11_S3 AC2
11_S2 AC28
11_S1 AC17
12_S9 AC12
12_S10 AC1
12_S5 AC18
12_S7 L1_02_mm
12_S1 L1
12_S12 L2
12_S6 AC9
12_S8 AC3
12_S2 AC2
12_S4 AC28
12_S11 AC17
13_S11 AC1
13_S13 AC12
13_S1 AC18
13_S4 L1_01_mm
13_S7 L1
13_S5 L2
13_S6 AC9
13_S10 AC3
13_S8 AC17_02_mm
13_S2 AC2
13_S12 AC28
13_S3 AC17
14_S3 AC1_01_mm
14_S8 AC12
14_S6 AC18
14_S7 AC3
14_S11 AC1
14_S14 L1
14_S1 L2
14_S10 AC9
14_S4 AC3_01_mm
14_S5 AC17_01_mm
14_S12 AC2
14_S2 AC28
14_S9 AC17
15_S7 AC1
15_S13 AC12
15_S2 AC8
15_S10 AC18
15_S9 AC1_02_mm
15_S1 L1
15_S14 AC1_03_mm
15_S5 L2
15_S8 AC9
15_S6 AC3
15_S12 AC2
15_S11 AC17_02_mm
15_S3 AC28
15_S4 AC17
16_S10 AC1_02_mm
16_S1 AC12
16_S3 AC10
16_S9 AC18
16_S6 L1
16_S5 AC1
16_S16 L1_02_mm
16_S4 L2_01_mm
16_S14 L2
16_S15 AC9
16_S8 AC3
16_S2 AC2
16_S12 AC17_02_mm
16_S11 AC28
16_S7 AC17
17_S2 AC1_01_mm
17_S17 AC12
17_S11 AC10
17_S3 AC18
17_S4 AC1
17_S10 L1
17_S7 AC5
17_S12 L1_02_mm
17_S15 L2_02_mm
17_S9 L2
17_S1 AC9
17_S8 AC3
17_S13 AC2
17_S14 AC17_01_mm
AC28
L3
17_S16 AC17
B
FrobError
copheneticCoeff
meanAmariDist
0.3
0.2
0.1
1.0
0.9
0.8
0.075
0.050
0.025
0.000
5
10
15
K
C
density
0.04
0.03
0.02
0.01
0.00
20
40
60
80
RepliSeq score

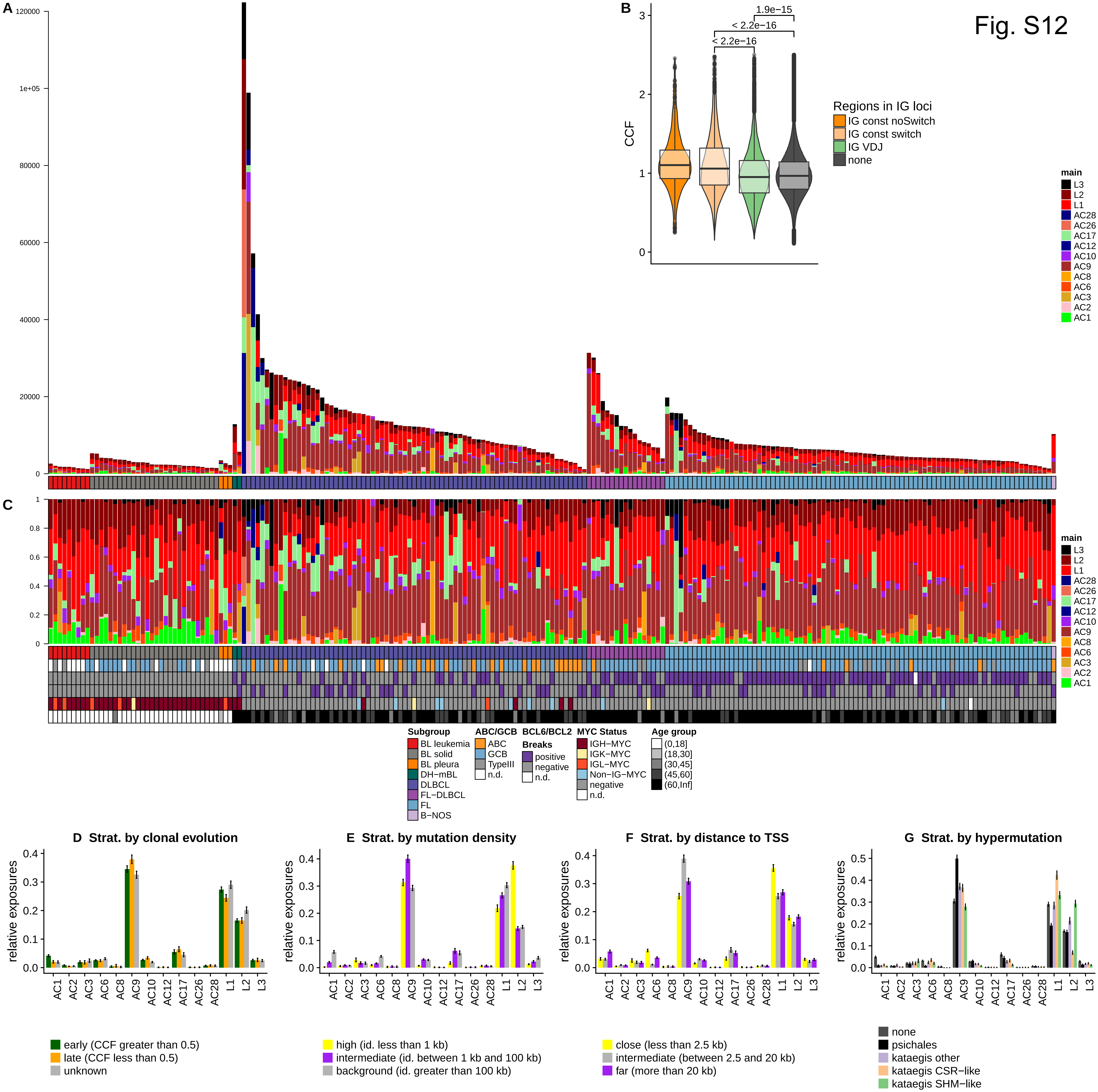
A
B
C
CCF
1.9e−15
< 2.2e−16
< 2.2e−16
Regions in IG loci
IG const noSwitch
IG const switch
IG VDJ
none
main
L3
L2
L1
AC28
AC26
AC17
AC12
AC10
AC9
AC8
AC6
AC3
AC2
AC1
Subgroup
BL leukemia
BL solid
BL pleura
DH−mBL
DLBCL
FL−DLBCL
FL
B−NOS
ABC/GCB
ABC
GCB
TypeIII
n.d.
BCL6/BCL2 Breaks
positive
negative
n.d.
MYC Status
IGH−MYC
IGK−MYC
IGL−MYC
Non−IG−MYC
negative
n.d.
Age group
(0,18]
(18,30]
(30,45]
(45,60]
(60,Inf]
D Strat. by clonal evolution
E Strat. by mutation density
F Strat. by distance to TSS
G Strat. by hypermutation
relative exposures
early (CCF greater than 0.5)
late (CCF less than 0.5)
unknown
high (id. less than 1 kb)
intermediate (id. between 1 kb and 100 kb)
background (id. greater than 100 kb)
close (less than 2.5 kb)
intermediate (between 2.5 and 20 kb)
far (more than 20 kb)
none
psichales
kataegis other
kataegis CSR−like
kataegis SHM−like

Fig. S12

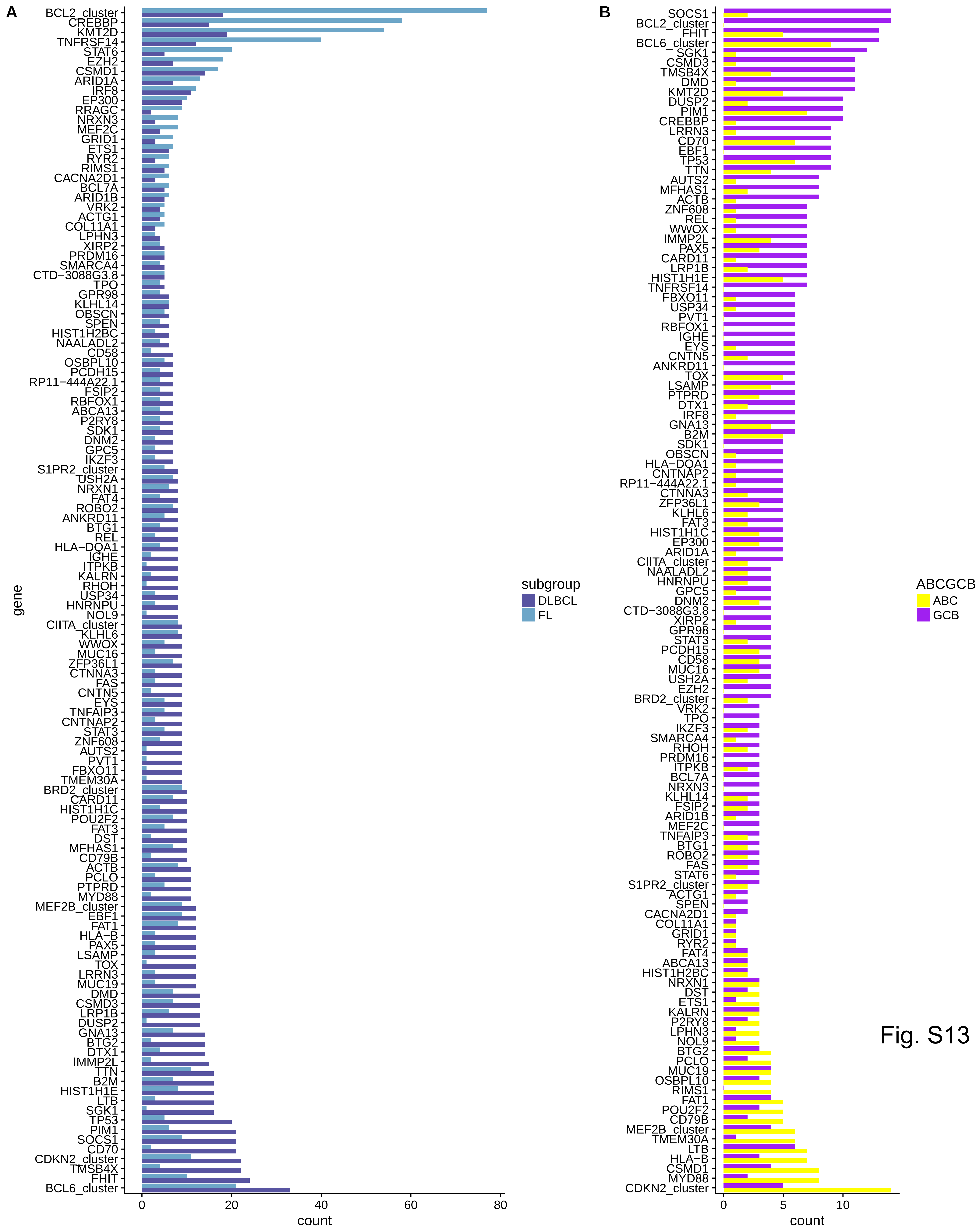
A
B
gene
count
subgroup
DLBCL
FL
ABCGCB
ABC
GCB
0
20
40
60
80
5
10
Fig. S13

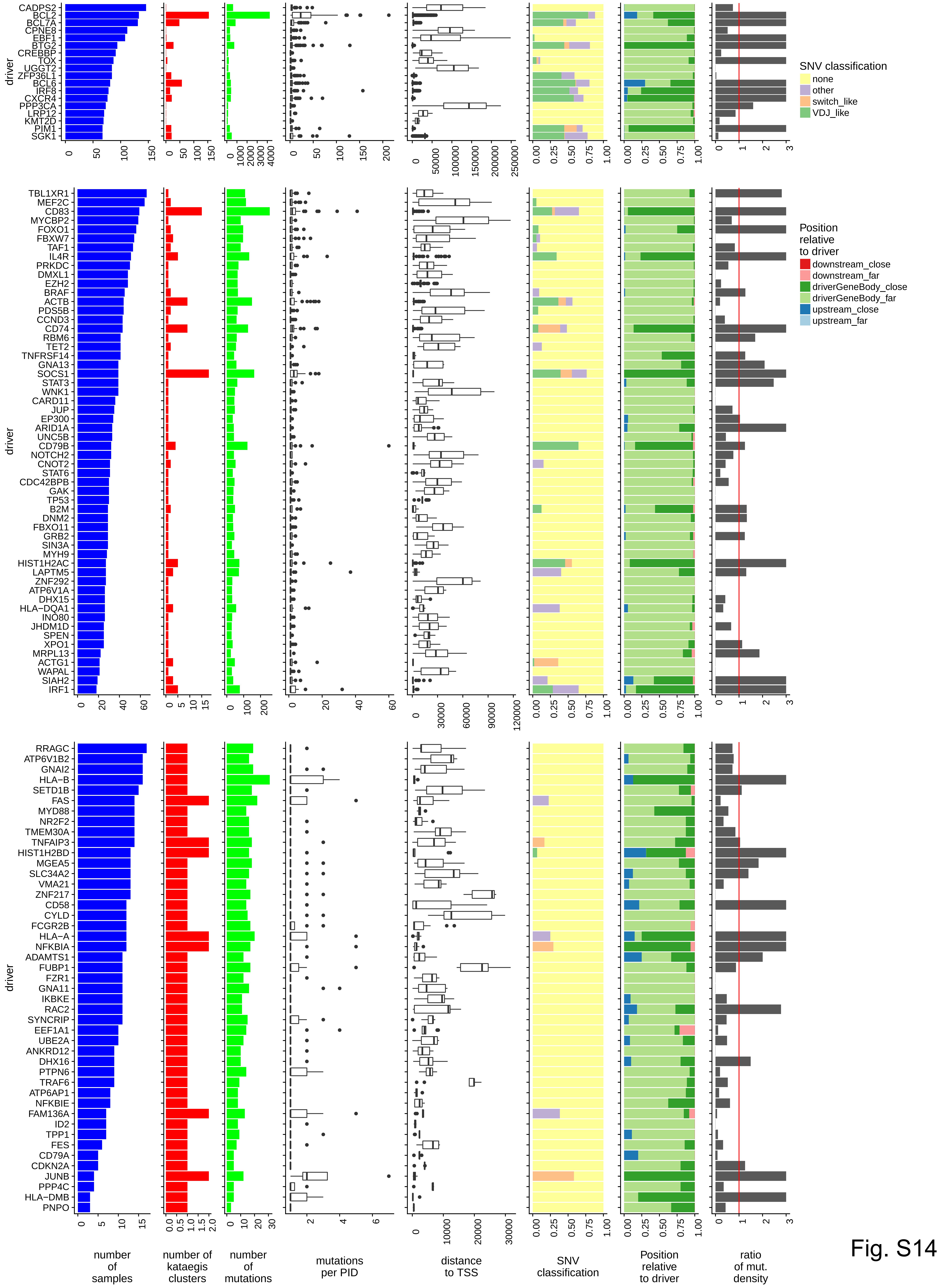

driver
CADPS2
BCL2
BCL7A
CPNE8
EBF1
BTG2
CREBBP
TOX
UGGT2
ZFP36L1
BCL6
IRF8
CXCR4
PPP3CA
LRP12
KMT2D
PIM1
SGK1
SNV classification
none
other
switch_like
VDJ_like
TBL1XR1
MEF2C
CD83
MYCBP2
FOXO1
FBXW7
TAF1
IL4R
PRKDC
DMXL1
EZH2
BRAF
ACTB
PDS5B
CCND3
CD74
RBM6
TET2
TNFRSF14
GNA13
SOCS1
STAT3
WNK1
CARD11
JUP
EP300
ARID1A
UNC5B
CD79B
NOTCH2
CNOT2
STAT6
CDC42BPB
GAK
TP53
B2M
DNM2
FBXO11
GRB2
SIN3A
MYH9
HIST1H2AC
LAPTM5
ZNF292
ATP6V1A
DHX15
HLA−DQA1
INO80
JHDM1D
SPEN
XPO1
MRPL13
ACTG1
WAPAL
SIAH2
IRF1
Position relative to driver
downstream_close
downstream_far
driverGeneBody_close
driverGeneBody_far
upstream_close
upstream_far
RRAGC
ATP6V1B2
GNAI2
HLA−B
SETD1B
FAS
MYD88
NR2F2
TMEM30A
TNFAIP3
HIST1H2BD
MGEA5
SLC34A2
VMA21
ZNF217
CD58
CYLD
FCGR2B
HLA−A
NFKBIA
ADAMTS1
FUBP1
FZR1
GNA11
IKBKE
RAC2
SYNCRIP
EEF1A1
UBE2A
ANKRD12
DHX16
PTPN6
TRAF6
ATP6AP1
NFKBIE
FAM136A
ID2
TPP1
FES
CD79A
CDKN2A
JUNB
PPP4C
HLA−DMB
PNPO
number of samples
number of kataegis clusters
number of mutations
mutations per PID
distance to TSS
SNV classification
Position relative to driver
ratio of mut. density

Fig. S14

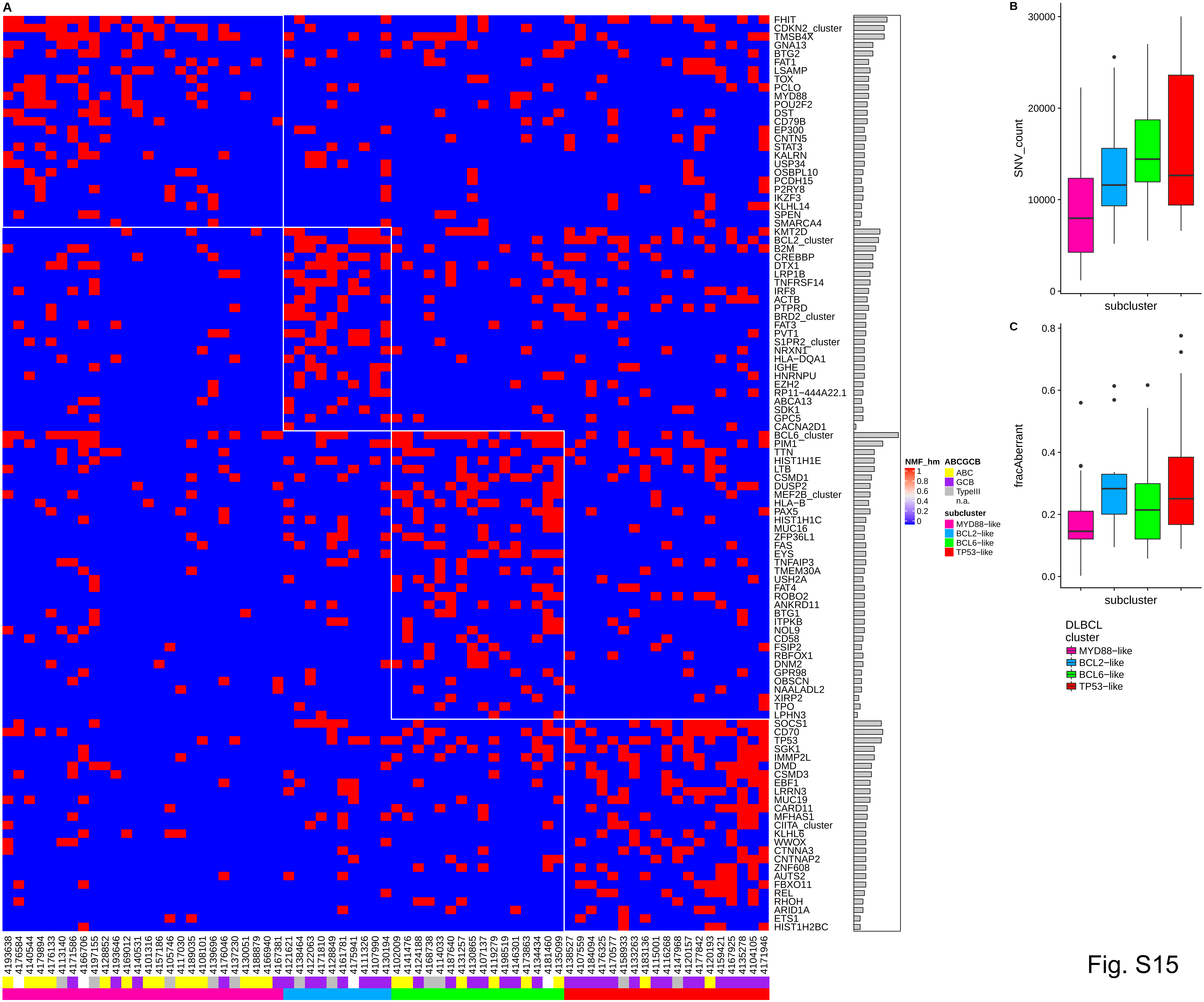

A
FHIT
CDKN2_cluster
TMSB4X
GNA13
BTG2
FAT1
LSAMP
TOX
PCLO
MYD88
POU2F2
DST
CD79B
EP300
CNTN5
STAT3
KALRN
USP34
OSBPL10
PCDH15
P2RY8
IKZF3
KLHL14
SPEN
SMARCA4
KMT2D
BCL2_cluster
B2M
CREBBP
DTX1
LRP1B
TNFRSF14
IRF8
ACTB
PTPRD
BRD2_cluster
FAT3
PVT1
S1PR2_cluster
NRXN1
HLA−DQA1
IGHE
HNRNPU
EZH2
RP11−444A22.1
ABCA13
SDK1
GPC5
CACNA2D1
BCL6_cluster
PIM1
TTN
HIST1H1E
LTB
CSMD1
DUSP2
MEF2B_cluster
HLA−B
PAX5
HIST1H1C
MUC16
ZFP36L1
FAS
EYS
TNFAIP3
TMEM30A
USH2A
FAT4
ROBO2
ANKRD11
BTG1
ITPKB
NOL9
CD58
FSIP2
RBFOX1
DNM2
GPR98
OBSCN
NAALADL2
XIRP2
TPO
LPHN3
SOCS1
CD70
TP53
SGK1
IMMP2L
DMD
CSMD3
EBF1
LRRN3
MUC19
CARD11
MFHAS1
CIITA_cluster
KLHL6
WWOX
CTNNA3
CNTNAP2
ZNF608
AUTS2
FBXO11
REL
RHOH
ARID1A
ETS1
HIST1H2BC
NMF_hm
1
0.8
0.6
0.4
0.2
0
ABCGCB
ABC
GCB
TypeIII
n.a.
subcluster
MYD88−like
BCL2−like
BCL6−like
TP53−like
B
SNV_count
30000
20000
10000
0
subcluster
C
fracAberrant
0.8
0.6
0.4
0.2
0.0
subcluster
DLBCL
cluster
MYD88−like
BCL2−like
BCL6−like
TP53−like

Fig. S15

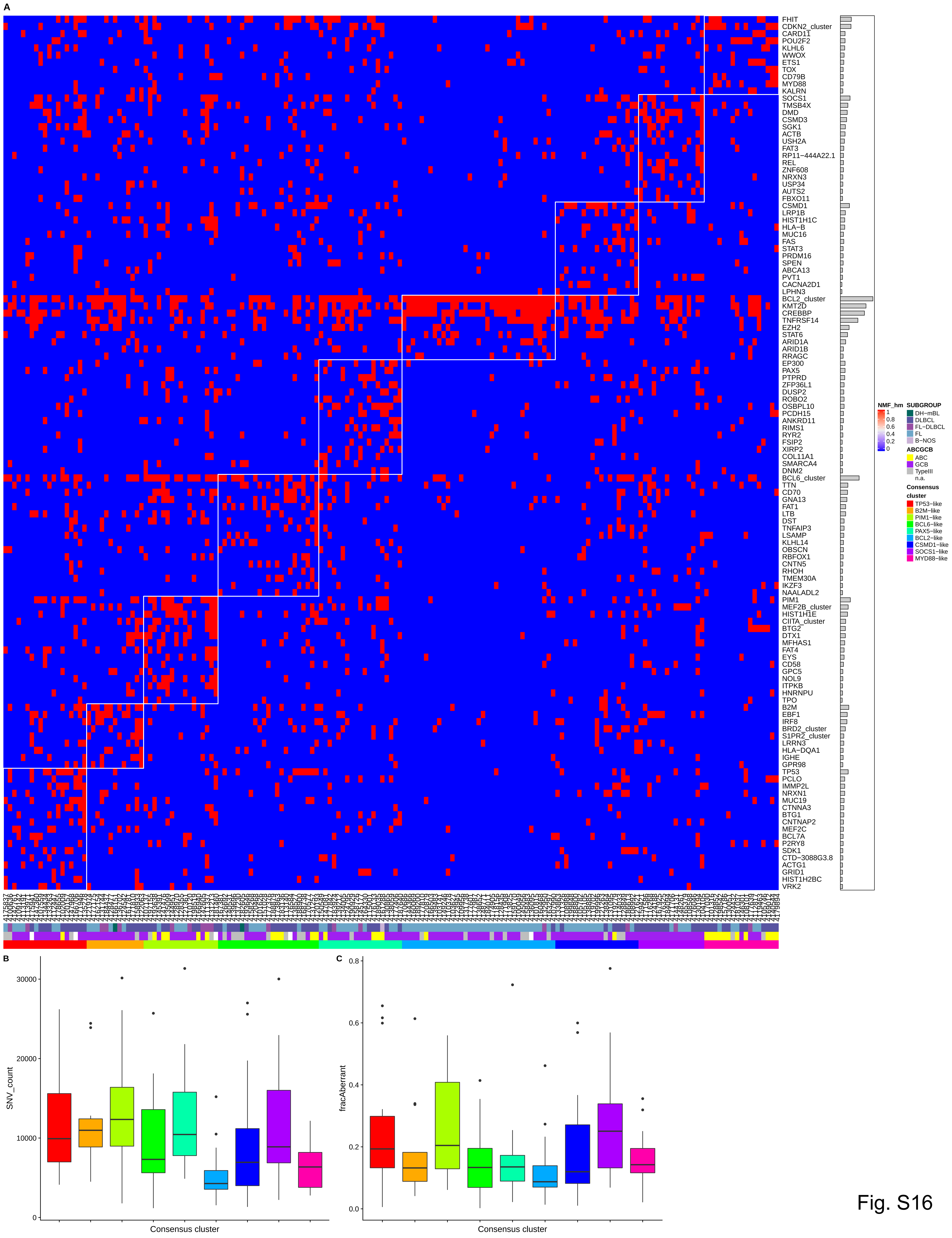
A
FHIT
CDKN2_cluster
CARD11
POU2F2
KLHL6
WWOX
ETS1
TOX
CD79B
MYD88
KALRN
SOCS1
TMSB4X
DMD
CSMD3
SGK1
ACTB
USH2A
FAT3
RP11−444A22.1
REL
ZNF608
NRXN3
USP34
AUTS2
FBXO11
CSMD1
LRP1B
HIST1H1C
HLA−B
MUC16
FAS
STAT3
PRDM16
SPEN
ABCA13
PVT1
CACNA2D1
LPHN3
BCL2_cluster
KMT2D
CREBBP
TNFRSF14
EZH2
STAT6
ARID1A
ARID1B
RRAGC
EP300
PAX5
PTPRD
ZFP36L1
DUSP2
ROBO2
OSBPL10
PCDH15
ANKRD11
RIMS1
RYR2
FSIP2
XIRP2
COL11A1
SMARCA4
DNM2
BCL6_cluster
TTN
CD70
GNA13
FAT1
LTB
DST
TNFAIP3
LSAMP
KLHL14
OBSCN
RBFOX1
CNTN5
RHOH
TMEM30A
IKZF3
NAALADL2
PIM1
MEF2B_cluster
HIST1H1E
CIITA_cluster
BTG2
DTX1
MFHAS1
FAT4
EYS
CD58
GPC5
NOL9
ITPKB
HNRNPU
TPO
B2M
EBF1
IRF8
BRD2_cluster
S1PR2_cluster
LRRN3
HLA−DQA1
IGHE
GPR98
TP53
PCLO
IMMP2L
NRXN1
MUC19
CTNNA3
BTG1
CNTNAP2
MEF2C
BCL7A
P2RY8
SDK1
CTD−3088G3.8
ACTG1
GRID1
HIST1H2BC
VRK2
NMF_hm
1
0.8
0.6
0.4
0.2
0
SUBGROUP
DH−mBL
DLBCL
FL−DLBCL
FL
B−NOS
ABCGCB
ABC
GCB
TypeIII
n.a.
Consensus cluster
TP53−like
B2M−like
PIM1−like
BCL6−like
PAX5−like
BCL2−like
CSMD1−like
SOCS1−like
MYD88−like
B
SNV_count
30000
20000
10000
0
Consensus cluster
C
fracAberrant
0.8
0.6
0.4
0.2
0.0
Consensus cluster

Fig. S16

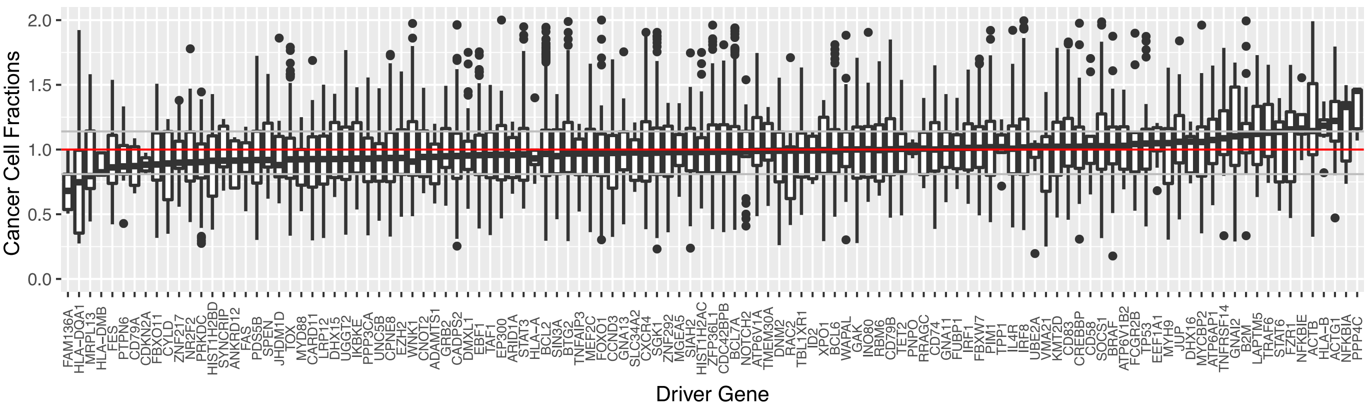

Cancer Cell Fractions
2.0
1.5
1.0
0.5
0.0
FAM136A
HLA−DQA1
MRPL13
HLA−DMB
FES
PTPN6
CD79A
CDKN2A
FBXO11
CYLD
ZNF217
NR2F2
PRKDC
HIST1H2BD
SYNCRIP
ANKRD12
FAS
PDS5B
SPEN
JHDM1D
TOX
MYD88
CARD11
LRP12
DHX15
UGGT2
IKBKE
PPP3CA
UNC5B
CPNE8
EZH2
WNK1
CNOT2
ADAMTS1
GRB2
CADPS2
DMXL1
EBF1
TAF1
EP300
ARID1A
STAT3
HLA−A
BCL2
SIN3A
BTG2
TNFAIP3
MEF2C
FOXO1
CCND3
GNA13
SLC34A2
CXCR4
SGK1
ZNF292
MGEA5
SIAH2
HIST1H2AC
ZFP36L1
CDC42BPB
BCL7A
NOTCH2
ATP6V1A
TMEM30A
DNM2
RAC2
TBL1XR1
ID2
XPO1
BCL6
WAPAL
GAK
INO80
RBM6
CD79B
TET2
PNPO
RRAGC
CD74
GNA11
FUBP1
IRF1
FBXW7
PIM1
TPP1
IL4R
IRF8
UBE2A
VMA21
KMT2D
CD83
CREBBP
CD58
SOCS1
BRAF
ATP6V1B2
FCGR2B
TP53
EEF1A1
MYH9
JUP
DHX16
MYCBP2
ATP6AP1
TNFRSF14
GNAI2
B2M
LAPTM5
TRAF6
STAT6
FZR1
NFKBIE
ACTB
HLA−B
ACTG1
NFKBIA
PPP4C
Driver Gene

Fig. S17

Fig. S18

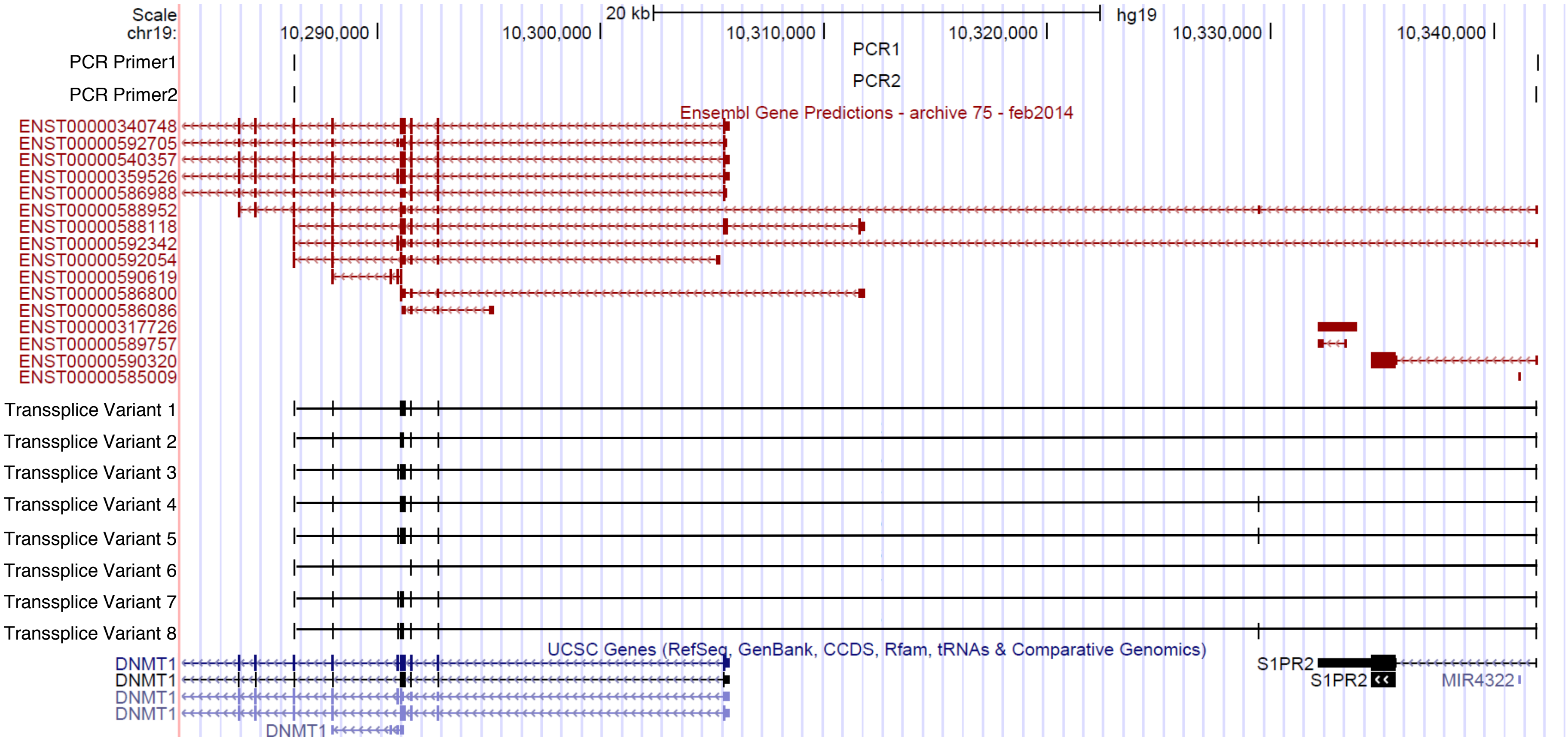

Scale
chr19:
20 kb
hg19
10,290,000
10,300,000
10,310,000
10,320,000
10,330,000
10,340,000
PCR Primer1
PCR Primer2
PCR1
PCR2
Ensembl Gene Predictions - archive 75 - feb2014
ENST00000340748
ENST00000592705
ENST00000540357
ENST00000359526
ENST00000586988
ENST00000588952
ENST00000588118
ENST00000592342
ENST00000592054
ENST00000590619
ENST00000586800
ENST00000586086
ENST00000317726
ENST00000589757
ENST00000590320
ENST00000585009
Transsplice Variant 1
Transsplice Variant 2
Transsplice Variant 3
Transsplice Variant 4
Transsplice Variant 5
Transsplice Variant 6
Transsplice Variant 7
Transsplice Variant 8
UCSC Genes (RefSeq, GenBank, CCDS, Rfam, tRNAs & Comparative Genomics)
DNMT1
DNMT1
DNMT1
DNMT1
DNMT1
S1PR2
S1PR2
MIR4322
