# Supplementary material for: Mutational mechanisms shaping the coding and noncoding genome of germinal center derived B-cell lymphomas
Source: Leukemia. 2021 May 5;35(7):2002–16. doi: 10.1038/s41375-021-01251-z (PMC8257491; doi:10.1038/s41375-021-01251-z)
Supplement: Supplementary file 4 — Supplementary Tables 1–5, 7, 9–13 [file 41375_2021_1251_MOESM4_ESM.pdf]

Supplementary Table 1A. Clinico-biological characteristics

ICGC-case	Age at diagnosis (years)	Sex	Tumor cell content based on WGS	RNA-available	molecular Burkitt index based classification (RNA-seq based gene expression classifier)	Diagnosis	Cell of origin (COO) based on RNAseq expression	Presence of chromosoml breakpoints / translocatios based on FISH/WGS/RNA-Seq				IHC analysis							in PCAWG
								MYC status	BCL2 status	BCL6 status	IGH Status	CD20 expression	CD10 expression	CD5 expression	BCL2 expression	BCL6 expression	MUM1 expression	KI67 expression	
4105105	40	f	0,77	yes	non-BL	FL	GCB	negative	positive	negative	positive	4	4	n.a.	4	3	0	20%	yes
4108101	66	m	0,51	yes	non-BL	DLBCL	ABC	negative	negative	negative	negative	4	0	n.a.	4	1	4	80%	yes
4121361	74	m	0,37	yes	non-BL	FL	GCB	negative	positive	negative	positive	4	4	n.a.	4	5	0	30%	yes
4134005	67	m	0,79	yes	non-BL	FL	GCB	negative	positive	negative	positive	4	4	n.a.	4	n.a.	n.a.	20%	yes
4135099	49	m	0,8	yes	non-BL	DLBCL	ABC	negative	negative	positive	positive	4	0	n.a.	4	4	4	90%	yes
4163639	75	f	0,6	yes	non-BL	DLBCL	GCB	negative	negative	negative	negative	4	4	n.a.	4	1	5	20%	yes
4101316	74	f	0,3	yes	non-BL	DLBCL	ABC	negative	negative	negative	negative	4	0	n.a.	4	4	4	70%	no
4158726	48	m	0,52	yes	non-BL	FL	GCB	negative	positive	negative	positive	4	4	n.a.	3	4	2	50%	yes
4159170	43	m	0,58	yes	non-BL	FL	GCB	negative	positive	negative	positive	4	4	n.a.	4	n.a.	1	30%	yes
4175837	74	f	0,34	yes	non-BL	FL	TypeIII	negative	positive	positive	positive	4	4	n.a.	4	5	1	n.a.	yes
4177376	73	f	0,59	yes	non-BL	FL-DLBCL	TypeIII	negative	positive	negative	positive	4	0	n.a.	4	n.a.	n.a.	60%	yes
4188900	76	m	0,52	yes	non-BL	FL	TypeIII	negative	positive	negative	negative	4	4	n.a.	4	2	1	30%	yes
4189200	51	f	0,66	yes	non-BL	FL	GCB	negative	positive	negative	positive	4	3	n.a.	4	n.a.	n.a.	10%	yes
4131257	72	m	0,5	yes	non-BL	DLBCL	ABC	negative	negative	positive	positive	4	0	n.a.	4	1	2	80%	yes
4131744	68	m	0,52	yes	non-BL	FL-DLBCL	GCB	negative	positive	negative	positive	4	4	n.a.	4	3	1	80%	yes
4160468	62	m	0,62	yes	non-BL	FL	GCB	negative	positive	negative	positive	4	4	n.a.	4	4	0	30%	yes
4171706	70	f	0,61	yes	non-BL	FL	GCB	negative	positive	negative	positive	4	4	n.a.	4	3	1	10%	yes
4174905	72	m	0,57	yes	non-BL	FL	TypeIII	negative	positive	negative	positive	4	4	n.a.	4	4	0	10%	yes
4177987	71	m	0,4	yes	non-BL	FL	GCB	negative	positive	negative	positive	4	3	n.a.	4	4	1	20%	yes
4102009	64	m	0,71	yes	non-BL	DLBCL	ABC	negative	negative	negative	negative	4	0	n.a.	4	3	4	90%	no
4107137	59	m	0,41	yes	non-BL	DLBCL	GCB	negative	negative	positive	positive	4	0	n.a.	4	3	4	70%	no

4120193	41	f	0,47	yes	non-BL	DLBCL	ABC	negative	negative	negative	negative	4	0	n.a.	4	1	3	70%	yes
4139696	70	m	0,46	yes	non-BL	DLBCL	TypeIII	negative	negative	positive	positive	4	4	0	0	4	0	90%	yes
4166706	62	m	0,53	no	NA	DLBCL	n.a.	negative	negative	positive	positive	4	0	n.a.	1	4	4	90%	yes
4170686	56	m	0,55	yes	non-BL	FL	GCB	negative	positive	negative	positive	4	0	n.a.	4	3	0	10%	yes
4176133	61	f	0,77	yes	non-BL	DLBCL	ABC	negative	negative	positive	positive	4	0	n.a.	4	4	0	50%	yes
4177601	52	f	0,3	yes	non-BL	FL	GCB	negative	positive	negative	positive	4	4	n.a.	4	4	1	30%	yes
4184094	57	f	0,79	yes	non-BL	DLBCL	GCB	negative	positive	negative	positive	4	4	n.a.	4	4	1	80%	yes
4112447	46	m	0,63	yes	non-BL	FL	GCB	negative	positive	positive	positive	4	4	n.a.	2	3	2	10%	no
4113825	74	f	0,64	yes	non-BL	FL	GCB	negative	positive	negative	positive	4	2	n.a.	0	2	1	40%	no
4115001	70	f	0,45	yes	non-BL	DLBCL	GCB	negative	positive	negative	positive	4	0	4	4	3	0	10%	no
4119279	62	f	0,84	yes	non-BL	DLBCL	ABC	negative	negative	negative	negative	4	0	0	1	1	4	90%	no
4120157	46	m	0,61	yes	non-BL	DLBCL	GCB	negative	negative	negative	positive	4	0	n.a.	2	4	0	70%	no
4133263	40	m	0,68	yes	intermediate	DLBCL	GCB	non-IG-MYC pos	negative	negative	positive	4	4	0	1	4	0	90%	yes
4134434	84	m	0,86	yes	non-BL	DLBCL	GCB	negative	positive	positive	positive	4	0	n.a.	3	4	4	90%	yes
4142605	76	f	0,79	yes	non-BL	FL-DLBCL	GCB	negative	positive	negative	positive	4	5	n.a.	4	4	1	30%	yes
4145056	67	f	0,69	yes	non-BL	FL	TypeIII	negative	positive	negative	positive	4	4	n.a.	2	4	4	100%	yes
4145177	75	f	0,8	yes	non-BL	FL-DLBCL	GCB	negative	positive	negative	positive	4	4	0	4	4	0	50%	yes
4149246	41	m	0,68	yes	non-BL	FL	GCB	negative	positive	positive	positive	4	4	0	4	3	2	10%	yes
4157186	74	m	0,75	yes	non-BL	DLBCL	ABC	negative	negative	negative	negative	4	0	0	4	2	3	70%	yes
4159421	78	f	0,34	yes	non-BL	DLBCL	GCB	negative	negative	negative	negative	4	4	n.a.	4	3	2	10%	yes
4177810	47	f	0,64	yes	non-BL	FL	TypeIII	negative	positive	negative	positive	4	4	n.a.	4	4	0	20%	yes
4178655	50	m	0,72	yes	non-BL	FL	GCB	negative	positive	negative	positive	4	4	0	4	3	0	30%	yes
4189035	46	m	0,49	yes	non-BL	DLBCL	ABC	negative	negative	negative	negative	4	0	0	4	1	3	90%	yes
4198542	68	f	0,3	yes	non-BL	FL	ABC	negative	negative	negative	negative	4	1	n.a.	2	3	3	40%	yes
4188879	52	m	0,62	yes	non-BL	DLBCL	ABC	non-IG-MYC pos	negative	negative	positive	4	0	n.a.	4	4	4	70%	yes
4166503	43	m	0,81	yes	non-BL	FL	GCB	negative	positive	negative	positive	4	4	n.a.	3	n.a.	n.a.	30%	yes
4110378	69	f	0,64	yes	non-BL	FL	GCB	negative	positive	negative	positive	4	0	n.a.	3	4	2	80%	no
4142761	63	m	0,8	yes	non-BL	FL	GCB	negative	positive	negative	positive	4	4	n.a.	4	3	1	20%	no
4109956	54	f	0,81	yes	non-BL	FL	GCB	negative	positive	positive	positive	4	4	n.a.	4	5	1	10%	no
4128477	54	m	0,57	yes	non-BL	FL	GCB	negative	positive	negative	positive	4	4	n.a.	4	3	1	30%	no

4184011	61	m	0,3	yes	non-BL	FL	GCB	negative	not-determinable	negative	not-determinable	4	4	n.a.	0	2	1	50%	yes
4197155	70	f	0,4	yes	non-BL	DLBCL	TypeIII	negative	negative	positive	positive	3	0	n.a.	3	4	4	70%	yes
4138885	55	f	0,3	yes	non-BL	FL	GCB	negative	negative	negative	negative	4	0	0	0	1	0	60%	no
4181460	70	f	0,44	no	NA	DLBCL	n.a.	negative	negative	positive	positive	4	0	0	4	4	3	90%	yes
4136702	75	f	0,82	yes	non-BL	FL-DLBCL	GCB	negative	positive	negative	positive	4	4	n.a.	4	4	1	50%	no
4110120	68	m	0,52	yes	non-BL	FL-DLBCL	GCB	negative	negative	positive	positive	4	0	0	4	2	4	70%	no
4177406	72	f	0,58	yes	non-BL	FL	GCB	negative	positive	negative	positive	4	4	n.a.	4	3	0	20%	yes
4187640	58	m	0,72	yes	non-BL	DLBCL	GCB	negative	negative	positive	positive	4	0	0	2	4	1	90%	yes
4124188	73	m	0,76	yes	non-BL	DLBCL	GCB	negative	negative	negative	negative	4	4	n.a.	4	4	1	80%	no
4130194	55	f	0,85	yes	non-BL	DLBCL	GCB	non-IG-MYC pos	positive	negative	positive	4	4	n.a.	3	4	4	80%	no
4138527	85	f	0,72	yes	non-BL	DLBCL	GCB	negative	positive	negative	positive	4	0	n.a.	4	4	0	90%	no
4147968	65	f	0,67	yes	non-BL	DLBCL	TypeIII	negative	positive	negative	positive	4	4	n.a.	4	2	4	90%	no
4103141	60	f	0,3	yes	non-BL	FL	GCB	non-IG-MYC pos	positive	positive	positive	4	4	0	4	4	0	10%	no
4108992	68	m	0,77	yes	non-BL	FL	GCB	negative	positive	positive	positive	4	4	0	4	4	1	10%	no
4111326	74	f	0,72	yes	non-BL	DLBCL	GCB	non-IG-MYC pos	positive	negative	positive	4	4	0	4	4	0	80%	no
4111337	79	f	0,61	yes	non-BL	FL-DLBCL	ABC	negative	negative	positive	positive	4	0	n.a.	4	4	4	90%	no
4113191	62	f	0,65	yes	non-BL	FL	GCB	negative	positive	negative	positive	4	4	n.a.	4	4	0	10%	no
4124542	72	f	0,33	yes	non-BL	FL	GCB	negative	positive	negative	positive	4	4	0	4	4	1	10%	no
4131095	56	f	0,35	yes	non-BL	FL	GCB	negative	negative	negative	positive	4	0	n.a.	0	4	0	60%	no
4144951	62	f	0,72	yes	non-BL	FL-DLBCL	GCB	negative	positive	negative	positive	4	4	n.a.	4	4	0	70%	no
4145528	62	m	0,49	yes	non-BL	DLBCL	GCB	negative	negative	negative	negative	4	0	n.a.	4	4	4	70%	no
4147081	42	f	0,79	yes	non-BL	FL	ABC	negative	positive	positive	positive	4	4	0	4	2	0	40%	no
4150549*	76	f	0,66	yes	non-BL	FL	GCB	negative	positive	positive	positive	4	4	n.a.	4	4	0	10%	no
4161781	36	f	0,43	yes	non-BL	DLBCL	GCB	negative	negative	negative	negative	4	0	0	1	1	1	30%	no
4165379	64	f	0,3	yes	non-BL	FL	GCB	negative	negative	negative	negative	4	4	n.a.	1	4	1	30%	no
4166151	74	f	0,84	yes	BL	DH-BL	GCB	non-IG-MYC pos	positive	negative	positive	4	4	n.a.	4	4	0	90%	no
4193638	55	f	0,76	yes	non-BL	DLBCL	ABC	negative	negative	positive	positive	4	0	n.a.	4	4	4	80%	yes
4199714	53	f	0,69	yes	non-BL	DLBCL	ABC	negative	negative	positive	negative	4	0	0	0	4	4	90%	yes
4199996	73	m	0,46	yes	non-BL	FL	GCB	negative	positive	negative	positive	4	4	n.a.	4	4	0	30%	yes
4107559	72	m	0,77	yes	intermediate	DLBCL	GCB	non-IG-MYC pos	positive	negative	positive	4	4	n.a.	4	4	1	90%	no

4113971	61	f	0,37	yes	non-BL	FL-DLBCL	GCB	negative	negative	positive	positive	4	0	n.a.	2	4	3	70%	no
4122063	47	m	0,54	yes	intermediate	DLBCL	GCB	non-IG-MYC pos	positive	negative	positive	4	4	n.a.	4	4	0	30%	no
4128852	57	f	0,57	yes	non-BL	DLBCL	ABC	negative	negative	negative	negative	4	4	0	4	4	4	80%	no
4140531	66	m	0,67	yes	non-BL	DLBCL	GCB	negative	negative	negative	negative	4	4	n.a.	5	4	3	100%	no
4158268	54	f	0,3	yes	non-BL	FL	GCB	negative	positive	negative	positive	4	4	0	4	4	0	20%	no
4198519	67	f	0,3	yes	non-BL	DLBCL	GCB	negative	negative	positive	positive	4	4	n.a.	0	3	1	80%	yes
4101626	68	m	0,6	yes	non-BL	FL	GCB	negative	positive	negative	positive	3	4	n.a.	4	3	1	20%	no
4104105	64	m	0,32	yes	non-BL	DLBCL	GCB	negative	negative	negative	negative	4	0	n.a.	4	4	2	80%	no
4105746	58	m	0,71	yes	non-BL	DLBCL	TypeIII	negative	negative	negative	negative	4	0	n.a.	4	1	2	90%	no
4108988	55	m	0,44	yes	non-BL	FL	GCB	negative	negative	negative	negative	4	4	n.a.	4	4	n.a.	10%	no
4109142	74	f	0,6	yes	non-BL	FL	GCB	negative	negative	positive	positive	4	4	n.a.	3	3	1	10%	no
4109808	63	m	0,79	yes	non-BL	DLBCL	GCB	negative	positive	negative	positive	4	4	n.a.	4	4	n.a.	30%	no
4117030	58	m	0,84	yes	non-BL	DLBCL	ABC	negative	negative	negative	negative	4	0	n.a.	4	2	1	60%	no
4120468	80	f	0,47	yes	non-BL	FL	GCB	negative	positive	positive	positive	4	4	0	4	4	0	10%	no
4128970	61	f	0,56	yes	non-BL	FL	GCB	negative	positive	positive	positive	4	4	n.a.	4	3	1	10%	no
4130051	74	f	0,8	yes	non-BL	DLBCL	ABC	non-IG-MYC pos	negative	negative	positive	4	0	n.a.	4	4	3	60%	no
4138464	47	f	0,9	yes	non-BL	DLBCL	TypeIII	IGL-MYC pos	positive	negative	positive	4	0	n.a.	1	3	n.a.	80%	no
4145391	73	m	0,86	yes	non-BL	FL	GCB	negative	positive	positive	positive	4	4	0	4	4	4	70%	no
4148771	64	f	0,69	yes	non-BL	FL	GCB	negative	positive	negative	positive	4	4	n.a.	4	3	0	30%	no
4158483	46	m	0,72	yes	non-BL	FL	TypeIII	negative	positive	negative	positive	4	4	n.a.	4	2	1	20%	no
4163297	47	m	0,61	yes	non-BL	FL	TypeIII	negative	positive	positive	positive	4	4	n.a.	2	5	1	30%	no
4168738	50	m	0,45	yes	non-BL	DLBCL	TypeIII	negative	negative	positive	positive	4	0	n.a.	3	4	1	90%	no
4171810	52	f	0,62	yes	non-BL	DLBCL	GCB	IGK-MYC pos	positive	negative	positive	4	4	n.a.	3	3	2	30%	no
4173863	53	f	0,36	yes	non-BL	DLBCL	ABC	negative	negative	positive	positive	4	4	n.a.	4	5	4	80%	no
4176325	80	f	0,63	yes	non-BL	DLBCL	GCB	negative	negative	negative	negative	4	4	n.a.	0	n.a.	n.a.	80%	no
4177175	74	f	0,72	yes	non-BL	FL	GCB	negative	positive	negative	positive	4	4	n.a.	4	4	0	80%	no
4179894	72	m	0,79	yes	non-BL	DLBCL	ABC	negative	negative	negative	not-determinable	4	4	0	0	n.a.	n.a.	90%	no
4181037	70	f	0,68	yes	non-BL	FL	ABC	negative	negative	negative	negative	4	0	0	4	4	n.a.	40%	no
4183136	55	m	0,54	yes	non-BL	DLBCL	ABC	negative	negative	negative	negative	4	2	0	1	2	n.a.	60%	no
4183924	72	f	0,76	yes	non-BL	FL-DLBCL	GCB	non-IG-MYC pos	positive	negative	positive	4	4	n.a.	4	4	0	60%	no

4188800	34	f	0,33	yes	non-BL	FL	GCB	negative	positive	negative	positive	4	4	n.a.	4	5	1	70%	no
4135278	43	f	0,3	yes	non-BL	DLBCL	GCB	negative	negative	negative	positive	4	0	n.a.	4	2	1	70%	no
4132950	76	m	0,5	yes	non-BL	FL-DLBCL	GCB	negative	positive	negative	positive	4	4	n.a.	4	4	0	60%	no
4101815	69	f	0,65	yes	non-BL	FL	GCB	negative	positive	negative	positive	4	4	n.a.	4	3	0	40%	no
4113140	43	m	0,41	yes	non-BL	DLBCL	TypeIII	negative	negative	positive	positive	4	0	n.a.	0	0	0	40%	no
4151028	67	f	0,56	yes	non-BL	FL	GCB	negative	positive	negative	positive	4	4	n.a.	4	3	0	n.a.	no
4138652	45	m	0,51	yes	non-BL	FL	GCB	negative	positive	negative	positive	4	4	0	4	4	0	10%	no
4141476	79	f	0,3	yes	non-BL	DLBCL	ABC	negative	negative	positive	positive	4	0	n.a.	4	4	n.a.	30%	no
4144366	40	m	0,73	yes	non-BL	FL	GCB	negative	positive	negative	positive	4	4	n.a.	4	3	1	20%	no
4199848	33	m	0,56	yes	non-BL	FL	GCB	negative	positive	negative	positive	4	4	n.a.	4	3	1	30%	no
4121263	64	f	0,41	yes	non-BL	FL	GCB	negative	negative	positive	positive	4	4	n.a.	0	4	0	10%	no
4144131	75	m	0,63	yes	non-BL	FL-DLBCL	GCB	IGK-MYC pos	positive	negative	positive	4	4	0	4	4	0	10%	no
4148261	61	f	0,33	yes	non-BL	FL	GCB	negative	positive	negative	positive	4	4	n.a.	4	3	0	10%	no
4108588	62	m	0,38	yes	non-BL	FL	GCB	negative	positive	positive	positive	4	4	n.a.	4	3	1	20%	no
4107990	45	f	0,59	yes	non-BL	DLBCL	GCB	negative	positive	negative	positive	4	4	0	4	4	0	90%	no
4100636	43	m	0,59	yes	non-BL	FL-DLBCL	TypeIII	negative	negative	positive	positive	4	n.a.	n.a.	4	2	0	10%	no
4119463	51	f	0,55	yes	non-BL	FL	GCB	non-determinable	positive	negative	positive	4	4	0	4	4	0	10%	no
4116268	52	m	0,52	yes	non-BL	DLBCL	GCB	negative	negative	positive	positive	4	0	n.a.	4	1	0	30%	no
4123945	57	f	0,76	yes	non-BL	FL	GCB	negative	positive	negative	positive	4	4	0	4	4	0	10%	no
4124795	75	f	0,49	yes	non-BL	FL	GCB	negative	positive	negative	positive	4	4	n.a.	4	4	2	10%	no
4131738	45	m	0,64	yes	non-BL	FL	TypeIII	negative	positive	negative	positive	4	4	n.a.	4	4	n.a.	10%	no
4158933	67	m	0,53	yes	non-BL	DLBCL	TypeIII	negative	positive	negative	positive	4	4	n.a.	4	3	1	80%	no
4133863	56	m	0,49	yes	non-BL	FL	GCB	negative	positive	negative	positive	4	4	0	4	2	0	10%	no
4160069	51	f	0,76	yes	non-BL	FL	GCB	negative	positive	negative	positive	4	4	n.a.	4	4	0	10%	no
4162154	51	f	0,56	yes	non-BL	FL	GCB	negative	positive	negative	positive	4	4	0	4	4	0	10%	no
4167381	68	m	NA	yes	non-BL	DLBCL	GCB	negative	negative	positive	positive	4	0	n.a.	0	1	0	80%	no
4166940	46	m	0,3	yes	non-BL	DLBCL	ABC	negative	negative	positive	positive	4	0	n.a.	4	0	n.a.	0%	no
4169012	60	f	0,32	yes	non-BL	DLBCL	ABC	negative	negative	negative	negative	4	4	0	4	4	4	80%	no
4177639	77	f	0,71	yes	non-BL	FL-DLBCL	GCB	negative	positive	positive	positive	4	4	0	4	2	0	50%	no
4170577	89	f	0,3	yes	non-BL	DLBCL	GCB	non-IG-MYC pos	positive	negative	positive	4	4	n.a.	4	3	0	40%	no

4193646	33	m	0,32	yes	non-BL	DLBCL	GCB	negative	negative	negative	negative	4	0	n.a.	4	4	2	80%	no
4105782	57	m	0,3	yes	non-BL	FL	GCB	negative	positive	negative	positive	4	0	n.a.	4	2	1	30%	no
4118156	71	f	0,55	yes	non-BL	FL	GCB	negative	positive	negative	positive	4	4	0	4	4	0	20%	no
4119702	74	m	0,64	yes	non-BL	FL	GCB	negative	positive	negative	positive	4	4	1	4	4	0	10%	no
4103627	51	f	0,3	yes	non-BL	FL	GCB	negative	positive	negative	positive	4	4	0	4	4	0	20%	no
4130865	54	m	0,2	yes	non-BL	DLBCL	GCB	negative	negative	positive	positive	4	4	n.a.	2	3	3	90%	no
4128435	53	f	0,42	yes	non-BL	FL	GCB	negative	positive	negative	positive	4	4	n.a.	4	4	0	20%	no
4128355	75	m	0,38	yes	non-BL	FL	GCB	negative	positive	positive	positive	4	4	n.a.	2	3	1	20%	no
4124432	64	f	0,62	yes	non-BL	FL	TypeIII	negative	positive	positive	positive	4	3	n.a.	4	3	0	10%	no
4121974	74	f	0,38	yes	non-BL	FL	GCB	negative	positive	negative	positive	4	3	n.a.	4	4	1	20%	no
4120879	53	m	0,65	yes	non-BL	FL	ABC	negative	positive	negative	positive	4	4	n.a.	4	3	2	10%	no
4114033	71	m	0,73	yes	non-BL	DLBCL	TypeIII	negative	negative	negative	negative	4	4	n.a.	0	3	n.a.	60%	no
4112817	49	m	0,75	yes	non-BL	FL	GCB	negative	positive	negative	negative	4	4	0	4	4	0	10%	no
4100049	76	m	0,65	yes	non-BL	FL	GCB	negative	positive	negative	positive	4	4	n.a.	4	3	0	10%	no
4121621	57	m	0,62	yes	non-BL	DLBCL	GCB	non-IG-MYC pos	negative	negative	negative	4	0	n.a.	4	4	4	80%	no
4128849	54	f	0,57	yes	non-BL	DLBCL	TypeIII	negative	negative	negative	negative	4	0	n.a.	1	1	1	50%	no
4139483	64	f	0,5	yes	non-BL	FL	GCB	negative	positive	positive	positive	4	4	n.a.	4	3	1	20%	no
4131213	71	f	0,34	yes	non-BL	FL-DLBCL	TypeIII	negative	negative	positive	positive	4	0	0	4	3	n.a.	40%	no
4136095	78	f	0,61	no	NA	FL	n.a.	negative	positive	negative	positive	4	4	n.a.	4	4	0	20%	no
4138059	73	f	0,51	yes	non-BL	FL	GCB	negative	positive	negative	negative	4	4	n.a.	4	3	0	n.a.	no
4184437	70	f	0,73	yes	non-BL	FL-DLBCL	GCB	negative	positive	negative	positive	4	0	n.a.	4	4	n.a.	40%	no
4126692	65	f	0,46	yes	non-BL	FL	TypeIII	negative	negative	positive	negative	4	0	0	2	1	1	10%	no
4139212	72	f	0,4	yes	non-BL	FL	GCB	negative	positive	negative	positive	4	4	n.a.	4	4	0	20%	no
4140544	74	f	0,58	yes	non-BL	DLBCL	ABC	negative	negative	negative	negative	4	0	4	4	0	4	50%	no
4146301	69	f	0,49	yes	non-BL	DLBCL	GCB	negative	negative	negative	negative	4	0	n.a.	0	0	0	90%	no
4147360	62	f	0,51	yes	non-BL	FL	GCB	negative	positive	negative	positive	4	4	0	4	4	0	10%	no
4137230	59	f	0,3	yes	non-BL	DLBCL	TypeIII	negative	negative	positive	positive	4	0	0	4	4	4	80%	no
4171946	47	m	0,35	yes	non-BL	DLBCL	GCB	negative	negative	negative	negative	n.a.	n.a.	n.a.	n.a.	n.a.	n.a.	n.a.	no
4175941	80	f	0,89	no	NA	DLBCL	n.a.	non-IG-MYC pos	positive	negative	positive	4	4	0	4	4	0	90%	no
4176584	65	m	0,65	no	NA	DLBCL	n.a.	negative	negative	positive	negative	4	4	n.a.	1	4	3	90%	no

4176046	61	m	0,87	yes	non-BL	DLBCL	GCB	non-IG-MYC pos	negative	negative	negative	4	4	n.a.	4	3	1	70%	no
4161288	73	m	0,69	yes	non-BL	B-NOS	ABC	negative	negative	positive	negative	4	0	n.a.	4	4	n.a.	50%	no
4164330	44	f	0,76	yes	non-BL	FL	GCB	negative	positive	negative	positive	4	4	0	4	4	0	30%	no
4171586	53	m	0,32	yes	non-BL	DLBCL	GCB	negative	negative	negative	negative	4	4	0	4	4	0	80%	no
4171908*	61	f	0,31	yes	non-BL	FL	TypeIII	negative	positive	negative	positive	4	0	0	4	4	0	0%	no
4177842	75	f	0,54	yes	non-BL	DLBCL	GCB	negative	positive	negative	positive	4	4	n.a.	4	4	3	50%	no
4167925	71	f	0,36	yes	non-BL	DLBCL	GCB	negative	positive	negative	positive	4	4	0	4	4	0	90%	no
4182605	75	f	0,95	yes	BL	DH-BL	TypeIII	non-IG-MYC pos	negative	positive	positive	4	0	n.a.	2	4	3	100%	no
4186613	45	m	0,3	yes	non-BL	FL-DLBCL	GCB	negative	negative	positive	positive	4	1	n.a.	1	4	1	10%	no
4190929	77	f	0,73	yes	non-BL	FL	TypeIII	negative	positive	negative	positive	4	4	n.a.	2	n.a.	n.a.	40%	no

* cases without germline tissue available; f: female; m:male; GEP: gene expression profile; n.a.: not available

Supplementary Table 1B, Clinico-biological characteristics

		Diagnosis				Total
		**FL**	**FL/DLBCL**	**DLBCL**	**others (1 B-NOS, 1 intermediate DLBCL/BL)**	
**Total cases (n=)**		86	17	76	2	181
**Cases thereof with RNA-seq GEP (n=)**		85	17	72	2	176
**GEP classification**	***MYC status****					
**non-mBL**	**single hit**	84	15	59	1 (B-NOS)	159
	**double hit**	1	2	9	0	12
**mIntermediate**	**single hit**	0	0	0	0	0
	**double hit**	0	0	3	0	3
**mBL**	**single hit**	0	0	0	0	0
	**double hit**	0	0	1	1 (intermediate DLBCL/BL)	2

* single hit: cases exhibiting either a *MYC* rearrangement or a *BCL2* or *BCL6* translocation

* double hit: cases exhibiting both a *MYC* rearrangement and a *BCL2* or *BCL6* translocation

Supplementary Table 2. Cohorts and analyses

	Study cohort of 181 nonBL Samples	BL cohort (Lopez et al., Nat Commun) plus one adult BL
Datasets		
whole-genome sequencing (matched tumor and normal tissue)	179	40
whole-genome sequencing tumor only	2	-
RNA-sequencing	176	not studied herein
Analyses		
Structural Variants	x	-
Detection of genomic structural rearrangements	x	-
Detection of copy number aberrations and allelic imbalances	x	-
Cancer cell fractions	x	-
Identification of hallmarks events	x	-
Gene expression based gcBCL classification	x	-
Extraction of clusters of SNVs at high and intermediate mutation density	x	x
Determination of IG switch regions	x	x
Classification of hypermutation clusters into SHM-like and CSR-like	x	x
Hypermutation by proxy	x	x
Unsupervised analysis of mutational signatures	x	x
Supervised analysis of mutational signatures	x	x
Synthetic SHM signature	x	x
Stratified analyses of mutational signatures	x	x
Detection of oncodrivers	x	-
AID enrichment and signature analysis of driver and recurrently mutated genes	x	-

Supplementary Table 3, Variant statistics per subgroup

A Summary mutation statistics

	coding	noncoding	coding / noncoding (%)	snv fraction (%)	snv fraction coding (%)
DLBCL	149	17397	0,9	93,8	94,7
FL	70	7738	0,9	93,5	94,2
FL-DLBCL	150	15658	1	93,2	93,7
DH-mBL/B-NOS	110	10189	1,1	93	92,3
whole cohort	110	12481	0,9	93,6	94,3

Explanations:

coding / noncoding	ratio coding over noncoding mutations (%)
snv fraction	fraction of SNVs in all small variants (%)
snv fraction coding	fraction of SNVs in all coding small variants (%)

B Variant statistics per subgroup

subgroup	BL_leukemia	BL_solid	BL_pleura	DH-BL	DLBCL	FL-DLBCL	FL	B-NOS
number_of_SNVs	1753 +/- 426.989	2359.5 +/- 919.953	2696 +/- 699.787	9237.5 +/- 5312.9	12169 +/- 6948.95	12356 +/- 6092	5515 +/- 2475.94	10277 +/- 0
num_Ka_ROIs	3 +/- 1.4826	4 +/- 1.4826	2 +/- 1.4826	20.5 +/- 9.6369	12 +/- 7.413	9.5 +/- 3.7065	6 +/- 2.9652	7 +/- 0
num_KaIG_ROIs	2 +/- 1.4826	3 +/- 1.4826	1 +/- 1.4826	10.5 +/- 2.2239	6 +/- 2.9652	6 +/- 1.4826	4 +/- 1.4826	6 +/- 0
SNVs_Ka	42 +/- 22.239	61.5 +/- 48.1845	31 +/- 7.413	632 +/- 339.515	391 +/- 330.62	418.5 +/- 320.983	196 +/- 121.573	183 +/- 0
SNVs_Ka_ROIs	32 +/- 19.2738	54.5 +/- 48.9258	31 +/- 0	425 +/- 32.6172	329 +/- 271.316	282 +/- 220.166	178 +/- 87.4734	156 +/- 0
SNVs_KaIG_ROIs	22 +/- 4.4478	49 +/- 40.7715	20 +/- 7.413	283 +/- 42.9954	246 +/- 163.086	212 +/- 160.121	138 +/- 57.8214	147 +/- 0
num_KaNonIG_ROIs	1 +/- 0	1 +/- 1.4826	1 +/- 0	10 +/- 7.413	6 +/- 5.9304	4 +/- 2.9652	2 +/- 1.4826	1 +/- 0
SNVs_KaNonIG_ROIs	10 +/- 7.413	7 +/- 10.3782	6 +/- 7.413	142 +/- 75.6126	78 +/- 90.4386	72.5 +/- 71.9061	30 +/- 37.065	9 +/- 0
SNVs_Ka_nonROIs	6 +/- 5.9304	5 +/- 7.413	5 +/- 1.4826	207 +/- 306.898	44 +/- 48.9258	90 +/- 81.543	16 +/- 16.3086	27 +/- 0
SNVs_Mech_other	7 +/- 2.9652	10.5 +/- 8.8956	6 +/- 7.413	169 +/- 151.225	73 +/- 72.6474	84.5 +/- 72.6474	27 +/- 28.1694	51 +/- 0

SNVs_Mech_CSR	13 +/- 8.8956	12 +/- 14.826	9 +/- 5.9304	120 +/- 96.369	23 +/- 26.6868	16.5 +/- 24.4629	6 +/- 8.8956	18 +/- 0
SNVs_Mech_SHM	19 +/- 7.413	26 +/- 25.9455	12 +/- 14.826	343 +/- 284.659	263 +/- 243.146	302.5 +/- 253.525	151 +/- 85.9908	114 +/- 0
num_Psi_ROIs	2 +/- 1.4826	14 +/- 14.826	13 +/- 13.3434	69.5 +/- 0.7413	65 +/- 29.652	66 +/- 15.5673	63 +/- 38.5476	91 +/- 0
SNVs_Psi	21 +/- 20.7564	133.5 +/- 160.862	100 +/- 81.543	2006.5 +/- 1277.26	2585 +/- 747.23	2536.5 +/- 487.775	1514 +/- 1363.99	2553 +/- 0
SNVs_Psi_ROIs	21 +/- 20.7564	130 +/- 157.156	100 +/- 81.543	1974 +/- 1289.86	2551 +/- 763.539	2510 +/- 523.358	1489 +/- 1335.82	2530 +/- 0
SNVs_Mech_other_ratio	0.21875 +/- 0.147819	0.206019 +/- 0.206633	0.305556 +/- 0.453017	0.240502 +/- 0.110081	0.20339 +/- 0.177996	0.176633 +/- 0.11464	0.117391 +/- 0.107567	0.278689 +/- 0
SNVs_Mech_CSR_ratio	0.309524 +/- 0.0947526	0.193103 +/- 0.263815	0.290323 +/- 0.104951	0.261468 +/- 0.292945	0.0454545 +/- 0.0646053	0.051968 +/- 0.0661141	0.0234742 +/- 0.0348028	0.0983607 +/- 0
SNVs_Mech_SHM_ratio	0.545455 +/- 0.0509176	0.491935 +/- 0.161097	0.333333 +/- 0.4942	0.49803 +/- 0.182864	0.673077 +/- 0.196822	0.749674 +/- 0.152362	0.821739 +/- 0.118024	0.622951 +/- 0
SNVs_Ka_ratio	0.0225352 +/- 0.00879189	0.023942 +/- 0.0156479	0.0089803 +/- 0.00648313	0.0692162 +/- 0.00305529	0.0316448 +/- 0.0148308	0.0317343 +/- 0.0178082	0.0369257 +/- 0.0163741	0.0178068 +/- 0
SNVs_KaROI_ratio	0.0225352 +/- 0.0101656	0.023942 +/- 0.014642	0.0089803 +/- 0.00373351	0.0530708 +/- 0.0269924	0.0269238 +/- 0.0120625	0.025866 +/- 0.0203371	0.032 +/- 0.0144141	0.0151795 +/- 0
SNVs_KaRecurr_ratio	0.918919 +/- 0.120211	0.963008 +/- 0.0548441	0.861111 +/- 0.205917	0.759582 +/- 0.356444	0.872449 +/- 0.108448	0.845434 +/- 0.0839829	0.901515 +/- 0.0833686	0.852459 +/- 0
SNVs_KaROI_IG_ratio	0.703704 +/- 0.051532	0.926662 +/- 0.108732	NA +/- NA	0.671213 +/- 0.152679	0.749226 +/- 0.21613	0.783019 +/- 0.14159	0.842932 +/- 0.152316	0.942308 +/- 0
ROIs_Ka_ratio	0.75 +/- 0.12355	0.816667 +/- 0.27181	NA +/- NA	0.543651 +/- 0.147083	0.5 +/- 0.2471	0.569444 +/- 0.214742	0.714286 +/- 0.1765	0.857143 +/- 0
SNVs_Psi_ratio	0.0113698 +/- 0.00954826	0.0548465 +/- 0.0530083	0.037092 +/- 0.0249939	0.213103 +/- 0.0157037	0.222833 +/- 0.100484	0.199039 +/- 0.082989	0.215413 +/- 0.135348	0.248419 +/- 0
SNVs_PsiROI_ratio	0.0113698 +/- 0.00954826	0.0548465 +/- 0.0530083	0.037092 +/- 0.0249939	0.208542 +/- 0.0196916	0.220361 +/- 0.100424	0.198478 +/- 0.0817735	0.215413 +/- 0.123929	0.246181 +/- 0
SNVs_Ka_Psi_ratio	1.75 +/- 2.01798	0.518838 +/- 0.485591	0.133333 +/- 0.0844766	0.326087 +/- 0.0383666	0.166579 +/- 0.110377	0.156423 +/- 0.109835	0.165146 +/- 0.099458	0.0716804 +/- 0
SNVs_KaROI_PsiROI_ratio	1.6875 +/- 2.17958	0.41882 +/- 0.394288	0.0763547 +/- 0.113203	0.261105 +/- 0.154089	0.137012 +/- 0.0841773	0.105967 +/- 0.090327	0.148776 +/- 0.102962	0.0616601 +/- 0
num_KaROI_PsiROI_ratio	1 +/- 1.15313	0.216374 +/- 0.143895	0.0810811 +/- 0.107881	0.294306 +/- 0.135521	0.173333 +/- 0.144666	0.168775 +/- 0.11367	0.112903 +/- 0.0739632	0.0769231 +/- 0

Explanations:

number_of_SNVs	median number of SNVs per subgroup with MAD (median absolute deviation)
num_Ka_ROIs	median number of kataegis regions affected per subgroup with MAD
num_KaIG_ROIs	median number of kataegis regions inside the IG loci affected per subgroup with MAD
SNVs_Ka	median number of SNVs in kataegis clusters per subgroup with MAD
SNVs_Ka_ROIs	median number of SNVs in kataegis regions (and thus in RECURRENT kataegis clusters) per subgroup with MAD

SNVs_KaIG_ROIs	median number of SNVs in kataegis regions (and thus in RECURRENT kataegis clusters) inside the IG loci per subgroup with MAD
num_KaNonIG_ROIs	median number of kataegis regions outside the IG loci affected per subgroup with MAD
SNVs_KaNonIG_ROIs	median number of SNVs in kataegis regions (and thus in RECURRENT kataegis clusters) outside the IG loci per subgroup with MAD
SNVs_Ka_nonROIs	median number of SNVs in NON-RECURRENT kataegis clusters per subgroup with MAD
SNVs_Mech_other	median number of SNVs in kataegis clusters of type "other" per subgroup with MAD
SNVs_Mech_CSR	median number of SNVs in CSR-like kataegis clusters per subgroup with MAD
SNVs_Mech_SHM	median number of SNVs in SHM-like kataegis clusters per subgroup with MAD
num_Psi_ROIs	median number of psichales regions affected per subgroup with MAD
SNVs_Psi	median number of SNVs in psichales clusters per subgroup with MAD
SNVs_Psi_ROIs	median number of SNVs in psichales regions (and thus in RECURRENT kataegis clusters) per subgroup with MAD
SNVs_Mech_other_ratio	median fraction of SNVs in kataegis clusters of type "other" among the number of SNVs in kataegis clusters per subgroup with MAD
SNVs_Mech_CSR_ratio	median fraction of SNVs in CSR-like kataegis clusters among the number of SNVs in kataegis clusters per subgroup with MAD
SNVs_Mech_SHM_ratio	median fraction of SNVs in SHM-like kataegis clusters among the number of SNVs in kataegis clusters per subgroup with MAD
SNVs_Ka_ratio	median fraction of the number of SNVs in kataegis clusters among the total number of SNVs per subgroup with MAD
SNVs_KaROI_ratio	median fraction of the number of SNVs in kataegis regions (and thus in RECURRENT kataegis clusters) among the total number of SNVs per subgroup with MAD
SNVs_KaRecurr_ratio	median fraction of the number of SNVs in kataegis regions (and thus in RECURRENT kataegis clusters) among the number of SNVs in all kataegis clusters per subgroup with MAD
SNVs_KaROI_IG_ratio	median fraction of the number of SNVs in kataegis regions inside the IG loci among the number of SNVs in kataegis regions (and thus in RECURRENT kataegis clusters) per subgroup with MAD
ROIs_Ka_ratio	median fraction of the number of kataegis regions in the IG loci among all kataegis regions per subgroup with MAD
SNVs_Psi_ratio	median fraction of the number of SNVs in psichales clusters among the total number of SNVs per subgroup with MAD
SNVs_PsiROI_ratio	median fraction of the number of SNVs in psichales regions (and thus in RECURRENT psichales clusters) among the total number of SNVs per subgroup with MAD
SNVs_Ka_Psi_ratio	median ratio of the number of SNVs in kataegis clusters divided by the number of SNVs in psichales clusters per subgroup with MAD
SNVs_KaROI_PsiROI_ratio	median ratio of the number of SNVs in kataegis regions (and thus in RECURRENT kataegis clusters) divided by the number of SNVs in psichales regions (and thus in RECURRENT psichales clusters) per subgroup with MAD
num_KaROI_PsiROI_ratio	median ratio of the number of kataegis regions divided by the number of psichales regions per subgroup with MAD

C Variant statistics per subgroup for DLBCL

ABCGCB	ABC	GCB	TypeIII	n.d.
number of cases	23	37	11	4
number_of_SNVs	8978 +/- 6986.01	12477.5 +/- 7338.13	12909.5 +/- 5729.51	16800.5 +/- 1968.15
num_Ka_ROIs	8 +/- 5.9304	13.5 +/- 9.6369	11 +/- 7.413	16 +/- 8.8956
num_KaIG_ROIs	5 +/- 2.9652	6 +/- 2.9652	7 +/- 2.9652	8.5 +/- 3.7065
SNVs_Ka	270 +/- 206.081	470 +/- 358.789	494 +/- 345.446	637.5 +/- 9.6369
SNVs_Ka_ROIs	249 +/- 174.947	358 +/- 225.355	426.5 +/- 354.341	507 +/- 17.7912
SNVs_KaIG_ROIs	222 +/- 177.912	254.5 +/- 123.797	357.5 +/- 259.455	326.5 +/- 74.8713
num_KaNonIG_ROIs	3 +/- 4.4478	7.5 +/- 8.1543	5.5 +/- 5.9304	7.5 +/- 5.1891
SNVs_KaNonIG_ROIs	38 +/- 56.3388	91 +/- 83.0256	85.5 +/- 100.817	180.5 +/- 92.6625
SNVs_Ka_nonROIs	26 +/- 20.7564	64 +/- 72.6474	49 +/- 49.6671	130.5 +/- 27.4281
SNVs_Mech_other	43 +/- 48.9258	90.5 +/- 81.543	117.5 +/- 77.8365	94 +/- 31.1346
SNVs_Mech_CSR	28 +/- 29.652	20 +/- 22.239	29.5 +/- 37.8063	55 +/- 5.9304
SNVs_Mech_SHM	188 +/- 131.951	324 +/- 273.54	274.5 +/- 317.276	488.5 +/- 46.7019
num_Psi_ROIs	62 +/- 37.065	65.5 +/- 30.3933	73 +/- 26.6868	59 +/- 8.8956
SNVs_Psi	2571 +/- 1264.66	2522.5 +/- 607.125	2598.5 +/- 651.603	4313.5 +/- 779.106
SNVs_Psi_ROIs	2521 +/- 1338.79	2522.5 +/- 597.488	2589 +/- 665.687	4313.5 +/- 779.106
SNVs_Mech_other_ratio	0.128205 +/- 0.0769014	0.238813 +/- 0.201166	0.27579 +/- 0.179998	0.147802 +/- 0.0510729
SNVs_Mech_CSR_ratio	0.0680851 +/- 0.0692635	0.0325237 +/- 0.0482196	0.0392718 +/- 0.0526422	0.0863475 +/- 0.0106079
SNVs_Mech_SHM_ratio	0.717391 +/- 0.213821	0.65629 +/- 0.206666	0.718736 +/- 0.183282	0.76585 +/- 0.0616807
SNVs_Ka_ratio	0.0301586 +/- 0.0115146	0.0306745 +/- 0.0181282	0.0331236 +/- 0.0173698	0.0381529 +/- 0.00389595
SNVs_KaROI_ratio	0.027093 +/- 0.0109481	0.0237205 +/- 0.0132039	0.0312556 +/- 0.0186984	0.0304241 +/- 0.0046231
SNVs_KaRecurr_ratio	0.894799 +/- 0.0753113	0.846 +/- 0.132064	0.88834 +/- 0.0843561	0.795569 +/- 0.0399341
SNVs_KaROI_IG_ratio	0.878187 +/- 0.1806	0.700707 +/- 0.183481	0.810383 +/- 0.227881	0.646704 +/- 0.170369
ROIs_Ka_ratio	0.625 +/- 0.344175	0.459936 +/- 0.19768	0.535714 +/- 0.231955	0.55 +/- 0.07413
SNVs_Psi_ratio	0.193268 +/- 0.112735	0.224571 +/- 0.105024	0.19919 +/- 0.0825156	0.255874 +/- 0.0163987
SNVs_PsiROI_ratio	0.193268 +/- 0.112735	0.221356 +/- 0.10207	0.198663 +/- 0.0796763	0.255874 +/- 0.0163987
SNVs_Ka_Psi_ratio	0.12483 +/- 0.0875607	0.17124 +/- 0.120341	0.19497 +/- 0.153448	0.149832 +/- 0.0248286

SNVs_KaROI_PsiROI_ratio	0.120068 +/- 0.04899	0.13895 +/- 0.0964113	0.174511 +/- 0.156131	0.119653 +/- 0.0257363
num_KaROI_PsiROI_ratio	0.150442 +/- 0.0828001	0.220362 +/- 0.198217	0.27159 +/- 0.259626	0.28447 +/- 0.193663

Explanations:

number_of_SNVs	median number of SNVs per subgroup with MAD (median absolute deviation)
num_Ka_ROIs	median number of kataegis regions affected per subgroup with MAD
num_KaIG_ROIs	median number of kataegis regions inside the IG loci affected per subgroup with MAD
SNVs_Ka	median number of SNVs in kataegis clusters per subgroup with MAD
SNVs_Ka_ROIs	median number of SNVs in kataegis regions (and thus in RECURRENT kataegis clusters) per subgroup with MAD
SNVs_KaIG_ROIs	median number of SNVs in kataegis regions (and thus in RECURRENT kataegis clusters) inside the IG loci per subgroup with MAD
num_KaNonIG_ROIs	median number of kataegis regions outside the IG loci affected per subgroup with MAD
SNVs_KaNonIG_ROIs	median number of SNVs in kataegis regions (and thus in RECURRENT kataegis clusters) outside the IG loci per subgroup with MAD
SNVs_Ka_nonROIs	median number of SNVs in NON-RECURRENT kataegis clusters per subgroup with MAD
SNVs_Mech_other	median number of SNVs in kataegis clusters of type "other" per subgroup with MAD
SNVs_Mech_CSR	median number of SNVs in CSR-like kataegis clusters per subgroup with MAD
SNVs_Mech_SHM	median number of SNVs in SHM-like kataegis clusters per subgroup with MAD
num_Psi_ROIs	median number of psichales regions affected per subgroup with MAD
SNVs_Psi	median number of SNVs in psichales clusters per subgroup with MAD
SNVs_Psi_ROIs	median number of SNVs in psichales regions (and thus in RECURRENT kataegis clusters) per subgroup with MAD
SNVs_Mech_other_ratio	median fraction of SNVs in kataegis clusters of type "other" among the number of SNVs in kataegis clusters per subgroup with MAD
SNVs_Mech_CSR_ratio	median fraction of SNVs in CSR-like kataegis clusters among the number of SNVs in kataegis clusters per subgroup with MAD
SNVs_Mech_SHM_ratio	median fraction of SNVs in SHM-like kataegis clusters among the number of SNVs in kataegis clusters per subgroup with MAD
SNVs_Ka_ratio	median fraction of the number of SNVs in kataegis clusters among the total number of SNVs per subgroup with MAD
SNVs_KaROI_ratio	median fraction of the number of SNVs in kataegis regions (and thus in RECURRENT kataegis clusters) among the total number of SNVs per subgroup with MAD
SNVs_KaRecurr_ratio	median fraction of the number of SNVs in kataegis regions (and thus in RECURRENT kataegis clusters) among the number of SNVs in all kataegis clusters per subgroup with MAD
SNVs_KaROI_IG_ratio	median fraction of the number of SNVs in kataegis regions inside the IG loci among the number of SNVs in kataegis regions (and thus in RECURRENT kataegis clusters) per subgroup with MAD
ROIs_Ka_ratio	median fraction of the number of kataegis regions in the IG loci among all kataegis regions per subgroup with MAD
SNVs_Psi_ratio	median fraction of the number of SNVs in psichales clusters among the total number of SNVs per subgroup with MAD

SNVs_PsiROI_ratio	median fraction of the number of SNVs in psichales regions (and thus in RECURRENT psichales clusters) among the total number of SNVs per subgroup with MAD
SNVs_Ka_Psi_ratio	median ratio of the number of SNVs in kataegis clusters divided by the number of SNVs in psichales clusters per subgroup with MAD
SNVs_KaROI_PsiROI_ratio	median ratio of the number of SNVs in kataegis regions (and thus in RECURRENT kataegis clusters) divided by the number of SNVs in psichales regions (and thus in RECURRENT psichales clusters) per subgroup with MAD
num_KaROI_PsiROI_ratio	median ratio of the number of kataegis regions divided by the number of psichales regions per subgroup with MAD

Supplemental Table 4. Clusters of mutation density

Suppl. Table 4A: p-values and effect strength of the Wilcoxon rank sum tests of replication time averaged over cell lines vs. psichales in the different entities. Abbreviations: p-val.: p-value; corr. p-val.: corrected p-value; diff.: difference in Repli-seq scores between psichales clusters and background; conf. int.: confidence interval for the difference. Note that both the difference values and the confidence intervals are in units of RepliSeq scores. These results are based on distributions shown in Extended Data Figure 4A.

Entity	BRCA-EU	BRCA-UK	BRCA-US	CLLE-ES	DLBC-US	OV-AU	OV-US	SARC-US
p-val.	< 2.2×10-16	< 2.2×10-16	< 2.2×10-16	< 2.2×10-16	< 2.2×10-16	< 2.2×10-16	< 2.2×10-16	< 2.2×10-16
corr. p-val.	< 2.2×10-16	< 2.2×10-16	< 2.2×10-16	< 2.2×10-16	< 2.2×10-16	< 2.2×10-16	< 2.2×10-16	< 2.2×10-16
diff.	3,76	1,14	5,24	2,59	21	8,75	9,75	8,72
conf. int.	[3.62 - 3.90]	[1.02 - 1.27]	[5.14 - 5.33]	[2.45 - 2.73]	[20.79 - 21.22]	[8.65 - 8.85]	[9.61 - 9.89]	[8.50 - 8.94]

Suppl. Table 4B: Comparison of p-values and effect strength of the Wilcoxon rank sum tests in the breast cancer cell line MCF7 against replication time averaged over cell lines vs. psichales in the breast cancer entities. Abbreviations as in Extended Data Table 4A. Also in analogy to Extended Data Table 4A, both the difference values and the confidence intervals are in units of RepliSeq scores. These results are based on distributions shown in Extended Data Figure 4C.

Cell line	MCF7			average over cell lines		
Quantity	p-val.	diff.	conf. int	p-val.	diff.	conf. int
BRCA-EU	< 2.2×10-16	4,09	[3.94 - 4.24]	< 2.2×10-16	3,76	[3.62 - 3.90]
BRCA-UK	< 2.2×10-16	1,16	[1.02 - 1.29]	< 2.2×10-16	1,14	[1.02 - 1.27]
BRCA-US	< 2.2×10-16	5,75	[5.65 - 5.85]	< 2.2×10-16	5,24	[5.14 - 5.33]

Suppl. Table 4C: Comparison of p-values and effect strength of the Wilcoxon rank sum tests in the lymphoblastoid cell lines GM06990 and GM12878 against replication time averaged over cell lines vs. psichales in the B-cell derived entities. Abbreviations as in Extended Data Tables 4A and 4B. Also in analogy to Extended Data Tables 4A and 4B, both the difference values and the confidence intervals are in units of RepliSeq scores. These results are based on distributions shown in Extended Data Figure 4D.

Cell line	GM06990			GM12878			average over cell lines		
Quantity	p-val.	diff.	conf. int	p-val.	diff.	conf. int	p-val.	diff.	conf. int
CLLE-ES	< 2.2×10-16	2,88	[2.73 - 3.03]	< 2.2×10-16	3,54	[3.36 - 3.73]	< 2.2×10-16	2,59	[2.45 - 2.73]
DLBC-US	< 2.2×10-16	24,23	[23.98 - 24.49]	< 2.2×10-16	27,77	[27.50 - 28.04]	< 2.2×10-16	21	[20.79 - 21.22]

Supplemental Table 5. Cataegis analysis

a) Decomp_cluaster_vs_distTSScat

Distance to closest TSS	close	intermediate	far
other	682	486	616
CSR_like	347	70	10
SHM_like	1547	345	429

Explanations for the categories of distances to the closest transcription start site (TSS) (analogous to the definitions in Extended Data Figure 9F):

close	less than 2.5 kb
intermediate	between 2.5 kb and 20 kb
far	more than 20 kb

b) decomp_cluster_vs_maxState

	Enhancer	Heterochromatin	PoisedPromoter	Promoter	Transcription
other	198	914	40	481	151
CSR_like	67	13	9	317	21
SHM_like	246	625	118	1236	96

c) dist_TSS_cat_vs_maxState

	Enhancer	Heterochromatin	PoisedPromoter	Promoter	Transcription
close	292	186	166	1816	116
intermediate	172	408	1	199	121
far	47	958	0	19	31

Explanations for the categories of distances to the closest transcription start site (TSS) (analogous to the definitions in Extended Data Figure 9F):

close	less than 2.5 kb
intermediate	between 2.5 kb and 20 kb
far	more than 20 kb

d) dist_TSS_vs_decomp_cluster

Statistical measures of the distribution of distances to the closest transcription start site (TSS)

	median	mad
other	7417.5	105.909.531
CSR_like	559.5	4.937.058
SHM_like	726.5	8.280.321

e) decomp_cluster_vs_recurrency

	non-recurrent kataegis clusters	recurrent kataegis clusters
other	1191	593
CSR_like	48	379
SHM_like	836	1485

f) dist_TSS_cat_vs_recurrency

	non-recurrent kataegis clusters	recurrent kataegis clusters
close	434	2142
intermediate	625	276
far	1016	39

Explanations for the categories of distances to the closest transcription start site (TSS) (analogous to the definitions in Extended Data Figure 12F):

close	less than 2.5 kb
intermediate	between 2.5 kb and 20 kb
far	more than 20 kb

g) maxState_vs_recurrency

	non-recurrent kataegis clusters	recurrent kataegis clusters
Enhancer	180	331
Heterochromatin	1427	125
PoisedPromoter	11	156
Promoter	245	1789
Transcription	212	56

h) pairWil_dist_TSS_vs_class

Table of pairwise posthoc tests for the distance to the closest TSS (Wilcoxon rank sum tests)

	other	CSR_like
CSR_like	3,29E-46	NA
SHM_like	3,93E-50	2,81E+08

i) pairWil_dist_TSS_vs_max_maxState

Table of pairwise posthoc tests for the distance to the closest TSS (Wilcoxon rank sum tests)

	Enhancer	Heterochromatin	PoisedPromoter	Promoter
Heterochromatin	1,71E-105	NA	NA	NA
PoisedPromoter	1,55E-07	9,48E-71	NA	NA
Promoter	5,15E-09	0	2,91E+05	NA
Transcription	1,83E+09	3,69E-45	1,64E-23	9,53E-37

j) comb_SHM_CSR_vs_distTSScar

Fisher test for the combined analysis of SHM-like and CSR-like profiles vs. the categorized distance to the closest TSS

p-value	3,30E-72

Supplementary Table 7, HbP characteristics

Suppl. Table 7A-C: Influence of TSS proximity on subject or object state in HbP relationships. (A) All HbP interactions. (B) Only HbP interactions involving IG loci. (C) Only HbP interactions without IG locus overlap.

			Object		
			close	far	Row sums
A) All interactions	Subject	close	271	164	435
		far	34	27	61
	Column sums		305	191	496
B) IG interactions	Subject	close	181	116	297
		far	6	1	7
	Column sums		187	117	304
C) Non-IG interactions	Subject	close	90	48	138
		far	28	26	54
	Column sums		118	74	192

Suppl. Table 7D: Test statistics (Wilcoxon rank sum test) for differences in the abundance fractions of the different chromatin states between subject and object regions in HbP relationships.

Grouping	Chromatin state	W statistic	p-value	BH-corrected p-value
HbP relationships involving all kataegis regions	Heterochromatin	110,118	4.56×10-10	1.82×10-9
	Enhancer	146,655	5.55×10-8	1.66×10-7
	Transcription	144,106.5	3.42×10-10	1.71×10-9
	Promoter	134,478.5	9.52×10-3	1.90×10-2
	Poised Promoter	120,367	0.101	0.101
Only HbP relationships involving the IG loci	Heterochromatin	39,088	1.11×10-10	4.43×10-10
	Enhancer	55,762	6.61×10-6	1.32×10-5
	Transcription	59,560.5	4.77×10-13	2.38×10-12
	Promoter	56,432.5	1.55×10-6	4.68×10-6
	Poised Promoter	43,320	9.71×10-6	1.32×10-5
HbP relationships involving only non-IG loci	Heterochromatin	17,930	0.2	0.39
	Enhancer	21,216	3.69×10-3	1.84×10-2
	Transcription	17,476	6.81×10-3	2.73×10-2
	Promoter	17,410	0.31	0.39
	Poised Promoter	19,228	0.087	0.26

Supplementary Table 9, Mutational signatures

Signature	Colour	Mutational Mechanism	Category	Comment
AC1	green	spontaneous deamination	clock-like	previously described in lymphoma
AC2	pink	APOBEC	phys. endo. mut.	previously described in lymphoma
AC3	gold	HRR defect	DNA repair defect	
AC6	red	MMR defect	DNA repair defect	
AC8	orange	possibly HRR defect	possibly DNA repair defect	
AC9	brown	previously POL η, implicated in AID activity	phys. endo. mut.	previously described in lymphoma
AC10	purple	activity of error-prone POL ε	phys. endo. mut.	
AC12	blue	unknown	unknown	
AC17	turquoise	unknown	unknown	previously described in lymphoma
AC26	ocre	MMR defect	DNA repair defect	
AC28	dark blue	unknown	unknown	
L1	carmen	CSR	new signature	
L2	lightbrown	SHM	new signature	
L3	black	unknown	new signature	very similar to AC17

Supplemental Table 10. Stratified analysis of mutational signatures. Numbers of SNVs in the different strata with different stratification axes

Stratification axis	Stratum	Number of SNVs	Matching Figure
Cellular fractions (corrected allele frequency)	early	1832664	Suppl. Figure S12D
	late	64067	
	unknown	236610	
Replication timing (encoded by RepliSeq scores)	early	297675	Main Figure 4E
	intermediate	822194	
	late	1011923	
	undetermined	1549	
Mutation density	high	650387	Suppl. Figure S12E
	intermediate	245894	
	background	1237060	
Breakpoint proximity	Less than 100 kb	80477	Main Figure 4D
	Between 100 kp and 1 Mb	171221	
	More than 1 Mb	1881643	
Subregions in IG loci	IG_const_noSwitch	1058	Main Figure 4C
	IG_const_switch	7591	
	IG_VDJ	41267	
	none	2083425	
Distance to TSS	close	272755	Suppl. Figure S12F
	intermediate	1169463	
	far	691123	
Chromatin state	Enhancer	55218	Main Figure 4D
	Heterochromatin	1837471	
	PoisedPromoter	12441	
	Promoter	80492	
	Transcription	144922	
Hypermutation catergory	None	1685331	Main Figure 4G
	Psichales	374680	
	Kataegis_other	17215	
	Kataegis CSR-like	5748	
	Kataegis SHM-like	50367	

Supplementary Table 11, Driver genes identified by IntOGen

The cohort column indicates whether the gene has been identified in the full cohort, or the DLBCL or FL specific analysis.

GENE	Full cohort	DLBCL	FL
ACTB	yes	yes	yes
ACTG1	yes		yes
ADAMTS1		yes	
ANKRD12		yes	
ARID1A	yes		yes
ATP6AP1	yes		yes
ATP6V1A	yes		
ATP6V1B2	yes		yes
B2M	yes	yes	yes
BCL2			yes
BCL6	yes	yes	
BCL7A	yes		yes
BRAF	yes	yes	
BTG2	yes		
CADPS2	yes	yes	
CARD11	yes		
CCND3	yes		
CD58	yes		
CD74	yes		
CD79A	yes		
CD79B	yes	yes	
CD83	yes	yes	
CDC42BPB			yes
CDKN2A	yes		
CNOT2	yes	yes	
CPNE8	yes		yes
CREBBP	yes	yes	yes
CXCR4			yes
CYLD	yes		
DHX15			yes
DHX16		yes	
DMXL1	yes		
DNM2		yes	
EBF1	yes		yes
EEF1A1	yes	yes	yes
EP300	yes	yes	yes
EZH2	yes	yes	yes
FAM136A	yes		
FAS	yes	yes	
FBXO11	yes	yes	
FBXW7	yes		
FCGR2B	yes		
FES	yes		
FOXO1	yes		yes
FUBP1	yes		
FZR1	yes		yes
GAK	yes	yes	
GNA11	yes		
GNA13	yes	yes	yes
GNAI2	yes	yes	yes
GRB2	yes	yes	
HIST1H2AC	yes		
HIST1H2BD	yes		
HLA-A	yes	yes	
HLA-B	yes	yes	
HLA-DMB	yes	yes	
HLA-DQA1	yes	yes	
ID2	yes		
IKBKE	yes	yes	
IL4R	yes	yes	
INO80	yes		
IRF1	yes	yes	
IRF8	yes		yes
JHDM1D	yes		
JUNB	yes	yes	
JUP			yes
LAPTM5	yes		yes
LRP12		yes	
MEF2C	yes		yes
MGEA5			yes
KMT2D	yes	yes	yes
MRPL13	yes		
MYCBP2			yes
MYD88	yes	yes	
MYH9	yes		
NFKBIA	yes	yes	
NFKBIE	yes	yes	
NOTCH2	yes	yes	
NR2F2	yes	yes	
PDS5B			yes
PIM1	yes	yes	
PNPO	yes	yes	
PPP3CA	yes		
PPP4C	yes		yes
PRKDC	yes		yes
PTPN6	yes		
RAC2	yes	yes	
RBM6	yes		yes
RRAGC	yes		yes
SGK1	yes	yes	
SIAH2		yes	
SIN3A		yes	
SLC34A2	yes	yes	
SOCS1	yes	yes	yes
SPEN	yes		
STAT3	yes		
STAT6	yes		yes
SYNCRIP	yes		
TAF1	yes	yes	
TBL1XR1	yes	yes	
TET2	yes	yes	
TMEM30A	yes		
TNFAIP3	yes	yes	
TNFRSF14	yes	yes	yes
TOX	yes		
TP53	yes	yes	yes
TPP1	yes		yes
TRAF6	yes	yes	
UBE2A	yes	yes	
UGGT2	yes		
UNC5B	yes	yes	
VMA21	yes		yes
WAPAL	yes		
WNK1	yes	yes	
XPO1	yes	yes	
ZFP36L1	yes	yes	
ZNF217		yes	
ZNF292		yes	

Supplemental Table 12. NFM consensus clustering

Suppl Table 12A: Pairwise post-hoc tests (Wilcoxon rank sum test p-values) for total mutational load between the different consensus clusters.

	TP53-like	B2M-like	PIM1-like	BCL6-like	PAX5-like	BCL2-like	CSMD1-like	SOCS1-like
B2M-like	1	-	-	-	-	-	-	-
PIM1-like	1	1	-	-	-	-	-	-
BCL6-like	1	1	1	-	-	-	-	-
PAX5-like	1	1	1	1	-	-	-	-
BCL2-like	1.7×10-5	1.7×10-5	1.7×10-5	1.7×10-5	1.7×10-5	-	-	-
CSMD1-like	1	1	1	1	1	0.99	-	-
SOCS1-like	1	1	1	1	1	3.2×10-3	1	-
MYD88-like	5.4×10-2	5.6×10-2	2.5×10-2	1	1.9×10-2	1	1	0.86

Suppl Table 12B: Pairwise post-hoc tests (Wilcoxon rank sum test p-values) for the fraction of the aberrant genome between the different consensus clusters.

	TP53-like	B2M-like	PIM1-like	BCL6-like	PAX5-like	BCL2-like	CSMD1-like	SOCS1-like
B2M-like	1	-	-	-	-	-	-	-
PIM1-like	1	1	-	-	-	-	-	-
BCL6-like	1	1	0.49	-	-	-	-	-
PAX5-like	1	1	0.69	1	-	-	-	-
BCL2-like	9.84×10-2	1	5.6×10-3	1	1	-	-	-
CSMD1-like	1	1	1	1	1	1	-	-
SOCS1-like	1	1	1	0.54	0.72	1.33×10-2	1	-
MYD88-like	1	1	1	1	1	0.69	1	1

Suppl. Table 12C: Cross-over of the DLBCL cases between the consensus clustering restricted to DLBCLs (rows) and the consensus clustering of the whole gcBCL cohort (columns). Fields highlighted in green indicate predominant matching of corresponding consensus clusters (on the diagonal), whereas fields highlighted in orange indicate predominant matching of off-diagonal fields (high level of cross-over).

	BCL2-like	BCL6-like	MYD88-like	TP53-like	B2M-like	SOCS1-like	CSMD1-like	PIM1-like	PAX5-like
BCL2-like	1	0	0	1	3	1	4	0	0
BCL6-like	0	3	1	2	0	1	2	5	2
MYD88-like	0	8	10	1	0	2	0	3	2
TP53-like	0	4	0	6	1	6	0	0	2

Supplemental Table 13: Position of variant as compared to driver gene vs. class of kataegis cluster.

A) Only variants annotated to driver genes by ANNOVAR (14774 SNVs)

			SNV classification				Row sums
				Class of kataegis cluster			
	orientation	distance to TSS	Single SNVs	other	CSR-like	SHM-like	
Position of variant as compared to driver gene	upstream	close	139	178	1	691	1009
		far	0	0	0	0	0
	Gene body	close	1282	687	241	3734	5944
		far	5956	569	59	1199	7783
	downstream	close	0	0	0	0	0
		far	38	0	0	0	38
Column sums			7415	1434	301	5624	14774

B) Also considering variants which are intergenic and annotated to antisense transcripts (26702 SNVs)

			SNV classification				Row sums
				Class of kataegis cluster			
	orientation	distance to TSS	Single SNVs	other	CSR-like	SHM-like	
Position of variant as compared to driver gene	upstream	close	324	228	7	850	1409
		far	5787	89	6	58	5940
	Gene body	close	1502	1002	293	4885	7682
		far	6313	585	59	1204	8161
	downstream	close	1	0	0	18	19
		far	3421	43	6	21	3491
Column sums			17348	1947	371	7036	26702
